# The Dallas Lifespan Brain Study: A Comprehensive Adult Lifespan Data Set of Brain and Cognitive Aging

**DOI:** 10.1038/s41597-025-04847-7

**Published:** 2025-05-26

**Authors:** Denise C. Park, Joseph P. Hennessee, Evan T. Smith, Micaela Y. Chan, Xi Chen, Marianna Dakanali, Michelle E. Farrell, Peiying Liu, Hanzhang Lu, Neil Rofsky, Xiankai Sun, Carol Tamminga, William Moore, Kristen M. Kennedy, Karen Rodrigue, Gagan S. Wig

**Affiliations:** 1 Center for Vital Longevity & Department of Psychology, University of Texas at Dallas, Dallas, TX 75235 USA; 2 Department of Psychiatry, University of Texas Southwestern Medical Center, Dallas, TX 75390 USA; 3 Department of Psychology, SUNY Stony Brook University, 100 Nicolls Road, Stony Brook, NY 11794 USA; 4 Department of Radiology, University of Texas Southwestern Department, Dallas, TX 75390 USA; 5 Department of Neurology, Massachusetts General Hospital, Harvard Medical School, Boston, MA USA; 6 Department of Diagnostic Radiology & Nuclear Medicine, University of Maryland School of Medicine, Baltimore, MD 21201 USA; 7 Russell H. Morgan Department of Radiology, Johns Hopkins University School of Medicine, Baltimore, MD 21205 USA; 8 Department of Radiology and Nuclear Medicine, Icahn School of Medicine at Mount Sinai, New York, NY 10029 USA

**Keywords:** Neural ageing, Predictive markers, Alzheimer's disease, Risk factors

## Abstract

The Dallas Lifespan Brain Study (DLBS) was designed to integrate brain and cognition across the adult lifespan. Participants (n = 464) were between age 21 and 89 years at time of first assessment and returned approximately every 3.5–5 years for a second (n = 338) and third epoch (n = 224) of data collection. The three epochs included a comprehensive neuropsychological battery, questionnaires that assessed physical health, psychosocial status, and brain health, structural MRI scans (including T1-weighted imaging and diffusion-weighted imaging), a hypercapnia scan, an arterial spin labeling scan, and four functional fMRI scans. Additionally, measures of amyloid and tau were collected with AV-45 (Florbetapir) and AV-1451 (Flortaucipir). Key innovations were robust sampling of middle-aged participants and inclusion of PET data for amyloid and tau in a cognitively normal sample. This large data set has recently been published on OpenNeuro.org open-access and provides the opportunity for researchers to test many hypotheses about brain and cognition across human adulthood, including longitudinal hypotheses, with these data across a multi-year span.

## Background & Significance

There is a large body of literature demonstrating convincingly that cognition changes with adult age. Most of this work is cross-sectional and/or behavioral in nature, limiting the conclusions that can be drawn about brain and cognitive aging within individuals. There are, however, a growing number of studies that relate brain connectomics to the dynamic nature of the aging brain which may shift from being driven by anatomics to a brain that is more based on topology^1,2^. The present set of data represent an empirical instantiation of the theories presented by Park *et al*.^3^ and Park & Reuter Lorenz^4–6^, integrating an extensive behavioral battery with multi-modal neuroimaging measures and named “The Dallas Lifespan Brain Study (DLBS)”. This extensive dataset has a broad scope and allows for the testing of many different hypotheses that go well beyond the Scaffolding Theory of Aging and Cognition (STAC) model. Adult participants ranging in age from 21 to 89 years (age of first assessment) were invited for testing three times at roughly four-year intervals. At each of these testing epochs, participants completed two extended sessions of cognitive assessment over the span of two days, and all received a MRI scan on the third day. MRI scanning included structural scans and four functional imaging tasks; they were also offered the opportunity to participate in a hypercapnia scan. Shortly after the study began in 2010, ligands that measured brain amyloid and tau deposition became available and were added to the study, early on.

**Fig. 1:**
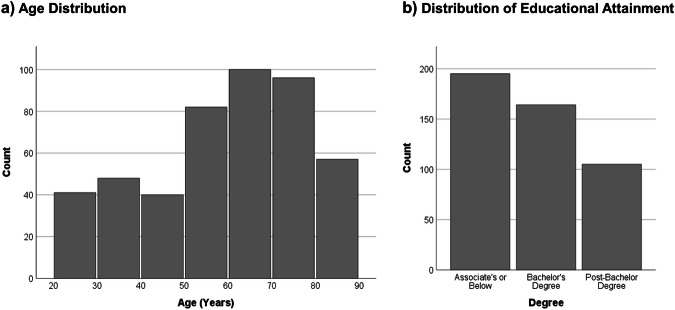
Age, and educational attainment, collected during epoch 1 of the DLBS. (**a**) Histogram of age (5-year bins). (**b**) Educational attainment by type of degree.

A total of 464 subjects participated in the first time point of data collection; all participants were invited back for both behavioral and brain imaging assessments, every 3–5 years. A total of 338 subjects returned for epoch two of data collection, and a further 224 participants returned for a third epoch of testing. Notably, this resulted in longitudinal data being available for every decade of adult life from the twenties through the eighties at time of first assessment (Fig. 1).

Participants completed a total of 24 cognitive tasks, organized by major constructs (see Table 1) that can be considered the building blocks of cognition. Overall, there were multiple measures of each construct, which included speed of processing, working memory, episodic memory, reasoning, verbal fluency and vocabulary. The use of multiple indicators of the same construct strengthens the construct and improves reliability - the cognitive measures are summarized in Table 1. Note that some tasks were discontinued or added as the study progressed (e.g., NIH Toolbox was not available for use in labs until epoch two), and availability of a given task at each epoch of testing is also summarized in Table 1. A complete definition of every construct along with detailed descriptions of associated tasks are provided in *The Keys to the Kingdom*, the data dictionary for this dataset, available in the supplementary data for this manuscript and in the public repository of these data on OpenNeuro.org.

**Table 1:** Availability of tasks by assessment epochs.

Assessment	Cognitive Assessments			
	Construct	E1	E2	E3
CANTAB Verbal Recognition Memory^46^	Episodic Memory	✔	✔	✔
Hopkin’s Verbal Learning^47^	Episodic Memory	✔	✔	✔
NIH Toolbox Picture Sequence Memory^48^	Episodic Memory	X	✔	✔
Wechsler Memory Scale Logical Memory^49^	Episodic Memory	X	✔	✔
Woodcock-Johnson Memory for Names^50^	Episodic Memory	✔	✔	✔
CANTAB Stockings of Cambridge^46^	Reasoning	✔	✔	✔
ETS Letter Sets^51^	Reasoning	✔	✔	✔
Everyday Problem Solving^52^	Reasoning	X	✔	X
Raven’s Matrices^53^	Reasoning	✔	✔	✔
Digit Comparison^54^	Speed of Processing	✔	✔	✔
NIH Toolbox Pattern Comparison^48^	Speed of Processing	X	✔	✔
WAIS-III Digit Symbol^49^	Speed of Processing	✔	✔	✔
CANTAB Delayed Matching to Sample^46^	Working Memory	✔	X	X
CANTAB Spatial Recognition Memory^46^	Working Memory	✔	X	X
CANTAB Spatial Working Memory^46^	Working Memory	✔	✔	✔
NIH Toolbox List Sorting^48^	Working Memory	X	✔	✔
Operation Span Task^55^	Working Memory	✔	✔	✔
WAIS-III Letter Number Sequencing^49^	Working Memory	✔	✔	✔
Controlled Oral Word Associations^56^	Verbal Fluency	✔	✔	✔
ETS Advanced Vocabulary^51^	Vocabulary	✔	✔	✔
NIH Toolbox Oral Reading Recognition Test^48^	Vocabulary	X	✔	✔
NIH Toolbox Picture Vocabulary^48^	Vocabulary	X	✔	✔
Shipley Vocabulary^57^	Vocabulary	✔	X	✔
CANTAB Graded Naming Task^46^	Vocabulary	✔	✔	✔

The purpose of the present article is to provide an overview of this rich dataset. The Park lab and others have published extensively from these data (summarized in the Usage Notes section), however, the publications thus far have not even come close to yielding the many secrets yet to be uncovered in these data that will help us understand the cognitive neuroscience of aging.

## Methods

### Participants

**Fig. 2:**
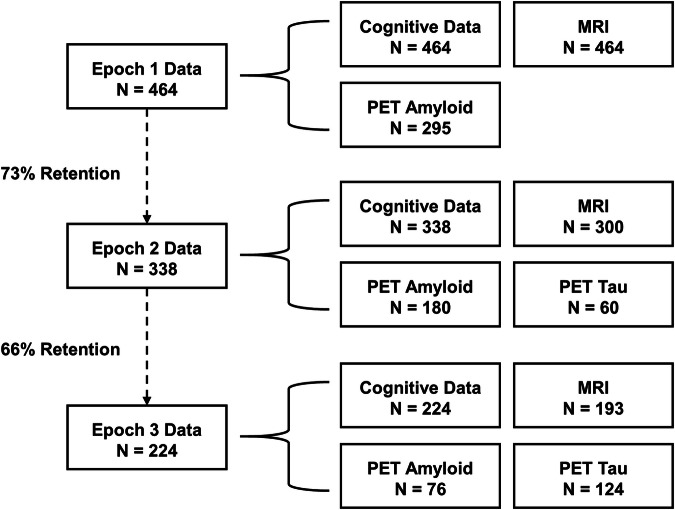
Participant flowchart describing total sample size and quantity of data for key modalities—cognitive data, MRI, PET amyloid, and PET tau—by epoch.

The first epoch of DLBS data collection began in 2008 and included 464 participants ages 21–89. Figure 2 presents the number of initial participants categorized by age group (young, middle-aged, old, and very old), and it also shows the number of participants in each decade of life. The design of the study allows for the treatment of age as a between groups variable with participants grouped by age (e.g., young = 21–39; middle-aged = 40–59; old = 60–79; very old = 80–89, as some past investigations with these data have done^7,8^) or as a continuous variable in analyses (see Figure 1). Basic demographic information regarding participants appears in Table 2. In general, the participants are moderately well-educated and are about 2/3 female. This sex ratio was maintained within each decade of the study. All participants were recruited from the Dallas-Fort Worth area using media advertisements primarily on the radio; ads were broadcast to diverse listeners by subscribing to stations that varied in content and audience. Flyers were also distributed throughout the community. No participants were recruited from colleges or universities, but volunteers who were college students were accepted if they learned about the study through community advertisements. No older adults lived in assisted living or nursing homes. Of these initial 464 participants, 338 returned for epoch 2 (73% retained from epoch 1), and 224 returned for a final epoch 3 (48% retained from the epoch 1 sample).

**Table 2:** Demographic and sample statistics.

	Epoch 1 (N = 464)	Epoch 2 (N = 338)	Epoch 3 (N = 224)
Age, y	58.31 (18.14)	62.45 (17.26)	65.07 (16.64)
Age Range, y	21–89	25–93	31–97
Interval to next epoch, y	3.93 (0.36)	4.92 (0.83)	—
Female/Male, n (% Female)	287/177 (61.9%)	216/121 (64.1%)	137/78 (63.7%)
Baseline Education, y	15.79 (2.29)	—	—
MMSE	28.39 (1.24)	29.04 (1.25)	29.14 (1.25)
APOE e4 carrier	102 (21.98%)	80 (12.67%)	
Caucasian	395 (85.1%)	298 (88.4%)	200 (89.3%)
Black/African-American	27 (5.8%)	16 (4.7%)	5 (2.2%)
Asian American /Pacific Islander	14 (3%)	8 (2.4%)	6 (2.7%)
Native American	5 (1.1%)	4 (1.2%)	4 (1.8%)
Hispanic/Latino	25 (5.4%)	15 (4.5%)	13 (5.8%)

*Note*. Age and the interval between epochs provided here are calculated based on the cognitive battery testing date. Similar metrics for the MRI and PET scanning sessions are available in the data set. Abbreviations: MMSE, Mini-Mental State Examination; *SD*, standard deviation; y, years.

### Procedures

Each epoch of data collection consisted of up to five testing sessions: two cognitive testing sessions, an MRI session, and separate PET scanning for amyloid with ^18^F-AV-45 (florbetapir made by Avid Radiopharmaceuticals, a division of Eli Lilly) and for tau with ^18^F-AV-1451 (flortaucipir). Survey data were collected at the cognitive testing session during epoch 1, but for epochs 2 and 3 participants completed the surveys at home and emailed them to the experimenter. As detailed in the Supplementary Information, sample sizes for epochs 2 and 3 survey data were somewhat reduced due to participant non-response (see header tables under headings “Physical Health”, “Mental Health and AD Screening Data”, and “Psychosocial” sections in the supplementary data). Retention and major imaging modality by epoch is visualized in Fig. 2.

Eligibility criteria were designed to ensure that this was a relatively cognitively healthy sample; participants were disqualified if they indicated they had suffered from alcoholism or drug addiction in the last 5 years, had a major heart attack or cancer that was systemically treated with chemotherapy and/or for major psychiatric and neurological disorders, chronic substance abuse, or had been unconscious for more than 10 minutes at some point in their life. All participants were right-handed and all scored a minimum of 26 on the Mini-Mental State Examination^9^, which is a common cutoff for mild cognitive impairment^10^. The MMSE cutoff was reduced to 22 for epochs two and three to allow for reduced cognitive function, although the lowest observed score was 24. There were three separate IRBs that each required separate consent forms. Participants were allowed 45 minutes for consenting. They signed one for the central study inclusive of all cognitive, behavioral, and MRI-based assessments, as well as separate consent forms for the Amyloid and Tau imaging processes, respectively. Lastly, participants separately consented to have their data retained in an open-access format for future analyses. All imaging took place at the University of Texas at Southwestern Medical Center and they were the IRB of record (IRB #: STU 072010-112; 072010-219; 092015-003r).

At each epoch of data collection, participants were compensated up to $600 for their participation, based on which assessments were completed. This included $75 payments for completion of each of two cognitive assessment sessions, $25 for completion of take-home questionnaires, $100 for completion of the structural/functional MRI scanning section, $25 for additional completion of the hypercapnia fMRI scan (described below), and $300 for full completion of all of these sessions. When PET-Amyloid imaging became available midway through epoch 1, all subsequent participants over age 35 were qualified for the PET-Amyloid portion of the study which included a total of $185 in compensation–a $60 payment for completion of a physician screening visit and a $125 payment for completion of the PET-Amyloid scan itself, at epoch 1 and each subsequent epoch of data collection. When PET-Tau imaging was added to the study during epoch 2 of data collection, participants aged 50 or older were recruited to the PET-Tau portion of the study and received a $25 payment for completion of a screening visit and a $100 payment for completion of the PET-tau scan itself, at epoch 2 and epoch 3 of data collection. If participants experienced claustrophobia, or any event in some way resulted in an incomplete session, the participant was fully compensated for the session.

We developed several procedures to anonymize the data placed on the web. We strove to ensure that another individual could not recognize a friend or relative, but also that an individual participant would be unable to recognize self. Every participant was assigned a unique number which was carried through every task and brain scan. Although we collected relatively complete medical data, these data are not included in the web-based data records to maintain confidentiality. Second, no specific dates for participation are recorded. Rather, the first day of testing is entered as Day 1, and all subsequent dates are entered as the number of days lapsed since Day 1.

#### Cognitive tasks

Participants were tested on cognitive tasks individually in a quiet room. Each cognitive task was representative of one of six cognitive constructs: speed of processing, working memory, episodic memory, reasoning, vocabulary, and verbal fluency. Note that some tasks were discontinued or added as the study progressed (e.g., NIH Toolbox was implemented in epochs 2 and 3); detailed descriptions of each task, as well as data availability, are provided in the “Keys to the Kingdom” (“Cognitive Data Constructs”, sections 1–7). These measures are listed in Table 1 in the preceding section, along with source citations and availability by epoch.

#### Questionnaire data

We collected numerous measures of health, psychosocial status, mental health, and Alzheimer’s Disease screening measures. The “Keys to the Kingdom” has extensive information about these surveys and includes detailed records of participants’ responses (“Health and Psychosocial Data”). The health and psychosocial questionnaires are listed in Table 3, along with source citations and availability by epoch.

**Table 3:** Availability of questionnaire and physical health data by epoch.

Assessment	Physical Health Assessments			
	Construct	E1	E2	E3
Blood Pressure	Physical Health	✔	✔	✔
Fitness Survey^58^	Physical Health	✔	✔	✔
NIH Toolbox Motor Assessment^48^	Physical Health	X	✔	✔
SF-36^59^	Physical Health	✔	✔	✔
**Assessment**	**Mental Health Assessments**			
	**Construct**	**E1**	**E2**	**E3**
Alzheimer’s Disease Assessment Scale-Cognitive Subscale (ADAS-COG)^60^	Mental Health	X	✔	✔
Center for Epidemiological Studies-Depression (CESD)^61^	Mental Health	✔	✔	✔
Geriatric Depression Scale^62^	Mental Health	✔	✔	✔
**Assessment**	**Psychosocial Assessments**			
	**Construct**	**E1**	**E2**	**E3**
Big Five Inventory^63^	Psychosocial	X	✔	✔
Daily Activities Questionnaire^64^	Psychosocial	✔	✔	✔
Lifetime Cognitive Activities^65^	Psychosocial	✔	✔	✔
Martin and Park Environmental Demands Questionnaire^66^	Psychosocial	✔	✔	✔
Metamemory in Adulthood Questionnaire^67^	Psychosocial	X	✔	✔
Need for Cognition Survey^68^	Psychosocial	✔	✔	✔
NIH Toolbox Emotion Measures^48^	Psychosocial	X	✔	✔
Personality Survey^69^	Psychosocial	✔	X	X
Psychological Well-Being^70^	Psychosocial	✔	✔	✔
Revised Neuroticism-Extraversion-Openness Personality Inventory (NEO-PI-R)^71^	Psychosocial	✔	X	X
Satisfaction with Life Scale^72^	Psychosocial	✔	✔	✔
Scale of Positive and Negative Experience^73^	Psychosocial	X	✔	✔
Self-Concept Clarity Survey^66^	Psychosocial	✔	✔	✔

#### MRI Data acquisition

**Fig. 3:**
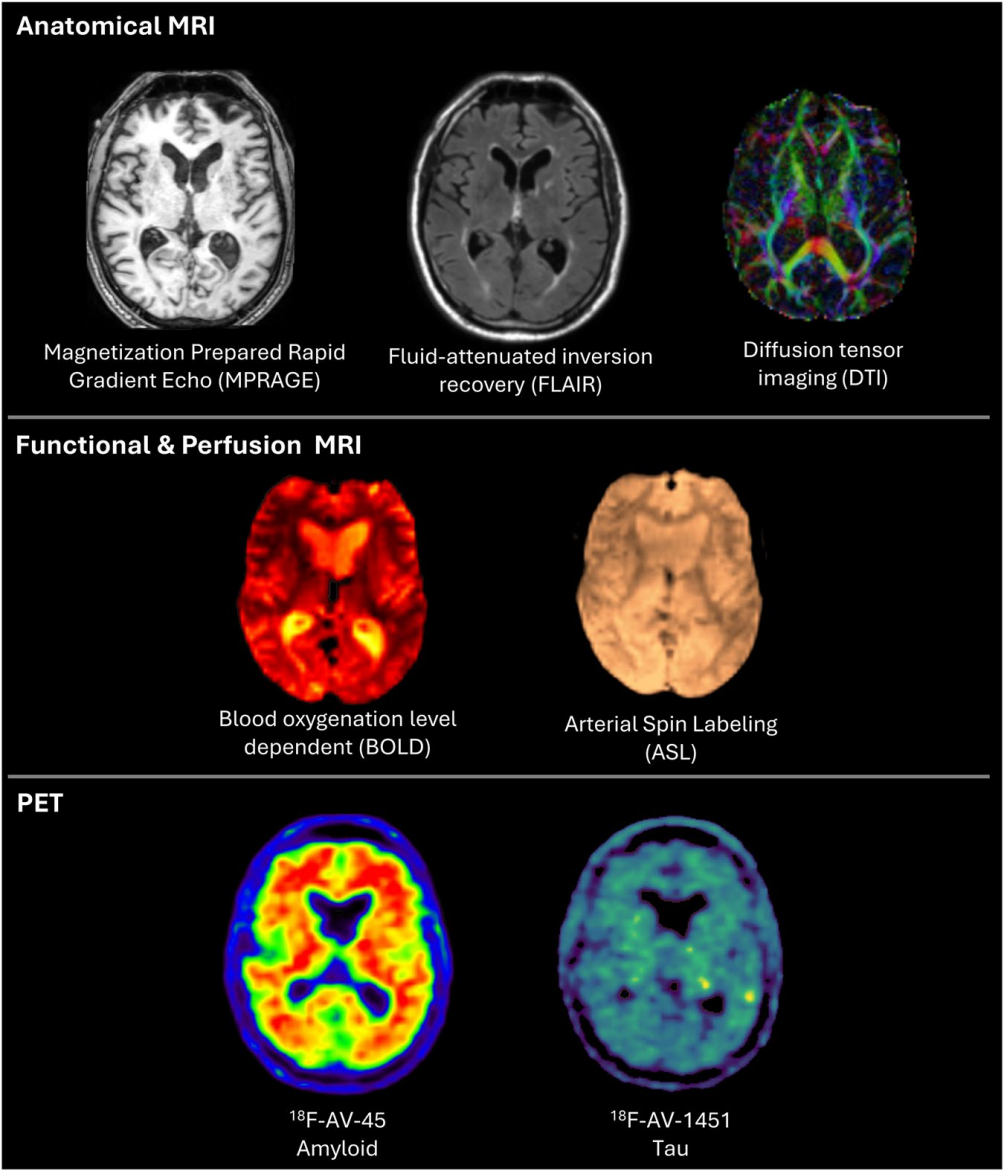
Imaging modalities available in the DLBS dataset.

Participants contributed T1-weighted images, functional MRI images, arterial spin labeling (ASL) images and diffusion tensor imaging (DTI) images at each epoch of assessment. All methodologies were administered during the MRI session of each epoch, and utilized the same scanners and 8-channel headcoil as specified below. Examples of brain imaging modalities available in the DLBS dataset are visualized in Fig. 3.

##### T1-weighted anatomical images

All participants for epochs 1 and 2 were scanned on a single 3 T Philips Achieva scanner (Philips Medical Systems, Best, The Netherlands) with an 8-channel head coil at the University of Texas Southwestern Medical Center (UTSW). At epoch 3, a newer 3 T Achieva scanner at UTSW was used with the same imaging parameters, though the original 8-channel head coil was retained for use with the new scanner, for consistency. Anatomical data were collected using a T1-weighted MP-RAGE sequence with the following parameters: 160 sagittal slices, 1 × 1 × 1 mm^3^ voxels, 204 × 256 × 160 matrix, TR = 8.1 ms, TE = 3.7 ms, shot interval = 2100 ms, inversion time = 1100 ms, flip-angle = 12°.

##### Functional MRI images

Functional MRI data were acquired using a T2*-weighted echo-planar imaging sequence with full brain coverage and the following parameters: 43 interleaved axial slices per volume parallel to the AC-PC line, SENSE = 2, 3.4 × 3.4 × 3.5 mm voxels, 64 × 64 × 43 matrix, FOV = 220 × 220 mm, TR = 2 s, TE = 25 ms, flip angle = 80°. Scanner par/rec files were converted to Neuroimaging Informatics Technology Initiative (NIFTI) format using *BIDSCOIN* (Donders Institute, Nijmegen, The Netherlands).

##### Arterial spin labeling images

Cerebral blood flow (CBF) was measured via a pseudo-continuous arterial spin labeling sequence described by Thomas *et al*.^11^. Scan parameters were: pseudo-continuous ASL with a duration of 1.65 seconds, delay = 1.525 seconds, labeling radio frequency duration = 0.5 ms, pause between radio frequency pulses = 0.5 ms, labeling pulse flip angle = 181, single-shot echo planar imaging, field-of-view = 240*240 mm^2^, matrix = 80*80, 27 axial slices, thickness = 5 mm, repetition time/echo time = 4,020 ms/14 ms, 30 pairs of label and control images. Total scan duration was 4 minutes.

##### Diffusion-weighted images

DTI scans were performed using a single-shot spin-echo echo planar imaging sequence with the following parameters: scan duration = 217.2 sec; repetition time = 4410 ms; echo time = 51 ms; flip angle = 90◦; scan resolution = 112 × 110; field of view = 224 mm × 149 mm × 224 mm; number of slices = 50; slice thickness = 2 mm; voxel size = 1.75 mm × 1.75 mm × 2 mm. The diffusion-sensitizing gradients were applied along 30 non-collinear directions with a b value of 1000 s/mm2, and one volume was acquired without diffusion weighting (b = 0).

#### MRI Data procedures

##### Structural images

As documented in the Data Records section below, the MRI and fMRI images included in this dataset are raw scanner images converted to NIFTI format without additional preprocessing. These images received no further processing so interested researchers can instead implement their preferred MRI processing pipelines. However, structural metrics such as grey matter volume and thickness are also provided (estimated from T1-weighted imaging) that were developed using the cross-sectional FreeSurfer ver. 5.3 pipeline. Extensively trained lab personnel inspected the FreeSurfer-constructed white and gray matter surfaces and made manual edits, when necessary. These edited volumes were then independently reviewed by members of Dr. Gagan Wig’s lab to ensure accurate anatomical segmentations were achieved (for additional details of manual editing procedures see Savalia *et al*.^12^).

##### fMRI Tasks

**Fig. 4:**
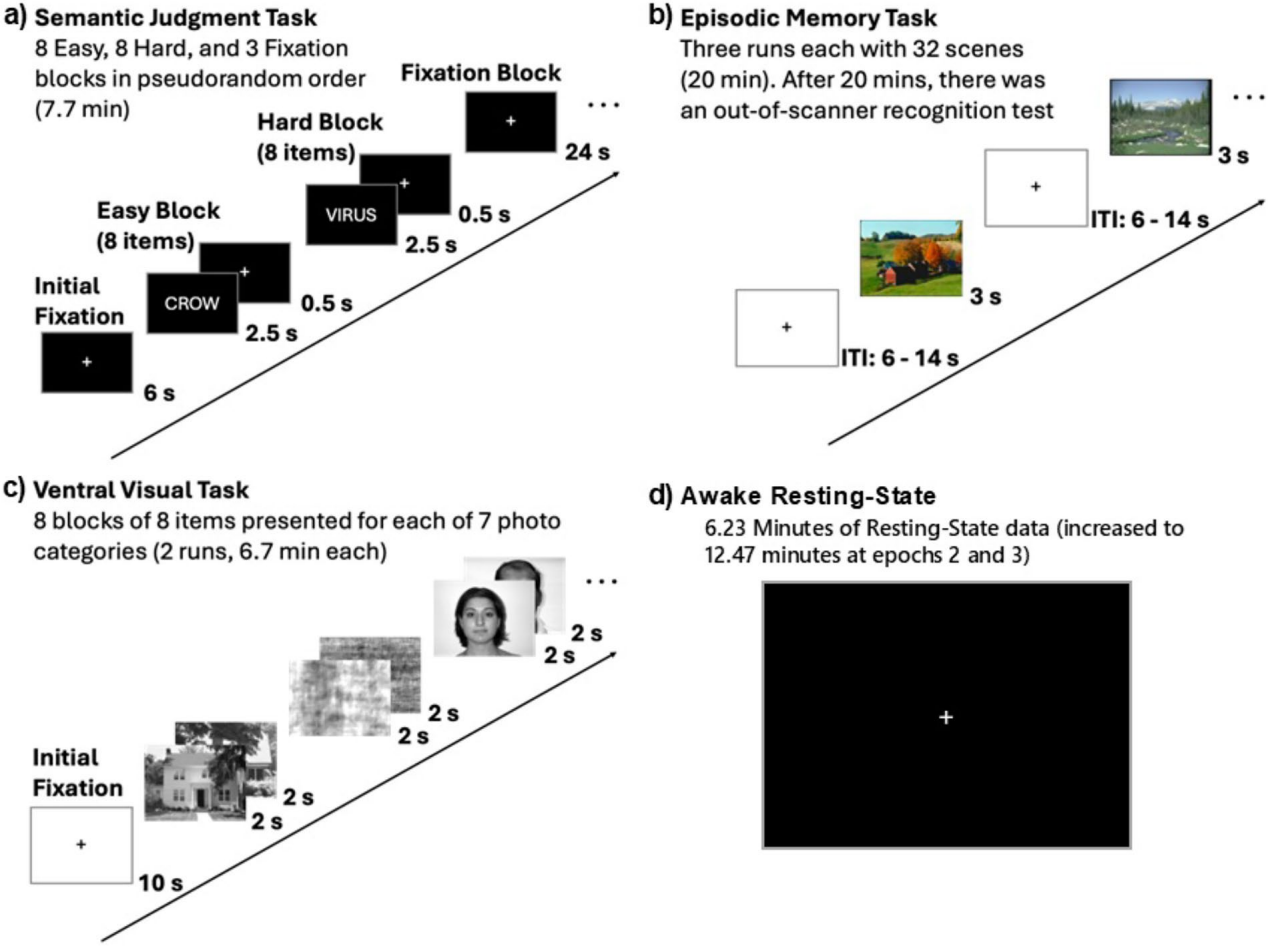
Trial schematic four in-scanner tasks: (**a**) Semantic Judgment Task, (**b**) Episodic Memory Task, (**c**) Ventral Visual Task, and (**d**) Awake Resting-state (static fixation cross).

Four in-scanner cognitive tasks were administered to participants during fMRI acquisition at each epoch of data collection. These tasks – the Subsequent Memory Task, Semantic Judgement Task, Face/Place Passive Viewing Task, and awake resting-state – are described below, and are additionally detailed in primary publications cited in each task’s respective description. These functional scan tasks are visually summarized in Fig. 4. We note that the subsequent memory tasks was designed to focus on hippocampal and frontal regions; the semantic judgement task focused on frontal parietal regions, and the passive viewing category task was directed primarily towards ventral/visual areas. In addition, all participants also had the opportunity to undergo hypercapnia manipulation at their option to assess cerebrovascular reactivity at each epoch, which also utilized the fMRI scan sequence detailed above.

##### fMRI subsequent memory task

This task was designed to assess how fMRI BOLD signal during the incidental encoding of images relates to performance in a subsequent recognition test^13,14^. Using an incidental encoding paradigm, participants were presented with outdoor landscape scenes and had to determine whether there was water present in each scene by pressing a “yes” or “no” button. The task consisted of three runs with a random ordering of 96 outdoor scenes (32 scenes per run) presented in an event-related design. Each image was presented for 3 s, and intertrial intervals (ITIs) were jittered between 4 and 14 seconds. The total scan time was 20 minutes. Responses were recorded using a fiber-optic button box held in the right hand.

Visual stimuli were presented using E-prime software^15^, projected through the back of the scanner and viewed though a mirror attached to the head coil. Approximately twenty minutes after the encoding trial, an off-line recognition task was administered outside the scanner. A total of 192 pictures were presented, consisting of 96 previously encountered target scenes and 96 closely matched lure scenes. Participants were instructed to make one of three judgements: 1. “high confidence remember” to indicate confident recognition of the exact picture; 2. “low confidence remember” to indicate recognition with low confidence; 3. “new item” to indicate that the picture was not previously presented. The recognition task allowed participants to respond at their own pace with a maximum response time of 4 seconds for each trial.

##### fMRI semantic judgment task

**Fig. 5:**
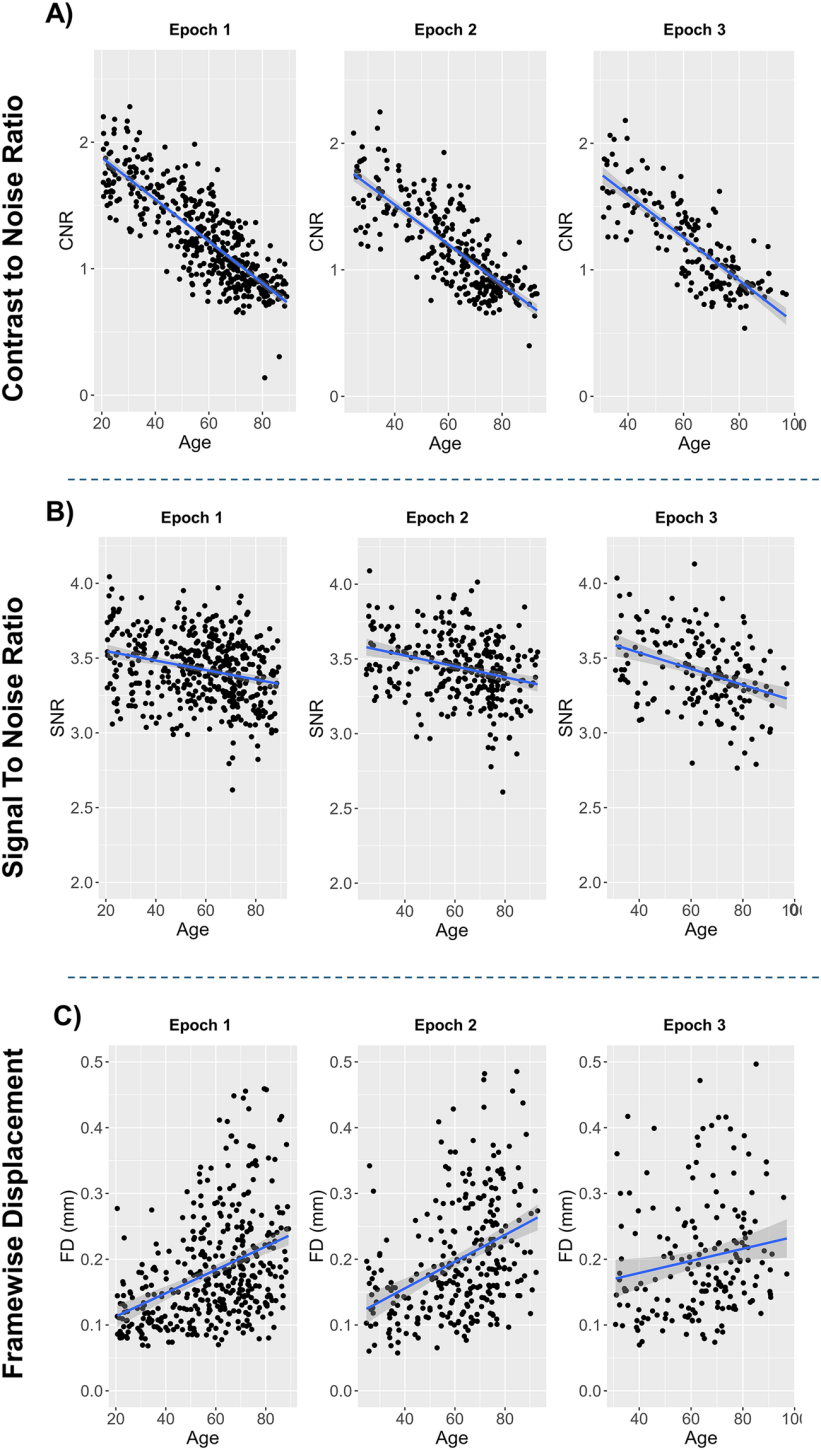
Estimates of image quality produced by MRIQC^29^ across epochs of data collection: (**A**) contrast-to-noise ratios for T1-weighted images, (**B**) signal-to-noise ratios for the semantic judgment (“Words”) fMRI task, and (**C**) framewise displacement for the resting-state task. Abbreviations: CNR, contrast-to-noise ratio; FD, framewise displacement; SNR, signal-to-noise ratio.

This block-design fMRI task was designed to investigate patterns of fMRI BOLD signal during semantic judgements on words^13,16^, with two levels of difficulty (Fig. 5B)^16^: Items were drawn from a word bank that included 64 ambiguous and 64 unambiguous words. Participants were presented with 8 blocks of easy, unambiguous word judgments and 8 blocks of hard, ambiguous word judgment, with 8 words per block, Examples of easy (unambiguous) items included “walrus”, “truck”, “asphalt”. Examples of hard (ambiguous) words included “speaker”, “virus”, and “sponge”. Upon viewing each word, participants made a yes or no judgment by pressing a button with their right index for “yes” (living) or middle finger for “no” (not living)The easy/unambiguous and hard/ambiguous blocks were presented in a pseudo random order.

Visual stimuli were using E-prime software^15^ projected through the back of the scanner and viewed through a mirror attached to the head coil. Each word was displayed for 2500 ms followed by a 500 ms fixation (crosshair). During each stimulus presentation, the reaction time to make a button press was recorded. Additionally, three 24-second fixation blocks were used as a baseline. The total scan time was 7.7 min.

*Note:* A study-wide stimulus onset asynchrony file is provided in this project’s root directory (task-Words_run-1_events.tsv).

##### fMRI face/place passive viewing task

This task was designed to assess age-related differences in the neural selectivity to various visual stimuli^17^. Participants viewed images from seven categories: human faces, primate faces, domestic cat faces, wild cat faces, houses, chairs, and phase-scrambled control stimuli. Each category consisted of 64 gray-scale photographs (400 pixels wide × 300 pixels tall), except for domestic and wild cat faces, which had 32 photographs each. The images of human faces were sourced from the face library of Minear and Park^18^, and included individuals of different ages, races, and genders. Animal photographs (primate, domestic and wild cats) were obtained from the internet and cropped to ensure clear visibility of the animal’s face. Similar to human faces, only animal images with front-facing views and neutral expressions were selected. Houses were photographed from Ann Arbor, MI and Toledo, OH, and photographs of chairs were sourced from furniture websites. To create control stimuli, phase-scrambled images were generated by combining and then scrambling the phase information in all of the experimental stimuli. This process preserved the spatial frequency information while rendering the visual content meaningless.

The images were presented in 16-second blocks, with each image displayed for 2 seconds. A total of 8 blocks were presented for each category, distributed across two separate runs with four blocks per run. The order of the blocks was randomized for each participant to minimize potential order effects. Participants were instructed to view each picture while receiving no instructions about the types of processing in which they should engage (passive viewing task). The stimuli were presented using E-prime software^15^, and displayed through a back-projection system.

##### fMRI awake “resting-state”

In each epoch, participants completed awake resting-state scans, which were meant to allow extraction of “intrinsic” patterns of functional connectivity^19–22^. During this time participants were instructed to remain awake and relax while fixating on a black crosshair against a white background. At the end of the run, the experimenter verified that participants complied with the instructions and did not fall asleep during the functional scan via verbal confirmation. One run of 154 BOLD acquisition was collected in epoch 1. Two runs of resting-state data were collected in epochs 2 and 3. The duration of the run was increased from 154 BOLD volumes to 180 volumes part-way through epoch 2.

##### fMRI hypercapnia

Cerebrovascular reactivity (CVR) was assessed at each epoch via a hypercapnia block-design model^23^. Participants inhaled a 5% CO_2_ gas mixture (5% CO_2_, 74% N_2_, and 21% O_2_) while BOLD MR images were simultaneously captured. During the CVR scan, participants wore a nose clip and alternated between breathing room air and the prepared gas (60 seconds CO_2_, followed by 60 seconds of room air, repeated three times) through a mouthpiece. Additionally, the concentration of CO_2_ in the lungs (end-tidal CO_2_ or EtCO2), which closely reflects arterial CO_2_ levels, was recorded throughout the breathing task using a capnograph. The total duration of the CVR scan was 7 minutes.

#### PET data acquisition and analysis

##### Pet amyloid data acquisition

For PET amyloid scanning, participants were injected with an approximately 370 MBq (10 mCi) bolus of ^18^F-AV-45 (^18^F-florbetapir; Avid Radiopharmaceutical/Eli Lilly). There was a waiting period of approximately 30 minutes prior to positioning for scanning in a Siemens ECAT HR PET scanner (Siemens, Munich, Germany) at UTSW for epochs 1 and 2. For epoch 3, a small subset of participants was scanned on a new Siemens Biograph mCT Flow Edge scanner. Scanning started with a 2-minute scout to ensure full brain coverage and lack of planar rotation. At 50 minutes after injection, PET data acquisition began, using a dynamic emission acquisition sequence including two five-minute frames. Immediately after, an internal rod source transmissions scan was performed for 7 minutes. This image was reconstructed using back-projection with a 6 mm full width half maximum (FWHM) Gaussian filter with 4 iterations, 16 subsets (21 subsets at epoch three), and a 3 mm FWHM ramp filter. A total of 295 participants were scanned at epoch 1, 180 participants at epoch 2 and 76 participants at epoch 3.

##### PET tau data acquisition

For PET tau scanning, participants were injected with an approximately 370 MBq (10 mCi) bolus of ^18^F-AV-1451 (^18^F-flortaucipir; Avid Radiopharmaceutical/Eli Lilly) 80 minutes prior to scanning in a Siemens ECAT HR + PET/CT scanner (Siemens, Munich, Germany) at UTSW for epoch 2; PET tau data were not collected at epoch 1 as flortaucipir was not yet available for general use. With the cooperation of Avid Pharmaceuticals, a team at UTSW led by XianKai Sun synthesized AV1451 with the UTSW cyclotron. As with PET amyloid scanning, for Epoch 3 a Siemens Biograph mCT Flow Edge scanner was used. The PET scan duration was 20 minutes and reconstruction used 3 mm FWHM smoothing with 4 iterations and 21 subsets (3 iterations and 34 subsets at Epoch 3).

##### PET data processing

In addition to the raw PET amyloid and PET tau images described above, we also provided pre-calculated regional standardized uptake ratios (SUVRs) for both imaging modalities (see Data Records). To obtain PET amyloid SUVRs, first, the second PET run was registered to the first run in the PET sequence and then the two runs were averaged. Second, for each subject with at least two epochs of data, a mean anatomical template was created using Freesurfer 5.3’s *mri_robust_template* procedure. For participants with only one epoch of data, their T1-weighted image collected at epoch 1 was used in place of this mean anatomical template. Third, the PET data were registered to this mean template (or T1-weighted image, as noted above.) and the relevant PET counts were extracted from volumes of interest from the Desikan-Killiany atlas^24^. Finally, SUVRs were estimated using a whole-cerebellum reference. Global SUVR, an estimate of cortical amyloid, was taken as the average SUVR across eight regions covering most of cortex except the somatosensory and motor strips:. Covered regions included anterior cingulate, posterior cingulate, lateral prefrontal, orbitofrontal, precuneus, lateral parietal, lateral occipital, and lateral temporal cortices.

For PET tau SUVR calculation, a similar processing pipeline was used, except that PET data were registered to each participant’s T1-weighted image from the same epoch, rather than a mean anatomical image. The corresponding data set includes SUVRs for a temporal meta region presented by Jack *et al*.^25^, which includes inferior temporal, middle temporal, entorhinal, parahippocampal gyrus, fusiform, and amygdala. This region was selected due to its sensitivity in detecting tau accumulation in otherwise healthy aging.

*Note:* The PET data were processed cross-sectional or calculation of amyloid and Tau SUVR values due to a change in the specific PET scanner utilized between epoch 2 and epoch 3. This approach was utilized to avoid adding measurement noise by comparing measures between these two scanners.

#### Genotyping

DNA isolation and genotyping were performed for APOE, BDNF, COMT, and DRD2. Venous blood samples were collected into EDTA-anti-coagulated tubes and genomic DNA was isolated by standard protocols^26^. Fragments containing each of the polymorphisms were amplified from genomic DNA by polymerase chain reaction (PCR) using Taq DNA polymerase (Roche Diagnostics; Indianapolis, IN) and a thermal profile, reaction conditions and primer sequences optimized for each polymorphism. All amplifications were carried out in an ABI 7900HT thermal cycler (Applied Biosystems, Inc; Foster City, CA). Genotypes were determined by a number of methods, depending upon the nature of the polymorphism. For single nucleotide polymorphisms (SNPs; ApoE, BDNF, COMT, DRD2) genotypes were determined by real-time PCR using TaqMan probes unique for each SNP (Applied Biosystems, Inc; Foster City, CA). Assay ID’s utilized for each single nucleotide polymorphism are listed in Supplementary Table 1, and further details of this procedure can be found in the supplementary material (see “Construct 19: Genotyping”).

## Data Records

All data for the DLBS are available open access on OpenNeuro.org (10.18112/openneuro.ds004856.v1.2.0)^27^. Information that could be used to identify participants (e.g., birthdates, addresses), as specified by HIPAA, have been removed from all records. Each participant was assigned an arbitrary participant identification number that is consistent across all data types that ranges from 12–4876. Anatomical T1 and T2* scans have been defaced primarily using *PyDeface* ver. 2.0.2 (The Poldrack Lab, CA, USA) with a small subset of faces defaced using *mri deface*^28^. A README file and our data dictionary “Keys to the Kingdom” (DLBS KTTK.pdf), both located in the root directory, provide detailed descriptions of the project and data set, including detailed task descriptions, variable descriptions, and sample sizes for key data types. A printed copy of “The Keys to the Kingdom” is available by request from (DLBS@utdallas.edu).

Raw imaging data are organized according to the Brain Imaging Data Structure (BIDS, ver. 1.8, 2022) standard using *BIDSCOIN* with participant-specific directories <sub-[insert ID here] >, which include up to three subdirectories based on data collection epoch (e.g., <ses-wave1>) and further subdirectories separated by imaging modality. Imaging modalities included: anatomical scans (‘anat’ folder), diffusion weighted images (‘dwi’), functional MRI scans (‘func’), perfusion imaging (‘perf’), and PET imaging (‘pet’). Stimulus onset asynchrony values are provided in the *_events.tsv and *_events.json files located in the root directory and in each subject’s ‘func’ folder. Demographic information and subject-specific testing intervals are provided in the participants.tsv and participants.json files in the root directory. Pre-processed cognitive data, survey data, and genotyping data, as well as summary structural (FreeSurfer metrics), PET amyloid (^18^F-AV-45) SUVRs, and PET tau (^18^F-AV-1451) SUVRs for each participant, are in the ‘phenotype’ directory, each in the form of Microsoft Excel spreadsheets with tabs for each epoch of data collection.

## Technical Validation

### Behavioral and questionnaire data

The accuracy of the behavioral and questionnaire data was rigorously evaluated. After completion of data collection, the experimenter entered the data via a computer download with a small amount of data entered by hand. Immediately thereafter, a second experimenter did a check on the download process and the data entered by hand. The next step was to transfer the data to the “Keys to the Kingdom”. This step did not begin until all the data were collected. Two teams of individuals from the lab were assigned to the behavioral data. One team entered or moved the data into the “KTTK”, and the second team reviewed every single number. Thus this process required that the data was checked four times before it was deemed accurate.

### Selective attrition

To determine if there were baseline differences between participants who attended all three epochs of data collection versus those who contributed to only one or two epochs, between-subjects ANOVAs were conducted with number of epochs completed as the grouping variable (Table 4). The number of epochs completed was determined based on whether participants had attended the cognitive testing session for each epoch. A Goodness of Fit Chi-square analysis was also conducted to determine if sex distribution differed as a function of the number of epochs completed. The main effect of number of epochs completed was not statistically significant for baseline age, *F*(2, 463) = 0.98, *p* = 0.377, *η*^*2*^ = 0.004, MMSE scores, *F*(2, 462) = 2.27, *p* = 0.105, *η*^*2*^ = .01, or sex distribution, χ^2^(2) = 2.14, *p* = 0.343. However, there was a significant effect of number of epochs completed on education, *F*(2, 462) = 3.61, *p* = .028, *η*^*2*^ = 0.02, as those who attended more epochs of data collection had slightly more years of education (Table 3). Baseline cortical amyloid SUVR was also examined in subsamples who completed 1 (n = 111), 2 (n = 112), or 3 (n = 72) epochs of PET amyloid scanning, in order to assess whether presence of AD-related brain pathology during an initial visit relates to likelihood of subsequent return. The between-subjects ANOVA indicated that baseline SUVR did not significantly differ as a function of number of PET amyloid scans completed, *F*(2, 292) = 2.38, *p* = .095, *η*^*2*^ = .02, and that SUVRs were relatively similar for those with 1 (*M* = 1.09, *SD = *0.15), 2 (*M* = 1.09, *SD = *0.16), or 3 (*M* = 1.05, *SD = *0.07) epochs of PET amyloid data.

**Table 4:** Selective-attrition analysis.

	Total Epochs Attended			
	1 (N = 119)	2 (N = 138)	3 (N = 207)	*p*
	Mean (*SD*)	Mean (*SD*)	Mean (*SD*)	
Baseline Age, y	59.11 (19.69)	59.57 (18.86)	57.01 (16.66)	0.377
Female/Male, n (% Female)	67/52 (56.3%)	87/51 (63.0%)	133/74 (64.3%)	0.343
Baseline Education, y	15.45 (2.32)	15.61 (2.48)	16.09 (2.12)	0.028
MMSE	28.27 (1.24)	28.29 (1.28)	28.53 (1.20)	0.105

*Note*. Age here was calculated based on the cognitive battery date. Abbreviations: MMSE, Mini-Mental State Examination; *SD*, standard deviation; y, years.

### Structural neuroimaging data

We assessed the quality of T1-weighted structural MRIs across epochs using *MRIQC*^29^ for 179 participants with all three epochs of data. For these T1-weighted images, the contrast-noise-ratio (CNR), an index describing the clarity of separation between grey and white matter, is provided in Fig. 3a. For CNR, we investigated whether there were systemic differences in image quality across epochs of data collection using a repeated measures ANOVA controlled for age and sex with the image metric as the variable of interest. Estimated CNR significantly differed as a function of epoch, *F*(2, 352) = 8.49, *p* < 0.001, *η*^*2*^ = 0.05, with CNR decreasing slightly across epochs. Bonferroni-adjusted post hoc pairwise comparisons revealed that this reduction in signal quality was numerically larger going from Epoch 1 to 2 (*M*_difference_ = 0.14, *SD*_*difference*_ = 0.13, *p* < 0.001), than from Epoch 1 to 3 (*M*_difference_ = 0.03, *SD*_*difference*_ = 0.15, *p* = 0.015), though both were statistically significant.

In addition to raw T1-weighted structural images, we also provide FreeSurfer structural volume estimates for each participant at each epoch (see Data Records). Manual quality check and editing of all FreeSurfer parcellations and segmentations were conducted by trained experimenters to ensure the accuracy of these estimates. All experimenters who conducted manual review and editing on T1-weighted structural images received extensive training on a pre-specified set of procedures developed by Dr. Wig’s laboratory (see Savalia *et al*., 2017^12^ for methodological reference). Completed manual edits were submitted to another highly experienced individual for independent review. If the resulting surface boundaries (i.e., pial, white surfaces) did not pass quality control by this individual, it was returned for further manual editing. This procedure ensured that all structural surface boundaries were of relatively equivalent quality, using the same high standard for all.

### Functional neuroimaging data

Because MRIQC’s computations were very time-intensive when conducted for this relatively large data set, MRIQC was only performed on the simpler (one-run) Words task using 176 participants with complete data across the three epochs. This was meant to serve as an illustrative example of fMRI scan quality across the three epochs. Signal-to-noise ratio was used as an index of image quality.

Finally, framewise displacement (FD) was used as an index of head motion, and by extension image quality, for resting-state images. After controlling for age and sex, there was a significant main effect of epoch on SNR, *F*(2, 346) = 3.52, *p* = 0.031, *η*^*2*^ = 0.02. Bonferroni-adjusted post hoc pairwise comparisons revealed that SNR did not significantly change from Epochs 1 to 2 (*M*_difference_ = 0.02, *SD*_*difference*_ = 0.12, *p* = 0.196), but there was a small significant reduction in SNR from epochs 2 to 3 (*M*_difference_ = 0.05, *SD*_*difference*_ = 0.15, *p* < 0.001; Fig. 5B). In contrast, and as expected, after controlling for age and sex, epoch did not have a significant main effect on FD, *F*(2, 346) = 0.10, *p* = 0.907, *η*^*2*^ = 0.001 (Fig. 5C). This suggests that head motion was relatively similar across the three epochs.

## Usage Notes

### Prior DLBS publications

Aspects of the DLBS have already been explored in extensive prior publications using this dataset. These prior publications are briefly summarized here, and we encourage their use as methodological and theoretical references for use with the DLBS database. The DLBS database has most notably been utilized to examine age-related differences in task-positive functional recruitment and compensation, exemplified in studies by H. Park *et al*.^30^, Kennedy *et al*.^13^, Chen *et al*.^31^, and Hennessee *et al*.^7^. A subset of this work has examined age-related differences in neural selectivity in response to visual stimuli^32,33^. The resting-state functional data have been applied to examine the trajectory of alterations in functional connectivity, large-scale brain network organization, and areal parcellation over the lifespan in detail, in work from Wig and colleagues^19^–^22^,^34^. The structural MRI data from these same participants has been examined in relation to variance in rate of cognitive decline^8^. Alzheimer’s pathology has also been a primary focus of prior work with the DLBS database, with numerous studies using the database having illuminated the relationship of Amyloid-B and both cognitive^35–38^ and structural^35,39^ decline in later life. Psychosocial factors that have been examined thus far from this dataset include the relationship between self-concept clarity and mental health^40^, the protective effect of business on cognitive function^41^, and the relationship of metamemory to memory performance across ages^16^. Finally, the cerebrovascular reactivity manipulation has been used to examine cerebrovascular aging in detail in a series of studies by Liu and colleagues^23,42,43^.

### Unexplored aspects of the DLBS database

Despite the body of literature that has investigated aspects of the database presented in the present article, numerous aspects of this database have not yet been examined and therefore reflect an opportunity for novel investigations. Most notably, the PET Tau data included in this database have not been scientifically examined, and there is limited work with the DTI. These measures alone offer myriad avenues into understanding Tau-mediated Alzheimer’s pathology as well as both normative and pathological structural change with age, especially when used in conjunction with the other measures included within this database. The genetic data have also been understudies a handful of above-mentioned studies examining effects of the APOE e4 gene^7,36^ on cognitive aging, and other genetic information (i.e. COMT, BDNF) thus far unexplored. Variables related to physical fitness (i.e. NIH Motor Toolbox, SF-36), mental health (i.e. the Geriatric Depression Scale), and personality and other psychosocial factors (i.e. Big 5 Index, SPANE), have similarly been only partly leveraged^44^ in scientific inquiry as of the time of this article’s publication. The authors strongly encourage use of these aspects of the database to facilitate novel scientific inquiry.

It should also be noted that there may be significant cross-cultural differences in the brain particularly with age. For example, this study parallels the Chinese Color Nest Project, a project which is currently in a data collection phase^45^. A great deal can be learned from DLBS in isolation, but combining DLBS with other adult lifespan datasets offers valuable opportunities to understand brain and cognitive aging in different contexts.

## Supplementary information

Dallas Lifespan Brain Study - Keys to the Kingdom

## Data Availability

Code used to process the PET amyloid and tau data are provided on the OpenNeuro.org project site (10.18112/openneuro.ds004856.v1.2.0)^27^. in the ‘/code’ directory. Procedures for manual editing of FreeSurfer output of T1-weighted images are provided at: https://zenodo.org/records/7800191.
