# Supplementary material for: The Dallas Lifespan Brain Study: A Comprehensive Adult Lifespan Data Set of Brain and Cognitive Aging
Source: Sci Data. 2025 May 26;12:846. doi: 10.1038/s41597-025-04847-7 (PMC12106762; doi:10.1038/s41597-025-04847-7)
Supplement: Supplementary file 1 — Dallas Lifespan Brain Study - Keys to the Kingdom [file 41597_2025_4847_MOESM1_ESM.pdf]

CENTER FOR VITAL LONGEVITY

Park Aging Mind Laboratory
Innovation. Intellect. Integrity

THE DALLAS LIFESPAN BRAIN STUDY

Keys to the Kingdom

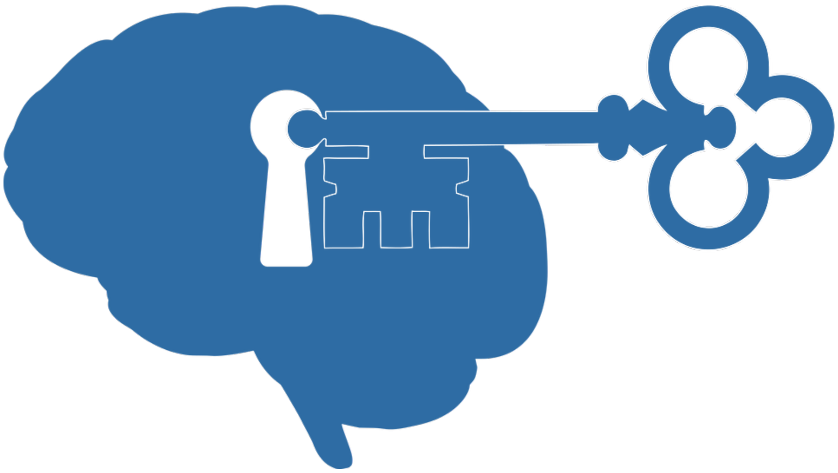

Revised: 2025-06-18

The Dallas Lifespan Brain Study Investigators

Principal Investigator:

- Denise C. Park, PhD

Coinvestigators

- Micaela Chan, PhD
- Marianna Dakanali, PhD
- Kristen Kennedy, PhD
- Hanzhang Lu, MD
- Will Moore, MD
- Karen Rodrigue, PhD
- Neil Rofsky, MD
- Xiankai Sun, PhD
- Carol Tamminga, MD
- Gagan Wig, PhD

Research Scientists

- Gérard Bischof, PhD
- Joseph Hennessee, PhD
- Peiying Liu, PhD

Postdoctoral Fellows

- Sara Festini, PhD
- Ian McDonough, PhD
- Katie Munro, PhD
- Sara Pixley, PhD
- Jenny Rieck, PhD
- Evan T. Smith, PhD

Graduate Students

- Xi Chen, PhD
- Michelle Farrell, PhD
- Ekarin Pongpipat, PhD

Research Staff

- Alexandra Collyer
- Aaron Dotson, MD
- Patrick Evans
- Victor Faner
- Blair Flicker, PhD
- Kimberly Flicker
- Sarah Frank
- Jacqueline Gauer
- Eliza Hearst
- Andrew Hebrank
- Yu (Cindy) Hong
- Carson Katen
- Mandeep Kaur
- Matthew Martin
- Alison Parker
- Kristin Smart

Sponsors

The investigators would like to thank the National Institute on Aging for support of this project (5R37AG-006265-27, RC1AG036199). In particular, we are indebted to Molly Wagster, NIA program officer, for her unflagging encouragement and help throughout the entire project. We also thank Dr. Jonathan King of NIA who kept track of the project and was an additional source of wise counsel during the 12-15 years this project took place. We are grateful as well to Avid Radiopharmaceuticals (a division of Eli Lilly) for providing the project with radiotracers. And most of all, we thank the hundreds of human participants who returned one time after another.

What are the "Keys to the Kingdom"

The "Keys to the Kingdom" is a master document and data dictionary that includes all the components of the Dallas Lifespan Brain Study. Within this document are listed the types of data that were collected, the source documents for each, how to access the study's source documents and spreadsheets, and the coded item names and their abbreviations for each variable found in the spreadsheets.

What is the Dallas Lifespan Brain Study?

The Dallas Lifespan Brain Study (DLBS) is a longitudinal study to examine changes in human cognition as well as changes in brain structure and function across the lifespan. This study represents the first systematic investigation of changes in neural activation across the lifespan, including middle-age. The present work is particularly noteworthy in that we have integrated structural measures of the brain with functional activation patterns to predict both cognitive function and neural activation for encoding tasks. Few, if any, studies have combined both types of neural measures; the standard paradigm in the aging literature has been to use behavioral differences to predict brain function, rather than the reverse, as we have done.

The study was designed to test the same set of participants approximately every 4 years with the following measures: 2 days of cognitive behavioral testing, take-home questionnaires, and an MRI scan session. 464 people participated in Epoch 1, which was collected between 2008-2014. Approximately 4 years later, between 2012-2017, 338 participants (73%) came back for Epoch 2 repeated testing. Finally, approximately 4 years later, between 2018-2022, 224 participants (48%) came back for Epoch 3 data collection. The Epoch 3 intervals are somewhat less standardized due to interference from COVID-19 restrictions.

Types of Data Collected

i. Cognitive Data Constructs
   - Speed of processing
   - Working memory
   - Long term (episodic memory)
   - Reasoning
   - Vocabulary
   - Verbal Fluency

ii. Structural MRI Data

iii. Amyloid PET imaging (AV-45), TAU PET imaging (AV-1451), Genotyping

iv. Functional MRI

v. Health and Psychosocial Data
   - Physical Health
   - Mental Health and AD Screening Data
   - Psychosocial

Accessing DLBS Data

DLBS Datasheets How-To

The Dallas Lifespan Brain Study is hosted publicly on OpenNeuro at https://openneuro.org/datasets/ds004856. Guides for new users to OpenNeuro, including instructions on how to download all or partial databases from OpenNeuro, can be found here: https://docs.openneuro.org/user_guide.html

The OpenNeuro repository is organized in the Brain Imaging Data Structure (BIDS, ver. 1.8, 2022) standard using *BIDSCOIN* with participant-specific directories <sub-XXX>, which include up to three subdirectories based on data collection Epoch (e.g., <ses-wave1>) and further subdirectories separated by imaging modality.

Imaging modalities included: anatomical scans (‘anat’ folder), diffusion weighted images (‘dwi’), functional MRI scans (‘func’), perfusion imaging (‘perf’), and pet imaging (‘pet’). Stimulus onset asynchrony values are provided in the *_events.tsv and *_events.json files located in the root directory and in each subject’s ‘func’ folder.

Demographic information and subject-specific testing intervals are provided in the participants.tsv and participants.json files in the root directory. Pre-processed cognitive data, survey data, and genotyping data, as well as summary structural (FreeSurfer metrics), PET amyloid (^{18}F-AV-45) SUVRs, and PET tau (^{18}F-AV-1451) SUVRs for each participant, are in the ‘phenotype’ directory, each in the form of Microsoft Excel spreadsheets with tabs for each epoch of data collection.

Each of these domains of pre-processed data are summarized in the subsequent chapters, and data dictionaries for the available data are provided where appropriate.

● ***Note:*** We refer to each instance of longitudinal observation as “Epoch” in our published materials for this database, including this document, while labels and abbreviations in our database and data dictionary utilize “Wave” or “W” to refer to each instance of longitudinal observation. “Epoch” and “Wave”/“W” are equivalent terms wherever they appear in this document.

Cognitive Data Constructs

The Dallas Lifespan Brain Study

Revised: 2025-06-18

Using the cognitive data

The cognitive data includes all task information, data coding, and data spreadsheets for each of the cognitive constructs in the Dallas Lifespan Brain Study.

The cognitive data in the KTTK is organized by 6 constructs which includes Speed of Processing, Working Memory, Episodic Memory, Reasoning, Vocabulary, and Verbal Fluency.

Each of the constructs has various tasks associated with it and there are a total of 30 tasks that can be found listed below. To access any of the tasks within each construct, select the task of interest. The key to the names and data structure used for data coding of each construct spreadsheet is also included in this document and can be accessed by selecting “Data Coding Sheet” included under each construct listed below. Finally, the spreadsheet for each construct can be found listed below and accessed by selecting “Spreadsheet of data” listed under each construct.

Task and Assessment Numbering*

1 Speed of Processing Construct
- Task 1: Digit Comparison
- Task 2: WAIS-III Digit Symbol
- Task 3: NIH Toolbox Pattern Comparison Processing Speed Test
- Data Coding Sheet for Speed of Processing
- Spreadsheet of Speed of Processing Data

2 Working Memory
- Task 4: CANTAB Spatial Working Memory
- Task 5: WAIS-III Letter Number Sequencing Task
- Task 6: Operation Span Task
- Task 7: NIH Toolbox List Sorting
- Task 8: CANTAB Delayed Matching to Sample Task
- Task 9: CANTAB Spatial Recognition Memory Task
- Data Coding Sheet for Working Memory
- Spreadsheet of Working Memory data

4 Episodic Memory
- Task 15: Hopkins Verbal Learning, Parts 1-4 (Immediate & Delayed Recall)
- Task 16: CANTAB Verbal Recognition Memory Parts 1-4
- Task 17: Woodcock-Johnson Memory for Names Immediate & Delayed
- Task 18: Wechsler Memory Scale Logical Memory
- Task 19: NIH Toolbox Picture Sequence Memory, Parts 1-2
- Data Coding Sheet for Episodic Memory
- Spreadsheet of Episodic Memory data

5 Reasoning
- Task 20: Raven’s Matrices
- Task 21: ETS Letter Sets
- Task 22: CANTAB Stockings of Cambridge

Task 23: Everyday Problem Solving
Data Coding Sheet for Reasoning
Spreadsheet of Reasoning data

6 Vocabulary

Task 24: Educational Testing Service Advanced Vocabulary
Task 25: Shipley Vocabulary
Task 26: CANTAB Graded Naming Task
Task 27: NIH Toolbox Oral Reading Recognition Test
Task 28: NIH Toolbox Picture Vocabulary
Data Coding Sheet for Vocabulary
Spreadsheet of Vocabulary data

7 Verbal Fluency

Task 29: Controlled Oral Word Association (FAS)
Task 30: Controlled Oral Association: Categories
Data Coding Sheet for Verbal Fluency
Spreadsheet of Verbal Fluency data

● ***Note:*** Construct numbering skips "3", as it corresponds to an additional construct (Executive Function) that was included in the original design of the DLBS, but was dropped from the study due to persistent data collection and task availability issues. The partial data for this construct is not included in this dataset, but can be made available upon request.

Construct 1: Speed of Processing

Definition
This construct measures how rapidly individuals can perceptually compare and process information (Park, 2000 in D.C. Park & N. Schwartz (Eds.)). It is highly sensitive to cognitive function and is considered a basic core component of cognition. Timothy Salthouse authored a classic paper that fully describes both the theoretical importance and empirical measures of speed of processing (Salthouse, 1996).

References
Salthouse, T.A. (1996). The processing-speed theory of adult age differences in cognition. Psychological Review, 103, 403-428.
Park, D.C. (2000). The basic mechanisms accounting for age-related decline in cognitive function. In D.C. Park & N. Schwartz (Eds.), Cognitive Aging: A primer, pp. 3-21. Psychology Press.

Sample Sizes by Epoch and Task (subjects with partial data in parentheses)

Assessment	Epoch 1	Epoch 2	Epoch 3
**Digit Comparison**	463(1)	324	212
**WAIS-III Digit Symbol**	456	323	212
**NIH Toolbox Pattern Comparison Processing Speed**	0	322(322)[a]	206(17)[a]

Notes on data completeness:
[a]NIH Toolbox Pattern Comparison Processing Speed: for Epoch 2, the computed score is unavailable and the fully-corrected score is available for only 193 participants; for Epoch 3, the age-corrected, percentile, and fully-corrected scores were only available for 189 participants. The uncorrected standardized or raw scores are recommended for this task.

Task 1.1 Digit Comparison Task
Description (task duration: 2.5 minutes): Participants have to decide whether two number strings that are either 3, 6, or 9 digits in length, have identical digits or different digits. The task is divided into 3 separate sets; a set is comprised only of 3-digit, 6-digit, or 9-digit comparisons (i.e., a set doesn't contain a mix of different string lengths). Subjects are given 45 seconds for each set to try to complete as many comparisons as they can. The number correctly completed for 3-, 6-, and 9-item strings, as well as total correct, is available. Higher scores are better.

Primary Reference (Letter Comparison Task):
Salthouse T. A., & Babcock R. L. (1991). Decomposing adult age differences in working memory. *Developmental Psychology*, 27, 763-776.

Development of Cross-culturally Appropriate Measures (Digit Comparison Task)
Hedden, T., Park, D. C., Nisbett, R., Ji, LJ, Jing, Q., & Jiao, S. (2002). Cultural variation in verbal versus spatial neuropsychological function across the life span. *Neuropsychology*, 16, 65-73.

Task 1.2 WAIS-III Digit Symbol

Description (task duration: 1.5 minutes):

- Participants are shown nine geometric symbols that are each assigned to a digit from 1 to 9. They are then presented with randomized digits and asked to draw the corresponding symbol below each digit as quickly as possible for 90 seconds.

Primary Reference:
Wechsler, D. (1997). *WAIS-III: Administration and scoring manual: Wechsler Adult Intelligence Scale*. San Antonio, TX: Psychological Corporation.

Task 1.3 NIH Toolbox Pattern Comparison Processing Speed Test

Description (task duration: 1.5 minutes): Participants are shown two pictures side-by-side and are asked to discern whether the pictures are the same or different. If the pictures are the same, the participant presses the "Yes" button. If the pictures are not the same, the participant presses the "No" button. The participant is instructed to only use their index finger on their dominant hand to press either button. Participants' raw score is the number of 130 items correct in an 85-second period. The items are designed to be simple to most purely measure processing speed. Higher scores reflect faster speeds of processing.

● ***Caution:*** Participants in DLBS Epoch 2 performed the NIH Toolbox Pattern Comparison Speed Test on a desktop computer, whereas participants in DLBS Epoch 3 performed the task on an iPad. NIH toolbox provides a computed score to equate the different platforms (desktop and iPad) used. For additional details, we refer you to the NIH Toolbox website: https://www.healthmeasures.net/explore-measurement-systems/nih-toolbox/obtain-and-administer-measures.

Primary Reference:
Gershon RC, Wagster MV, Hendrie HC, Fox NA, Cook KF, Nowinsky CJ. NIH Toolbox for Assessment of Neurological and Behavioral Function. Neurology. 2013; 80: S1-S92.

Software Reference:
NIH Toolbox for the iPad test ver. 2.1 https://nihtoolbox.force.com/s/article/nih-toolbox-scoring-and-interpretation-guide

Speed of Processing Ability Construct: Key to Names and Data Structure in Data Set

Item Name	Abbreviation	Description	Measurement
Subject Number	S#	Subject identifier	
Age Interval	AgeInterval	Age at Epoch recoded into 3-year intervals	20-100
Sex	Sex	Participant's biological sex.	m = Male

			f = Female
Race	Race	Race that the participant self-identifies with.	1 = Asian American/ Pacific Islander<br>2 = Black/African American<br>3 = Multiracial<br>4 = Native American<br>5 = White/Caucasian<br>6 = Other<br>7 = Unknown
Ethnicity	Ethnicity	Ethnicity that the participant self-identifies with.	1 = Hispanic/Latin(o/a)<br>0 = Non-Hispanic
Handedness Score	HandednessScore	Average score of participant hand preference while completing various tasks. Higher scores indicate preference for the right hand.	Score Range: 0-4<br><br>0 = Always left<br>1 = Usually left<br>2 = No preference<br>3 = Usually right<br>4 = Always right
Mini-Mental State Exam Total	MMSE	Total # of items answered correctly.	Score Range: 0-30
Cognitive Battery Epoch 1-2 Interval	CogW1toW2	Interval between cognitive testing day 1 for Epochs 1-2.	# of Years
Cognitive Battery Epoch 2-3 Interval	CogW2toW3	Interval between cognitive testing day 1 for Epochs 2-3.	# of Years
Cognitive Battery Epoch 1-3 Interval	CogW1toW3	Interval between cognitive testing day 1 for Epochs 1-3.	# of Years
Take Home Epoch 1-2 Interval	TakeHomeW1toW2	Interval between Take Home for Epochs 1-2.	# of Years
Take Home Epoch 2-3 Interval	TakeHomeW2toW3	Interval between Take Home for Epochs 2-3.	# of Years
Take Home Epoch 1-3 Interval	TakeHomeW1toW3	Interval between Take Home for Epochs 1-3.	# of Years
MRI Epoch 1-2 Interval	MRIW1toW2	Interval between MRI scan for Epochs 1-2.	# of Years
MRI Epoch 2-3 Interval	MRIW2toW3	Interval between MRI scan for Epochs 2-3.	# of Years
MRI Epoch 1-3 Interval	MRIW1toW3	Interval between MRI scan for Epochs 1-3.	# of Years
Amyloid PET Epoch 1-2 Interval	PETAmyW1toW2	Interval between amyloid PET scan for Epochs 1-2.	# of Years
Amyloid PET Epoch 2-3 Interval	PETAmyW2toW3	Interval between amyloid PET scan for Epochs 2-3.	# of Years
Amyloid PET Epoch 1-3 Interval	PETAmyW1toW3	Interval between amyloid PET scan for Epochs 1-3.	# of Years
Highest Level of Education Completed	EduComp5	This is an ordinal measure of participants' self-reported highest level of education completed.	1 = Less than high school graduate<br>2 = High school graduate/GED<br>3 = Some college/trade/ technical/business school<br>4 = Bachelor's degree<br>5 = Some graduate work<br>6 = Master's degree

			7 = MD/JD/PhD/other advanced degree
Education Estimated Years Capped	EduYrsEstCap5	This is a conversion of the participant's self-reported highest level of education into a capped estimated number of years it would take to reach this highest level of education.<br><br>The "capped" comes into play when someone spend a longer time than usual for a certain degree but did not complete it. In short, someone with a lot of years of education but did not complete a degree will not score higher than someone who did complete the degree.	*11 maximum* = Less than High school<br>*12* = High School<br>*15 maximum* = Some College<br>*16* = Bachelor’s degree<br>*20 maximum* = Some Graduate Work<br>*18* = Master’s degree<br>*21* = MD/JD/PhD/ Advanced degree
Construct Name	ConstructName	Speed of Processing	
Construct Number	ConstructNumber	Construct 1	
Epoch	Wave	Denotes the data collection Epoch.<br>See individual differences data set for more detail, including testing date intervals.	1 = Epoch 1<br>2 = Epoch 2<br>3 = Epoch 3
Has Data	HasData	1 = Yes, returned for Epoch<br>2 = No, did not return for Epoch	
Number of Tasks in Construct	NumTasks	How many tasks make up the speed of processing construct	3 Tasks for Speed of Processing
**Task 1—Digit Comparison**	**Task1**	1 = Has data<br>2 = Task data partial<br>3 = No task data	
Digit Comparison 3	DigComp3_1	Total correct for 3-digit strings	Score Range:<br>0-64
Digit Comparison 6	DigComp6_1	Dependent Variable: total correct for 6-digit strings	Score Range:<br>0-64
Digit Comparison 9	DigComp9_1	Dependent Variable: total correct for 9-digit strings	Score Range:<br>0-64
Digit Comparison Total	DigCompTotal1	Dependent Variable: Total correct summed across T3, T6, and T9 trials	Score Range:<br>0-192
**Task 2—Digit Symbol**	**Task2**	1 = Has data<br>2 = Task data partial<br>3 = No task data	
Digit Symbol Total	DigSymTotal2	Number of items matched correctly in 90 sec	Score Range:<br>0-93
**Task 3—NIH Toolbox Pattern Comparison Speed Test**	**Task3**	1 = Has data<br>2 = Task data partial<br>3 = No task data	
NIH Toolbox Pattern Comparison Speed Test	NIHSpeedRaw3	The participant’s raw score is the number of items answered correctly in 85 seconds of response time, with a range of 0-130. This score is then converted	Score Range:<br>0-130

		to the NIH Toolbox normative standard scores.	
NIH Toolbox Pattern Comparison Speed Test	NIHSpeedComp3	The computed score is a conversion between the desktop and iPad data present in Epoch 3.	Score Range: 0-130
NIH Toolbox Pattern Comparison Speed Test	NIHSpeedUn3	It compares the performance of the test-taker to those in the entire NIH Toolbox nationally representative normative sample, regardless of age or any other variable.	Normative Mean = 100, Standard Deviation = 15
NIH Toolbox Pattern Comparison Speed Test	NIHSpeedAge3	This score compares the score of the test-taker to those in the NIH Toolbox nationally representative normative sample at the same age, where a score of 100 indicates performance that was at the national average for the test-taking participant's age. Age-corrected standard scores were derived for adults (ages 18-85).	Mean = 100, Standard Deviation = 15
NIH Toolbox Pattern Comparison Speed Test	NIHSpeedPercent3	A Percentile represents the percentage of people nationally above whom the participant's score ranks (the comparison group will be based on whichever normative score is used)	Percentile Rank: 0-100
NIH Toolbox Pattern Comparison Speed Test	NIHSpeedFully3	This score compares the score of the test-taker to those in the NIH Toolbox nationally representative normative sample, while adjusting for key demographic variables (education, gender, and race/ethnicity) collected during the NIH Toolbox national norming study.	Mean = 50, Standard Deviation = 10

Construct 2: Working Memory

Definition

The construct of working memory measures the ability of individuals to simultaneously manipulate and store information. This ability plays a key role in processes involving language comprehension, reasoning, and planning, highlighting its importance in analyzing cognition. Baddeley and Hitch present the initial transition from the classically accepted short-term memory to the current definition of working memory.

References

Baddeley, A.D., & Hitch, G. (1974). Working Memory. *Psychology of Learning and Motivation*, *8*, 47-89. https://doi.org/10.1016/S0079-7421(08)60452-1

Salthouse, T. A., & Babcock, R. L. (1991). Decomposing adult age differences in working memory. *Developmental Psychology*, *25*(5), 763-776. https://doi.org/10.1037/0033-295X.103.3.403

Park, D.C. (2000). The basic mechanisms accounting for age-related decline in cognitive function. In D.C. Park & N. Schwartz (Eds.), *Cognitive Aging: A primer*, pp. 47-89. Psychology Press.

Sample Sizes by Epoch and Task (subjects with partial data in parentheses)

Assessment	Epoch 1	Epoch 2	Epoch 3
**CANTAB Spatial Working Memory**	463	333(1)	212
**WAIS-III Letter Number Sequencing Task**	463	321	212
**Operation Span Task**	461	94	206
**NIH Toolbox List Sorting**	0	316(230)[a]	206(17)[a]
**CANTAB Delayed Matching to Sample Task**	463	0	0
**CANTAB Spatial Recognition Memory Task**	463	0	0

Notes on data completeness:
[a]NIH Toolbox List Sorting: For Epoch 2, raw scores are only available for 90 participants and fully-corrected scores are only available for 198 participants. For Epoch 3, age-corrected, percentile, and fully-corrected scores are only available for 189 participants. The uncorrected standardized scores are recommended for this task.

Task 2.4 CANTAB Spatial Working Memory

Description (task duration: 10 minutes): This task assesses the participant's ability to retain spatial information while simultaneously manipulating remembered items in working memory. The objective of the task is to remember the sequence of locations where blue tokens were found (spatial memory), and simultaneously continue searching for tokens (processing component) without revisiting locations that have already yielded a token (memory component). Participants collect blue tokens that are hidden in an array of boxes (set size from 3-8 boxes). When an array of boxes is presented, participants must touch each box in turn with a touch-screen stylus until one reveals a blue token inside (a "search"). Once a blue token has been found, the participant will place it in the depository column on the right side of the screen ("home"), which indicates how many tokens remain in the sequence. The participant must search for the remaining tokens until one has been found in each box on the current screen. The boxes will remain on the screen after being touched, regardless of whether the participant locates blue tokens inside them. The participant's task is to remember where they find the tokens because, once a token has been found in a box, that box will never contain another token for that set. After a participant has found all blue tokens for an array of boxes, the task moves onto the next trial of boxes in a different array. This task contains 15 total trials. Trials 1-3 contain 3 boxes each, trials 4-7 contain 4 boxes each, trials 8-11 contain 6 boxes each, and trials 12-15 contain 8 boxes each. Performance in the more difficult trials of this task is enhanced by the use of a heuristic search strategy, which indicates that participants did not choose boxes at random.

Performance Errors: Two kinds of errors can be made: *Between (memory) errors* are when a participant returns to a box in which a token has already been found during the same search trial. Lower between error scores are better.
Within errors are when participants search any particular box more than once in the same search sequence. Lower within error scores are better.

Primary References:
Robbins T.W., et al. (1994). Cambridge Neuropsychological Test Automated Battery (CANTAB): A factor-analytic study of a large sample of normal elderly volunteers. *Dementia, 5*(5):266-281. https://doi.org/10.1159/000106735.
CANTAB Eclipse (2007). https://www.cambridgecognition.com/cantab/cognitive-tests/executive-function/spatial-executive-function-swm

Task 2.5 WAIS-III Letter-Number Sequencing

Description (task duration: 5 minutes): 7 blocks of 3 trials per block are presented unless task is terminated for poor performance. Participants listen to a series of numbers and letters (e.g., 1-J-A-6) and are asked to rearrange the items in their head and recite the sequence with the numbers first, in ascending order, followed by the letters, in alphabetical order (e.g., 1-6-A-J). Researchers present the letter-number strings at a rate of one letter or number per second. Responses are recorded for accuracy and each receives a score of correct (1) or incorrect (0). There are a total of 21 trials that range from 2-8-item strings and are presented from easiest to hardest. The task is terminated when a participant responds incorrectly to all three trials within a 3-item block. Otherwise, the researcher will continue and administer the next item of increased

difficulty (higher total number of letters and numbers) until all seven blocks are completed. The variables of interest are the number of trials answered correctly in each block and the total number of trials correct across all blocks. A higher score indicates better working memory performance.

Primary Reference:

Wechsler, D. (1997). *WAIS-III: Administration and scoring manual: Wechsler Adult Intelligence Scale*. San Antonio, TX: Psychological Corporation.

Task 2.6 Operation Span Task

Description (task duration: 20 minutes): Participants are presented with a simple arithmetic equation, and they respond "yes" if the equation is accurate (e.g., (6/3) +5=7) and "no" if it is inaccurate (e.g., (3+6)/3=2). As soon as the subject responds, the equation disappears, and a concrete noun is presented. Participants read the word aloud. Participants are told to take as much time as is needed to answer the arithmetic portion but must immediately read the ensuing word after answering. Immediately after they pronounce the word, the next arithmetic-word string appears on the screen. After a block of equations and words is complete (number of items in block varies from 2-5), participants are shown a screen with three question marks and prompted to write down all the words they remembered from that block in the order that they were presented. There are 12 blocks presented that each contain 2-5 items within a block, with a total of 42 arithmetic-word strings. Blocks are randomly ordered but do not vary between participants. Variables of interest are total number of words correctly recalled for each of the four block sizes (2, 3, 4, 5) and total recalled, with a higher number of recalled words indicative of better performance. Responses to the arithmetic portion of each set are not recorded and are not used for data analysis.

Primary Reference:

Turner, M. L., & Engle, R. W. (1989). Is working memory capacity task dependent? *Journal of Memory and Language*, *28*(2):127-154. https://doi.org/10.1016/0749-596X(89)90040-5

Task 2.7 NIH Toolbox List Sorting

Description (task duration: 10 minutes): Participants are presented with pictures of commonly known foods and animals that are displayed along with written text and an accompanying audio recording of the name of the item (e.g., "elephant"). Each picture is displayed on the screen, one at a time, in a "flashing" manner at a rate of 2 seconds per item. The objective is to reorder the block of pictures according to particular rules. In the one-list condition, participants sort each block of items by size and in the two-list condition, items are sorted by both size as well as by category. The variable of interest is a sum of the total correct responses across both lists, with higher scores suggesting greater global working memory capacity.

One-List Condition: Participants are presented with a sequence of 2-7 pictures (*either* food or animals) and must order the series from smallest to largest. Participants answer verbally and must name all the items in the correct order without intrusions. The task begins with a 2-item block. If answered correctly, the number of items in each block will increase up to seven total items. If answered incorrectly, participants will get a second block of similar difficulty. If they then answer correctly for the second block, they advance to the next block of higher difficulty,

otherwise, the testing is terminated. This condition contains two practice blocks in which immediate feedback is provided and a maximum of 14 testing blocks.
Two-List Condition: Participants are presented with a series of *both* food and animals and must order the series by both size and category. Participants will sort the food items from smallest to largest, followed by the animal items from smallest to largest. This condition contains two practice blocks, in which feedback is provided, and a maximum of 12 testing blocks. The test procedure is identical to the one-list condition, only with the added complexity of sorting by both size and category.

Primary References:

Gershon, R.C., et al. (2013). NIH toolbox for assessment of neurological and behavioral function. *Neurology, 80*(11 Suppl 3): S2-6. https://doi.org/10.1212/WNL.0b013e3182872e5f

Tulsky, D.S., et al. (2013). NIH Toolbox Cognitive Function Battery (NIHTB-CFB): Measuring working memory. *Monographs of the Society for Research in Child Development*, *78*(4):70–87. https://doi.org/10.1111/mono.12035

Task 2.8 CANTAB Delayed Matching to Sample Task

Description (task duration: 12 minutes): This task measures maintenance of visual memory in a four-choice delayed recognition memory paradigm. Participants are presented with a complex, abstract target pattern that consists of four quadrants differing in color and form and they must match the target to one of four choice patterns. One of the choice patterns is identical to the target, one is a novel distractor pattern, one has the shape of the sample and the colors of the distractor, and the fourth has the colors of the sample and the shape of the distractor. All four choice patterns have at least one quadrant in common with the sample. There a four different choice conditions: (1) choices added to the screen with the target, (2) choices shown 0 seconds after the target disappears, (3) choices are shown 4 seconds after the target pattern disappears, and (4) choices are shown 12 seconds after the target pattern disappears. Participants are asked to select the choice pattern that matches the presented sample pattern by touching their response with a touch-screen stylus. Feedback is provided on-screen for incorrect responses and participants continue their search until they find the matching pattern. The variables of interest include the total number of items matched correctly across each delay period (simultaneous, 0 second delay, 4 second delay, and 12 second delay) and the total number of correctly matched items across all delayed periods. Higher scores suggest a more efficient visual memory ability.

Primary References:

Robbins T.W., et al. (1994). Cambridge Neuropsychological Test Automated Battery (CANTAB): A factor-analytic study of a large sample of normal elderly volunteers. *Dementia, 5*(5):266-281. https://doi.org/10.1159/000106735.
CANTAB Eclipse (2007). https://www.cambridgecognition.com/cantab/cognitive-tests/executive-function/spatial-executive-function-swm

Task 2.9 CANTAB Spatial Recognition Memory Task

Description (task duration: 5 minutes): This task measures visual-spatial recognition memory in a two-choice forced discrimination paradigm. The task has two phases: spatial encoding followed by recognition. In the encoding phase, participants are shown a white square that moves sequentially to five different locations on the screen, each for three seconds. After a five second delay, subjects are presented with two white squares for the recognition phase. One of them occupies a location where a square was presented during encoding and the other square is in a novel location (distractor stimulus). Participants are asked to select the square that is in the location previously seen in the encoding phase. There are four blocks of five trials, for a total of 20 responses. The variable of interest is total number of locations correctly identified with higher scores indicating better working memory performance.

Primary References:

Robbins T.W., et al. (1994). Cambridge Neuropsychological Test Automated Battery (CANTAB): A factor-analytic study of a large sample of normal elderly volunteers. *Dementia, 5*(5):266-281. https://doi.org/10.1159/000106735.

CANTAB Eclipse (2007). https://www.cambridgecognition.com/cantab/cognitive-tests/executive-function/spatial-executive-function-swm

Working Memory Data Set: Key to Names and Data Structure in Data Set

Item Name	Abbreviation	Description	Measurement
Subject Number	S#	Subject identifier	
Age Interval	AgeInterval	Age at Epoch recoded into 3-year intervals	20-100
Sex	Sex	Participant's biological sex.	m = Male<br>f = Female
Race	Race	Race that the participant self-identifies with.	1 = Asian American/ Pacific Islander<br>2 = Black/African American<br>3 = Multiracial<br>4 = Native American<br>5 = White/Caucasian<br>6 = Other<br>7 = Unknown
Ethnicity	Ethnicity	Ethnicity that the participant self-identifies with.	1 = Hispanic/Latin(o/a)<br>0 = Non-Hispanic
Handedness Score	HandednessScore	Average score of participant hand preference while completing various tasks. Higher scores indicate preference for the right hand.	Score Range: 0-4<br><br>0 = Always left<br>1 = Usually left<br>2 = No preference<br>3 = Usually right<br>4 = Always right
Mini-Mental State Exam Total	MMSE	Total # of items answered correctly.	Score Range: 0-30
Cognitive Battery Epoch 1-2 Interval	CogW1toW2	Interval between cognitive testing day 1 for Epochs 1-2.	# of Years

Cognitive Battery Epoch 2-3 Interval	CogW2toW3	Interval between cognitive testing day 1 for Epochs 2-3.	# of Years
Cognitive Battery Epoch 1-3 Interval	CogW1toW3	Interval between cognitive testing day 1 for Epochs 1-3.	# of Years
Take Home Epoch 1-2 Interval	TakeHomeW1toW2	Interval between Take Home for Epochs 1-2.	# of Years
Take Home Epoch 2-3 Interval	TakeHomeW2toW3	Interval between Take Home for Epochs 2-3.	# of Years
Take Home Epoch 1-3 Interval	TakeHomeW1toW3	Interval between Take Home for Epochs 1-3.	# of Years
MRI Epoch 1-2 Interval	MRIW1toW2	Interval between MRI scan for Epochs 1-2.	# of Years
MRI Epoch 2-3 Interval	MRIW2toW3	Interval between MRI scan for Epochs 2-3.	# of Years
MRI Epoch 1-3 Interval	MRIW1toW3	Interval between MRI scan for Epochs 1-3.	# of Years
Amyloid PET Epoch 1-2 Interval	PETAmyW1toW2	Interval between amyloid PET scan for Epochs 1-2.	# of Years
Amyloid PET Epoch 2-3 Interval	PETAmyW2toW3	Interval between amyloid PET scan for Epochs 2-3.	# of Years
Amyloid PET Epoch 1-3 Interval	PETAmyW1toW3	Interval between amyloid PET scan for Epochs 1-3.	# of Years
Highest Level of Education Completed	EduComp5	This is an ordinal measure of participants’ self-reported highest level of education completed.	1 = Less than high school graduate<br>2 = High school graduate/GED<br>3 = Some college/trade/ technical/business school<br>4 = Bachelor’s degree<br>5 = Some graduate work<br>6 = Master’s degree<br>7 = MD/JD/PhD/other advanced degree
Education Estimated Years Capped	EduYrsEstCap5	This is a conversion of the participant's self-reported highest level of education into a capped estimated number of years it would take to reach this highest level of education.<br><br>The "capped" comes into play when someone spend a longer time than usual for a certain degree but did not complete it. In short, someone with a lot of years of education but did not complete a degree will not score higher than someone who did complete the degree.	*11 maximum* = Less than High school<br>*12* = High School<br>*15 maximum* = Some College<br>*16* = Bachelor’s degree<br>*20 maximum* = Some Graduate Work<br>*18* = Master’s degree<br>*21* = MD/JD/PhD/ Advanced degree
Construct Name	ConstructName	Working Memory	
Construct Number	ConstructNumber	Construct 2	
Epoch	Wave	Denotes the data collection Epoch. See individual differences data set for more detail, including testing date intervals.	1 = Epoch 1<br>2 = Epoch 2<br>3 = Epoch 3

Has Data	HasData	Yes = 1<br>No = 2	
Number of Tasks in Construct	NumTasks	How many tasks make up the working memory construct	6 tasks for Working Memory
**Task 4-CANTAB Spatial Working Memory**	**Task4**	1 = Has data<br>2 = Task data partial<br>3 =No task data	
Spatial WM 4-Box Errors*	SptlWM4BoxErrs4	Total errors for 4-box trials	No max score
Spatial WM 6-Box Errors*	SptlWM6BoxErrs4	Total errors for 6-box trials	No max score
Spatial WM 8-Box Errors*	SptlWM8BoxErrs4	Total errors for 8-box trials	No max score
Spatial WM Total Errors	SptlWMTotErrs4	Total number of errors across all trials.	No max score
**Task 5 – WAIS Letter-Number Sequencing**	**Task5**	1 = Has data<br>2 = Task data partial<br>3 =No task data	
Letter-Number Sequencing 2-Item Trials Total Correct	LetNumSeq2Item5	Total number of 2-item trials recalled correctly	Score Range: 0-3
Letter-Number Sequencing 3-Item Trials Total Correct	LetNumSeq3Item5	Total number of 3-item trials recalled correctly	Score Range: 0-3
Letter-Number Sequencing 4-Item Trials Total Correct	LetNumSeq4Item5	Total number of 4-item trials recalled correctly	Score Range: 0-3
Letter-Number Sequencing 5-Item Trials Total Correct	LetNumSeq5Item5	Total number of 5-item trials recalled correctly	Score Range: 0-3
Letter-Number Sequencing 6-Item Trials Total Correct	LetNumSeq6Item5	Total number of 6-item trials recalled correctly	Score Range: 0-3
Letter-Number Sequencing 7-Item Trials Total Correct	LetNumSeq7Item5	Total number of 7-item trials recalled correctly	Score Range: 0-3
Letter-Number Sequencing 8-Item Trials Total Correct	LetNumSeq8Item5	Total number of 8-item trials recalled correctly	Score Range: 0-3
Letter-Number Sequencing Total	LetNumSeqTot5	Total number of trials recalled correctly	Score Range: 0-21
**Task 6 – Operation Span**	**Task6**	1 = Has data<br>2 = Task data partial<br>3 =No task data	
OSpan 2-Item Block Total (Blocks 4,7,11)	OSp2BLTot6	Total # of blocks recalled correctly for 2-item blocks	Score Range: 0-3

OSpan 3-Item Block Total (Blocks 1,3,9)	OSp3BLTot6	Total # of blocks recalled correctly for 3-item blocks	Score Range: 0-3
OSpan 4-Item Block Total (Blocks 6,8,12)	OSp4BLTot6	Total # of blocks recalled correctly for 4-item blocks	Score Range: 0-3
OSpan 5-Item Block Total (Blocks 2,5,10)	OSp5BLTot6	Total # of blocks recalled correctly for 5-item blocks	Score Range: 0-3
OSpan Total	OSpanTot6	Sum of total # of words recalled correctly for *perfectly recalled* blocks<br>To get a score of 42, subjects must correctly recall: Three two-item blocks (3 blocks x 2 words each = 6), three three-item blocks (3 blocks x 3 words each = 9), three four-item blocks (3 blocks x 4 words each = 12), three five-item blocks (3 blocks x 5 words each = 15); total number of words correct is thus 42.	Score Range: 0-42
**Task 7 – NIH Toolbox List Sorting**	**Task7**	1 = Has data<br>2 = Task data partial<br>3 =No task data	
NIH Toolbox List Sorting Raw Score	LstSrtRaw7	Scored sum of total # of items correctly recalled and sequenced on both lists	Score Range: 0-26
NIH Toolbox List Sorting Uncorrected Standard Score	LstSrtUn7	This score compares the performance of the test-taker to those in the entire NIH Toolbox nationally representative normative sample, regardless of age or any other variable.	Normative Mean = 100,<br>Standard Deviation = 15
NIH Toolbox List Sorting Age-Corrected Standard Score	LstSrtAge7	This score compares the score of the test-taker to those in the NIH Toolbox nationally representative normative sample at the same age, where a score of 100 indicates performance that was at the national average for the test-taking participant's age. Age-corrected standard scores were derived for adults (ages 18-85).	Mean = 100,<br>Standard Deviation = 15
NIH Toolbox List Sorting National Percentile	LstSrtPercent7	A Percentile represents the percentage of people nationally above whom the participant's score ranks (the comparison group will be based on whichever normative score is used).	Percentile Rank: 0-100
NIH Toolbox List Sorting Fully-Corrected T-score	LstSrtFully7	This score compares the score of the test-taker to those in the NIH Toolbox nationally representative normative sample, while adjusting for key demographic	Mean = 50,<br>Standard Deviation = 10

		variables (education, gender, and race/ethnicity) collected during the NIH Toolbox national norming study.	
**Task 8 – CANTAB Delayed Matching to Sample**	**Task8**	1 = Has data<br>2 = Task data partial<br>3 =No task data	
Delayed Matching to Sample Simultaneous Total*	DMSSimTot8	Total # of items matched correctly with simultaneous presentation	Score Range: 0-10
Delayed Matching to Sample Immediate (0 second delay) Total*	DMSImmTot8	Total # of items matched correctly with immediate (0 second delay) presentation	Score Range: 0-10
Delayed Matching to Sample 4 Second Delay Total*	DMS4SecTot8	Total # of items matched correctly with 4 second delay presentation	Score Range: 0-10
Delayed Matching to Sample 12 Second Delay Total*	DMS12SecTot8	Total # of items matched correctly with 12 second delay presentation	Score Range: 0-10
Delayed Matching to Sample Total	DMSTot8	Total # of items matched correctly	Score Range: 0-40
**Task 9 – CANTAB Spatial Recognition Memory**	**Task9**	1 = Has data<br>2 = Task data partial<br>3 =No task data	
Spatial Recognition Memory Total	SRMTot9	Total # of locations correctly identified	

Construct 4: Episodic Memory

Definition

This construct measures how well individuals can store, maintain, and retrieve detailed information in long-term memory. It is highly sensitive to normal aging processes and shows robust deficits in mild cognitive impairment and Alzheimer's disease (Koen & Yonelinas, 2014). Two classic papers by Endel Tulving (1972, 2002) provide both a theoretical conceptualization of episodic memory and relevant empirical measures.

References

Koen, J. D., & Yonelinas, A. P. (2014). The effects of healthy aging, amnestic mild cognitive impairment, and Alzheimer's disease on recollection and familiarity: A meta-analytic review. *Neuropsychology Review*, 24(3), 332-354. https://doi.org/10.1007/s11065-014-9266-5

Tulving, E. (1972). Episodic and semantic memory. *Organization of memory, 1*, 381-403.

Tulving, E. (2002). Episodic memory: From mind to brain. *Annual Review of Psychology, 53*, 1–25. https://doi.org/10.1146/annurev.psych.53.100901.135114

● ***Note:*** For all included memory tasks, the same item lists were used at each Epoch of data collection as there was an approximately 4-year interval between testing sessions.

Sample Sizes by Epoch and Task (subjects with partial data in parentheses)

Assessment	Epoch 1	Epoch 2	Epoch 3
**Hopkins Verbal Learning**	463(37)[a]	323(2)	213
**CANTAB Verbal Recognition Memory**	463(2)	335(335)[b]	212(212)[b]
**Woodcock-Johnson Memory for Names**	251(1)[c]	322(5)	212(2)
**Wechsler Memory Scale Logical Memory**	0	331	213(3)[a]
**NIH Toolbox Picture Sequence Memory**	0	322(144)[d]	206(17)[d]

Notes on data completeness:
[a]Hopkins Verbal Learning: For Epoch 1, the delayed recall and recognition tests were only administered to 426 participants.
[b]CANTAB Verbal Recognition Memory: Epoch 2-3 data for the delayed recall portion of the CANTAB VRM task are unavailable as administration of that test was discontinued due to extremely skewed score distributions. Use of the immediate recall score is recommended.
[c]Woodcock-Johnson Memory for Names: Administration of the Woodcock-Johnson began partway through Epoch 1, thus data were not collected for approximately the first half of participants.
[d]NIH Toolbox Picture Sequence Memory: For Epoch 2, the fully-corrected score is only available for 191 participants. For Epoch 3, the age-corrected, percentile, and fully-corrected scores are only available for 189 participants. The uncorrected standardized scores or raw scores are recommended for this task.

Task 4.15 Hopkins Verbal Learning, Parts 1-4

Description (task duration: 6 minutes):

- **Encoding:** Participants memorize a semantically categorized list of 12 concrete nouns that are read aloud by the experimenter at a rate of one word every 1.5 seconds. The three semantic categories are sports, professions, and vegetables, with 4 words in each category.
- **Immediate Recall:** Immediately following the presentation, participants are asked to recall aloud as many words from the list as they can in any order. The experimenter records the words recalled on a scoring sheet. The dependent measure is the number of items correctly recalled out of 12.

- **<u>Delayed Recall</u>:** After approximately 20 minutes, participants are again asked to recall aloud as many words as possible from the previous list in any order. The experimenter records the words recalled on a scoring sheet. The dependent measure is the number of items correctly recalled out of 12.
- **<u>Delayed Recognition</u>:** Following delayed recall, participants are given a recognition test in which the experimenter reads another list of 24 words, including 12 target words (from the recall list) and 12 new words (lures). Of the 12 lures, 6 are semantically related to the target items (2 for each semantic category) and 6 are not semantically related to the target items. Participants make "yes"/"no" judgments to indicate if the word was on the original study list. The dependent measure is the total number of correct judgments (including hits + correction rejections) out of 24. In addition, false alarm rates are available for related and unrelated items, as are hits to old items.

Primary Reference:

Brandt, J. (1991). The Hopkins Verbal Learning Test: Development of a new memory test with six equivalent forms. *The Clinical Neuropsychologist*, *5*(2), 125-142. https://doi.org/10.1080/13854049108403297

<u>Task 4.16 CANTAB Verbal Recognition Memory, Parts 1-4</u>

Description (task duration: 7 minutes):

- **<u>Encoding</u>**: Twelve nouns are presented on the computer screen one at a time. Participants are asked to read each word aloud and remember as many as they can.
- **<u>Immediate Recall</u>:** Immediately following the presentation of the word list, participants are asked to recall aloud as many of the words as possible in any order. Data for the number of items recalled, out of 12, are available.
- **<u>Immediate Recognition</u>:** Immediately following recall, participants complete a recognition test in which the computer displays the 12 target items and 12 distractor items, one at a time. Participants answer whether they remember seeing the item earlier in the task on the computer ("yes" or "no"). Performance was near ceiling for this test, and data are not currently processed/checked.
- **<u>Delayed Recognition</u>:** The recognition phase is repeated after a delay of approximately 40 minutes. Data for the number of items recognized (and correct rejections), out of 24, are available. Performance was near ceiling for this task and we advise against using it but include it to provide a complete accounting of the methodology.

Primary Reference:

Robbins, T.W., et al. (1994). Cambridge Neuropsychological Test Automated Battery (CANTAB): A factor analytic study of a large sample of normal elderly volunteers. *Dementia, 5*, 266-281. https://doi.org/10.1159/000106735

Software Reference:

CANTAB Eclipse. *Cambridge Cognition* (2007). https://www.cambridgecognition.com/cantab/cognitive-tests/memory/verbal-recognition-memory-vrm/

● ***Note:*** It is recommended that the delayed recognition score (CantabVrmDelayRcg16) should NOT be used as it has strong ceiling effects, resulting in severe skewness and kurtosis. Standard data transformations were unable to correct this issue.

Task 4.17 Woodcock-Johnson Memory for Names, Parts 1-3

Description (task duration: 15 minutes):

- **Task Overview:** In this paired-associate recognition task, participants are given 12 trials that each include both an encoding and recognition component. This task is administered using color illustrations in a printed flip-book. On each trial, the participant first learns the name of a single cartoon space creature. Then, they must identify that creature in an array of nine aliens. Finally, they are asked to identify previously learned creatures in that array. The difficulty increases across trials as participants are required to remember the names of an increasingly larger set of creatures (up to 12 unique creatures). A separate delayed recognition test is administered 20 minutes later.
- **Encoding:** For the encoding component of each trial, participants are shown a color illustration of the space creature by itself on a page. Participants are told the name of the creature and are asked to point to it on the page (e.g., "This is Meegoy. Point to Meegoy." .").
- **Immediate Recognition:** Next, for the recognition component of that trial, participants are shown a page of nine space creatures and are asked to point to the newly-introduced creature among the distractors ("Now point to Meegoy"). Then, they are asked to point to previously learned creatures ("Now point to Kiptron"). For each trial, the previously learned creatures are tested in a novel order, and whenever the participant responds incorrectly, they are corrected (e.g., "No, this is Meegoy. Point to Meegoy."). For each trial, they are tested on all previously learned creatures up to a total of 9 creatures; for trials 10-12, the earliest creatures are dropped to keep that total at 9. Specifically, the total number of creatures to be recognized on each trial progresses across the 12 trials as follows: 1, 2, 3, 4, 5, 6, 7, 8, 9, 9, 9, 9 (total = 72 items). The dependent measure is the number of creatures recognized out of 72.
- **Delayed Recognition:** After a 20-minute delay, participants are given a surprise recognition test in which they are asked to point to each space creature when prompted by the experimenter. This delayed test has 3 parts, with 12 trials per part. In each trial, the participant is shown an array of nine space creatures, as before, and is asked to point to a previously learned creature ("Now point to Meegoy"). Next, they are shown a new array and asked to point to a different creature ("Now point to Kiptron"). In this test, incorrect responses are no longer corrected by the experimenter, and creatures are not presented in the order originally learned. This process repeats for part 1 until they have been asked to recognize all 12 unique space creatures in one of the 12 different arrays. For parts 2 and 3, they repeat this processing going through the 12 arrays in the same order, but with the items tested being put in a new order—for example, in trial 1 they may now be asked to identify "Delton" instead of "Meegoy". Thus, all 12 space creatures are tested 3 times each, and the dependent measure is the number of creatures recognized out of 36.

Primary Reference:

Woodcock, R. W., & Johnson, M. B. (1989). *Woodcock-Johnson Tests of Achievement*. Allen, TX: DLM Teaching Resources.

Task 4.18 Wechsler Memory Scale (WMS-III) Logical Memory, Parts 1-3

Description (task duration: 7 minutes):

- **Encoding:** The experimenter reads two highly detailed stories to the participant. One story describes a fictional character reporting a robbery and another describes a character listening to a weather bulletin.
- **Immediate Recall:** Immediately after each story, the participant is asked to recall as much of the story as they can, verbatim. The participant's response is recorded via a tape recorder. Reviewing the tape, the experimenter scores the participant's response by awarding one point per highly specific detail recalled by the participant (called Story Units, e.g., the main character's name is Anna, the story took place in Boston, the weather forecast predicted rain and hail, etc.). Story Unit scores for Story A and Story B (each out of 25) are calculated by summing all correct details (total out of 50).
- **Delayed Recall:** After a delay of approximately 30 minutes, the participant is asked to repeat as much of each of the two stories as they can remember with answers recorded. Story Unit scores for Story A and B (each out of 25) are again calculated, with a combined score out of 50.

Task Example:

Story A: This story involves a fictional character, Anna Thompson, reporting at a police station that she was robbed, including additional details about her profession and family. (length: 351 characters)

Story B: This story involves a fictional character, Joe Garcia, hearing a detailed weather bulletin about inclement weather and then Joe deciding to stay home for the day. (length: 470 characters)

Primary Reference:

Wechsler, D. (1997). *Wechsler memory scale (WMS-III)*. San Antonio, TX: Psychological Corporation.

● ***Note:*** The Logical Memory task can also be scored based on the participants' recall of seven or eight thematic details from the stories (e.g., broadly, indication of character's gender, indication of major events in the story – storm, robbery, etc.). The thematic score is not checked or verified and is not used.

Task 4.19 NIH Toolbox Picture Sequence Memory, Parts 1-2

Description (task duration: 7 minutes):

- **Encoding:** This test involves recalling increasingly lengthy series of illustrated objects and activities that are presented in a particular order on the computer screen. These picture sequences revolve around two scenarios: playing in a park and going camping. During encoding, each picture is presented individually in the center of the screen for approximately 5 s with pre-recorded instructions describing the image (e.g., "roasting a marshmallow") and the item then being placed below in a sequence mirroring presentation order (from left-to-right) (see below example).

- **Retrieval:** After all items are placed, these pictures are then returned to the center of the screen in a jumbled pattern, and the participant's task is to move them below again in the correct sequence. There are 15 items in the first trial, and 18 items in the second trial.
- **Scoring:** Participants are given credit for each adjacent pair of pictures that are put in the correct sequence, regardless of location. For example, if pictures in locations 7 and 8 are placed in that order and adjacent to each other anywhere–such as slots 1 and 2–one point is awarded. The maximum score for each trial is one less than the trial length, which equates to 14 points for trial 1 and 17 points for trial 2 (Total Score Range: 0-31). Multiple dependent variables are provided via NIH Toolbox: (1) a *raw score* is their combined score across the two trials (Score Range: 0-31), (2) a *computed score* uses item response theory to put everyone on a scale of 200-750, (3) an *unadjusted scale score* compares this computed score with the full NIH Toolbox nationally representative normative sample (normative *M* = 100, *SD* = 15) (4) an *age-adjusted scale score* compares the computed score of the test-taker to those in the NIH normative sample at the same age (*M* = 100, *SD* = 15), (5) an *age-adjusted national percentile* represents the percentage of people nationally above whom the participant's score ranks (using NIH normative sample), and (6) a *fully-adjusted scale score* further adjusts for key demographic variables from the NIH normative sample, including age, gender, race/ethnicity (white/Asian, black, Hispanic, multiracial), and educational attainment (*M* = 50, *SD* = 10; NIH Toolbox: Scoring and Interpretation Guide, 2016).

Primary Reference:

Dikmen, S. S., Bauer, P. J., Weintraub, S., Mungas, D., Slotkin, J., Beaumont, J. L., ... & Heaton, R. K. (2014). Measuring episodic memory across the lifespan: NIH Toolbox Picture Sequence Memory Test. *Journal of the International Neuropsychological Society, 20*(6), 611-619. https://doi.org/10.1017/S1355617714000460

Software Reference:

NIH Toolbox for the iPad test ver. 2.1 https://nihtoolbox.force.com/s/article/nih-toolbox-scoring-and-interpretation-guide

● ***Note:*** Participants in DLBS Epoch 2 performed the NIH Toolbox Picture Sequence Memory on a desktop computer, whereas, participants in DLBS Epoch 3 performed the task on an ipad. For additional details, we refer you to the the NIH Toolbox website: https://www.healthmeasures.net/explore-measurement-systems/nih-toolbox/obtain-and-administer-measures

Episodic Memory Data Set: Key to Names and Data Structure in Data Set

Item Name	Abbreviation	Description	Measurement
Subject Number	S#	Subject identifier	
Age Interval	AgeInterval	Age at Epoch recoded into 3-year intervals	20-100
Sex	Sex	Participant's biological sex.	m = Male<br>f = Female
Race	Race	Race that the participant self-identifies with.	1 = Asian American/ Pacific Islander<br>2 = Black/African American

			3 = Multiracial<br>4 = Native American<br>5 = White/Caucasian<br>6 = Other<br>7 = Unknown
Ethnicity	Ethnicity	Ethnicity that the participant self-identifies with.	1 = Hispanic/Latin(o/a)<br>0 = Non-Hispanic
Handedness Score	HandednessScore	Average score of participant hand preference while completing various tasks. Higher scores indicate preference for the right hand.	Score Range: 0-4<br><br>0 = Always left<br>1 = Usually left<br>2 = No preference<br>3 = Usually right<br>4 = Always right
Mini-Mental State Exam Total	MMSE	Total # of items answered correctly.	Score Range: 0-30
Cognitive Battery Epoch 1-2 Interval	CogW1toW2	Interval between cognitive testing day 1 for Epochs 1-2.	# of Years
Cognitive Battery Epoch 2-3 Interval	CogW2toW3	Interval between cognitive testing day 1 for Epochs 2-3.	# of Years
Cognitive Battery Epoch 1-3 Interval	CogW1toW3	Interval between cognitive testing day 1 for Epochs 1-3.	# of Years
Take Home Epoch 1-2 Interval	TakeHomeW1toW2	Interval between Take Home for Epochs 1-2.	# of Years
Take Home Epoch 2-3 Interval	TakeHomeW2toW3	Interval between Take Home for Epochs 2-3.	# of Years
Take Home Epoch 1-3 Interval	TakeHomeW1toW3	Interval between Take Home for Epochs 1-3.	# of Years
MRI Epoch 1-2 Interval	MRIW1toW2	Interval between MRI scan for Epochs 1-2.	# of Years
MRI Epoch 2-3 Interval	MRIW2toW3	Interval between MRI scan for Epochs 2-3.	# of Years
MRI Epoch 1-3 Interval	MRIW1toW3	Interval between MRI scan for Epochs 1-3.	# of Years
Amyloid PET Epoch 1-2 Interval	PETAmyW1toW2	Interval between amyloid PET scan for Epochs 1-2.	# of Years
Amyloid PET Epoch 2-3 Interval	PETAmyW2toW3	Interval between amyloid PET scan for Epochs 2-3.	# of Years
Amyloid PET Epoch 1-3 Interval	PETAmyW1toW3	Interval between amyloid PET scan for Epochs 1-3.	# of Years
Highest Level of Education Completed	EduComp5	This is an ordinal measure of participants' self-reported highest level of education completed.	1 = Less than high school graduate<br>2 = High school graduate/GED<br>3 = Some college/trade/ technical/business school<br>4 = Bachelor's degree<br>5 = Some graduate work<br>6 = Master's degree<br>7 = MD/JD/PhD/other advanced degree

Education Estimated Years Capped	EduYrsEstCap5	This is a conversion of the participant's self-reported highest level of education into a capped estimated number of years it would take to reach this highest level of education.<br><br>The "capped" comes into play when someone spend a longer time than usual for a certain degree but did not complete it. In short, someone with a lot of years of education but did not complete a degree will not score higher than someone who did complete the degree.	*11 maximum* = Less than High school<br>*12* = High School<br>*15 maximum* = Some College<br>*16* = Bachelor's degree<br>*20 maximum* = Some Graduate Work<br>*18* = Master's degree<br>*21* = MD/JD/PhD/ Advanced degree
Construct Name	ConstructName	Episodic Memory	
Construct Number	ConstructNumber	Construct 4	
Epoch	Wave	Denotes the data collection Epoch. See individual differences data set for more detail, including testing date intervals.	1 = Epoch 1<br>2 = Epoch 2<br>3 = Epoch 3
Has Data	HasData	1 = Yes, returned for Epoch;<br>2 = No, did not return for Epoch	
Number of Tasks in Construct	NumTasks	How many tasks make up the episodic memory construct	5 tasks for Episodic Memory
**Task 15—Hopkins Verbal Learning**	**Task15**	1 = Has data<br>2 = Task data partial<br>3 = No task data	
Hopkins immediate recall	HopImmRcll15	Total correctly recalled	Score Range: 0-12
Hopkins delayed recall	HopDelayRcll15	Total correctly recalled	Score Range: 0-12
Hopkins delayed recognition	HopRcgCrrct15	Total correct (hits + correct rejections)	Score Range: 0-24
Hopkins delayed recognition	HopRcgHit15	Total hits (calling old item old)	Score Range: 0-12
Hopkins delayed recognition	HopRcgFaRelat15	Total false alarms to distractors semantically related to target (calling new item old)	Score Range: 0-6
Hopkins delayed recognition	HopRcgFaUnrelat15	Total false alarms to distractors semantically unrelated to target (calling new item old)	Score Range: 0-6
Hopkins delayed recognition	HopRcgFaTotal15	Total false alarms to distractors (calling new item old)	Score Range: 0-12
Hopkins delayed recognition	HopRcgHitminusfa15	Total hits – false alarms	Score Range: -12-12

**Task 16—CANTAB Verbal Recognition Memory**	**Task16**	1 = Has data<br>2 = Task data partial<br>3 = No task data	
CANTAB Verbal Recognition immediate recall	CantabVrmImmRcll16	Total correctly recalled	Score Range: 0-12
CANTAB Verbal Recognition delayed	CantabVrmDelayRcg16	Total correctly recognized (hits + correct rejections)	Score Range: 0-24
**Task 17—Woodcock-Johnson Memory for Names**	**Task17**	1 = Has data<br>2 = Task data partial<br>3 = No task data	
Woodcock-Johnson immediate recognition	WjImm17	Total correctly recognized	Score Range: 0-72
Woodcock-Johnson delayed recognition	WjDelay17	Total correctly recognized	Score Range: 0-36
**Task 18—Wechsler Memory Scale Logical Memory**	**Task18**	1 = Has data<br>2 = Task data partial<br>3 = No task data	
Logical memory immediate recall	LmStoryAImm18	Total immediate Story A recall score	Score Range: 0-25
Logical memory immediate recall	LmStoryBImm18	Total immediate Story B recall score	Score Range: 0-25
Logical memory immediate recall	LmStoryImm18	Total immediate Story A+B recall score	Score Range: 0-50
Logical memory delayed recall	LmStoryADelay18	Total delayed Story A recall score	Score Range: 0-25
Logical memory delayed recall	LmStoryBDelay18	Total delayed Story B recall score	Score Range: 0-25
Logical memory delayed recall	LmStoryDelay18	Total delayed Story A+B recall score	Score Range: 0-50
**Task19--NIH Toolbox Picture Sequence Memory**	**Task19**	1 = Has data<br>2 = Task data partial<br>3 = No task data	
NIH Toolbox Picture Sequence Memory	NIHPicSeqRaw19	Total number of pictures placed in the correct sequence across both trials	Score Range: 0-31
NIH Toolbox Picture Sequence Memory	NIHPicSeqComp19	This computed score uses item response theory to put everyone on a scale of 200-750	Score Range: 200-750
NIH Toolbox Picture Sequence Memory	NIHPicSeqUn19	It compares the performance of the test-taker to those in the entire NIH Toolbox nationally representative normative sample, regardless of age or any other variable.	Normative Mean = 100,<br>Standard Deviation = 15
NIH Toolbox Picture Sequence Memory	NIHPicSeqAge19	This score compares the score of the test-taker to those in the NIH Toolbox nationally representative normative sample at the same age, where a score of	Mean = 100,<br>Standard Deviation = 15

		100 indicates performance that was at the national average for the test-taking participant’s age. Age-corrected standard scores were derived for adults (ages 18-85).	
NIH Toolbox Picture Sequence Memory	NIHPicSeqPercent19	A Percentile represents the percentage of people nationally above whom the participant’s score ranks (the comparison group will be based on whichever normative score is used)	Percentile Rank: 0-100
NIH Toolbox Picture Sequence Memory	NIHPicSeqFully19	This score compares the score of the test-taker to those in the NIH Toolbox nationally representative normative sample, while adjusting for key demographic variables (education, gender, and race/ethnicity) collected during the NIH Toolbox national norming study.	Mean = 50,<br>Standard Deviation = 10

Construct 5: Reasoning

Definition

The construct of reasoning measures an individual's ability to recognize novel patterns and the conceptual relationship among objects and effectively apply these patterns to solve similar problems.

References

Schaie, K. W., & Willis, S. L. (1986). Can decline in adult intellectual functioning be reversed? *Developmental Psychology, 22*(2), 223–232. https://doi.org/10.1037/0012-1649.22.2.223

Boron, J. B., Turiano, N. A., Willis, S. L., & Schaie, K. W. (2007). Effects of Cognitive Training on Change in Accuracy in Inductive Reasoning Ability *Journal of Gerontology: Psychological Sciences,* 62B (3), 179-186.

Sample Sizes by Epoch and Task (subjects with partial data in parentheses)

Assessment	Epoch 1	Epoch 2	Epoch 3
**Raven's Matrices**	426(361)	327(45)	212(28)
**ETS Letter Sets**	461(1)	320(4)	212(3)
**CANTAB Stockings of Cambridge**	463	334	212(2)
**Everyday Problem Solving**	0	322	0

Notes on data completeness:
[a]Raven's Matrices: For Epoch 1, completion times were available for only 65 participants. For Epoch 2, completion times were available for only 282 participants. For Epoch 3, completion times were available for only 184 participants.

Task 5.20 Raven's Matrices

Description (task duration: 15 minutes):

- Participants are presented with a set of geometric patterns that have a sequential structure with one piece missing. At the same time, they are also presented with an array of 6 or 8 geometric shape options. Participants must determine which pattern out of these 6 or 8 options is required to complete the visual pattern set.
- The problems are divided into 4 blocks. In the first two blocks, subjects chose the correct pattern out of 6 options; in the last two blocks, subjects choose the correct pattern out of 8 options. Within a block, problems are arranged by increasing difficulty, with problems 1 and 2 being the easiest, problems 3 and 4 being moderately difficult, and problems 5 and 6 the most difficult.
- Participants are given 15 minutes to complete 24 problems. We note that this is a modification of the original Raven's Matrices, which has a larger pattern set.

Primary Reference:

Raven, J., Raven, J. C., & Court, J. H. (1998a). Manual for Raven's Progressive Matrices and Vocabulary Scales. Section 1: General Overview. San Antonio, TX: Harcourt Assessment.

Task 5.21 ETS Letter Sets

Description (task duration: 14 minutes):

- Subjects are presented with 5 sets of letters; each set is made up of 4 letters. Four of the sets of letters are alike in some way, while the fifth set of letters does not follow the same rule. Subjects are asked to determine which set of letters does not follow the same rule as the other 4 sets of letters. Subjects are instructed to mark a line through the set of letters that does not follow the same rule as the other 4 sets of letters.
- Participants have a total of 14 minutes to complete 30 problems. The task is presented in 2 parts each part lasting 7 minutes with 15 problems to complete.
- Higher scores indicate better reasoning ability.

Primary Reference:

Ekstrom, R. B., French, J. W., Harman, H., & Derman, D. (1976). Kit of factor-referenced cognitive tests (rev. ed.). Princeton, NJ: Educational Testing Service.

Task 5.22 CANTAB Stockings of Cambridge

Description (task duration: approximately 15 minutes):

- Stockings of Cambridge is a computerized version of Tower of London (Shallice 1982) in which participants are shown a split screen with two displays each containing three colored balls.
- ***Task Phase:*** The balls are arranged in such a way that they look like they are stacked in stockings hanging from a beam. Participants must move the balls in the bottom arrangement one at a time in order to match the top arrangement in as few moves as possible.
- ***Motor Phase:*** The balls are arranged in the same way as in the task phase, but now the top arrangement and the bottom arrangement begin identical. The computer will automatically move a ball in the top arrangement. The participant should copy the same movement on the bottom arrangement, moving the same-colored ball to the same position that the computer moved the ball in the top arrangement.
- ***Practice Phase:*** Subjects are given 8 1 or 2-move practice problems, which are not included in the overall score.
- Stockings of Cambridge is completed in 4 blocks. It starts with the task phase, which is followed by the motor phase. This sequence is then repeated with a slight increase in difficulty. In the first task phase subjects see 2, 3 and 4-move problems twice each. In the second task phase subjects see 4-move problems twice, and 5-move problems four times. There is a total of 12 scored items.

Primary Reference:

Robbins, T.W., James, M., Owen, A.M., Sahakian, B.J., McInnes, L., & Rabbitt, P. (1994). Cambridge Neuropsychological Test Automated Battery (CANTAB): A factor analytic

study of a large sample of normal elderly volunteers. *Dementia, 5*, 266-281. https://doi.org/10.1159/000106735

Software Reference:
CANTAB Eclipse (2007) http://www.cambridgecognition.com/academic/cantabsuite/tests

Task 5.23 Everyday Problem Solving

Description (task duration: approximately 30 minutes):

- Participants are asked to read things taken from things people think are important, such as labels, credit applications and bus schedules, and answer questions based on them.
- This test has 42 questions and is not timed.

Primary Reference:
Willis, S. L., & Marsiske, M. (1993). Manual for the everyday problems test. University Park: Pennsylvania State University.

Reasoning Construct: Key to Names and Data Structure in Data Set

Item Name	Abbreviation	Description	Measurement
Subject Number	S#	Subject identifier	
Age Interval	AgeInterval	Age at Epoch recoded into 3-year intervals	20-100
Sex	Sex	Participant's biological sex.	m = Male<br>f = Female
Race	Race	Race that the participant self-identifies with.	1 = Asian American/ Pacific Islander<br>2 = Black/African American<br>3 = Multiracial<br>4 = Native American<br>5 = White/Caucasian<br>6 = Other<br>7 = Unknown
Ethnicity	Ethnicity	Ethnicity that the participant self-identifies with.	1 = Hispanic/Latin(o/a)<br>0 = Non-Hispanic
Handedness Score	HandednessScore	Average score of participant hand preference while completing various tasks. Higher scores indicate preference for the right hand.	Score Range: 0-4<br><br>0 = Always left<br>1 = Usually left<br>2 = No preference<br>3 = Usually right<br>4 = Always right
Mini-Mental State Exam Total	MMSE	Total # of items answered correctly.	Score Range: 0-30
Cognitive Battery Epoch 1-2 Interval	CogW1toW2	Interval between cognitive testing day 1 for Epochs 1-2.	# of Years
Cognitive Battery Epoch 2-3 Interval	CogW2toW3	Interval between cognitive testing day 1 for Epochs 2-3.	# of Years

Cognitive Battery Epoch 1-3 Interval	CogW1toW3	Interval between cognitive testing day 1 for Epochs 1-3.	# of Years
Take Home Epoch 1-2 Interval	TakeHomeW1toW2	Interval between Take Home for Epochs 1-2.	# of Years
Take Home Epoch 2-3 Interval	TakeHomeW2toW3	Interval between Take Home for Epochs 2-3.	# of Years
Take Home Epoch 1-3 Interval	TakeHomeW1toW3	Interval between Take Home for Epochs 1-3.	# of Years
MRI Epoch 1-2 Interval	MRIW1toW2	Interval between MRI scan for Epochs 1-2.	# of Years
MRI Epoch 2-3 Interval	MRIW2toW3	Interval between MRI scan for Epochs 2-3.	# of Years
MRI Epoch 1-3 Interval	MRIW1toW3	Interval between MRI scan for Epochs 1-3.	# of Years
Amyloid PET Epoch 1-2 Interval	PETAmyW1toW2	Interval between amyloid PET scan for Epochs 1-2.	# of Years
Amyloid PET Epoch 2-3 Interval	PETAmyW2toW3	Interval between amyloid PET scan for Epochs 2-3.	# of Years
Amyloid PET Epoch 1-3 Interval	PETAmyW1toW3	Interval between amyloid PET scan for Epochs 1-3.	# of Years
Highest Level of Education Completed	EduComp5	This is an ordinal measure of participants' self-reported highest level of education completed.	1 = Less than high school graduate<br>2 = High school graduate/GED<br>3 = Some college/trade/ technical/business school<br>4 = Bachelor's degree<br>5 = Some graduate work<br>6 = Master's degree<br>7 = MD/JD/PhD/other advanced degree
Education Estimated Years Capped	EduYrsEstCap5	This is a conversion of the participant's self-reported highest level of education into a capped estimated number of years it would take to reach this highest level of education.<br><br>The "capped" comes into play when someone spend a longer time than usual for a certain degree but did not complete it. In short, someone with a	*11 maximum* = Less than High school<br>*12* = High School<br>*15 maximum* = Some College<br>*16* = Bachelor's degree<br>*20 maximum* = Some Graduate Work<br>*18* = Master's degree<br>*21* = MD/JD/PhD/Advanced degree

		lot of years of education but did not complete a degree will not score higher than someone who did complete the degree.	
Construct Name	ConstructName	Reasoning	
Construct Number	ConstructNumber	Construct 5	
Epoch	Wave	Denotes the data collection Epoch. See individual differences data set for more detail, including testing date intervals.	1 = Epoch 1<br>2 = Epoch 2<br>3 = Epoch 3
Has Data	HasData	1 = Yes, returned for Epoch; 2 = No, did not return for Epoch	
Number of Tasks in Construct	NumTasks	How many tasks make up the reasoning construct	4 Tasks for Reasoning
**Task 20—Ravens Matrices**	**Task20**	1 = Has data<br>2 = Task data partial<br>3 = No task data	
Ravens Accuracy Easy	RavenAccE20	Total number of correct items for first 18 Easy problems divided by 18	Score Range: 0-1
Ravens Accuracy Medium	RavenAccM20	Total number of correct items for first 18 Medium problems divided by 18	Score Range: 0-1
Ravens Accuracy Hard	RavenAccH20	Total number of correct items for first 18 Hard problems divided by 18	Score Range: 0-1
Ravens Accuracy All	RavenAccAll20	Total number of correct items for all 24 problems divided by 24	Score Range: 0-1
Ravens Number Correct	RavenNumCor20	Number of correct responses for all 24 problems	Score Range: 0-24
Ravens Time	RavenTime20	Time subjects needed to complete the task	0-15 minutes
Ravens Number Answered	RavenNumAnswer20	Number of problems answered in 15 minutes	Score Range:<br>0-24
**Task 21—ETS Letter Sets**	**Task21**	1 = Has data<br>2 = Task data partial<br>3 = No task data	
ETS Letter Sets Part 1	EtsLsP1_21	Total number of correct items for the first 15 Sets	Score Range: 0-15
ETS Letter Sets Part 2	EtsLsP2_21	Total number of correct items for the last 15 Sets	Score Range: 0-15

ETS Letter Sets Total	EtsLsTOTAL21	Total number of correct items for the whole task.	Score Range: 0-30
**Task 22—Cantab Stockings of Cambridge**	**Task22**	1 = Has data<br>2 = Task data partial<br>3 = No task data	
Cantab Stocking of Cambridge – Number of Problems solved in Minimum Moves	CantabSOCMinMov22	The number of times upon which the subject has successfully completed a test problem in the minimum possible number of moves.	Score Range: 0-12
Cantab Stocking of Cambridge – Mean 2-move problems	CantabSOCMeanMove2_22	The average number of moves the subject made for 2-move problems	
Cantab Stocking of Cambridge - Mean 3-move problems	CantabSOCMeanMove3_22	The average number of moves the subject made for 3-move problems	
Cantab Stocking of Cambridge – Mean 4-move problems	CantabSOCMeanMove4_22	The average number of moves the subject made for 4-move problems	
Cantab Stocking of Cambridge – Mean 5-move problems	CantabSOCMeanMove5_22	The average number of moves the subject made for 5-move problems	
Cantab Stocking of Cambridge	CantabSOCIntialTime2_22	Average initial thinking time is the difference in time taken to select the first ball for the same 2-move problems in the task phase vs. the motor phase.	
Cantab Stocking of Cambridge	CantabSOCIntialTime3_22	Average initial thinking time is the difference in time taken to select the first ball for the same 3-move problems in the task phase vs. the motor phase.	
Cantab Stocking of Cambridge	CantabSOCIntialTime4_22	Average initial thinking time is the difference in time taken to select the first ball for the same 4-move problems in the	

		task phase vs. the motor phase.	
Cantab Stocking of Cambridge	CantabSOCIntialTime5_22	Average initial thinking time is the difference in time taken to select the first ball for the same 5-move problems in the task phase vs. the motor phase.	
Cantab Stocking of Cambridge	CantabSOCSubsequentTime2_22	The average difference in time between selecting the first ball and completing the 2-move problem for the task vs. motor phase, and then dividing this result by the number of moves made.	
Cantab Stocking of Cambridge	CantabSOCSubsequentTime3_22	The average difference in time between selecting the first ball and completing the 3-move problem for the task vs. motor phase, and then dividing this result by the number of moves made.	
Cantab Stocking of Cambridge	CantabSOCSubsequentTime4_22	The average difference in time between selecting the first ball and completing the 4-move problem for the task vs. motor phase, and then dividing this result by the number of moves made.	
Cantab Stocking of Cambridge	CantabSOCSubsequentTime5_22	The average difference in time between selecting the first ball and completing the 5-move problem for the task vs. motor phase, and then dividing this result by the number of moves made.	
**Task 23—Everyday Problem Solving**	**Task23**	1 = Has data<br>2 = Task data partial<br>3 = No task data	
Everyday Problem Solving	Eps23	Total number correct	Score Range: 0-42

Construct 6: Vocabulary

Definition

This construct measures the breadth of vocabulary known by an individual and is a core measure of crystallized intelligence (Diehl, Willis, and Schaie, 1995). Unlike most cognitive measures, verbal ability has been shown to be greater in older adults relative to the young (Park et al., 2002). A classic paper Horn and Cattell (1967) provides a theoretical conceptualization of verbal ability—in relation to crystallized intelligence—and relevant measures.

References

Diehl, M., Willis, S.L., & Schaie, K.W. (1995). Everyday problem solving in older adults: Observational assessment and cognitive correlates. *Psychology and Aging, 10*, 478-491. https://doi.org/10.1037/0882-7974.10.3.478

Horn, J., & Cattell, R.B. (1967). Age differences in fluid and crystallized intelligence. *Acta Psychologica, 26*, 107-129. https://doi.org/10.1016/0001-6918(67)90011-X

Park, D.C., Lautenschlager, G., Hedden, T., Davidson, N.S., Smith, A.D., & Smith, P.K. (2002). Models of visuospatial and verbal memory across the adult life span. *Psychology and Aging, 17*, 299–320. https://doi.org/10.1037/0882-7974.17.2.299

Sample Sizes by Epoch and Task (subjects with partial data in parentheses)

Assessment	Epoch 1	Epoch 2	Epoch 3
**Educational Testing Service Advanced Vocabulary**	463	324	212
**Shipley Vocabulary**	463	0	212
**CANTAB Graded Naming Task**	464	70	212
**NIH Toolbox Oral Reading Recognition Test**	0	304(304)[a]	207(17)[a]
**NIH Toolbox Picture Vocabulary**	0	302(302)[b]	208(18)[b]

Notes on data completeness:
[a]NIH Toolbox Oral Reading Recognition Test: For Epoch 2, thetas and theta SEs are unavailable and fully-corrected scores are only available for 193 participants. For Epoch 3, age-corrected, percentile, and fully-corrected scores are only available for 190 participants. Use of the uncorrected standardized score is recommended.
[b]NIH Toolbox Picture Vocabulary: For Epoch 2, thetas and theta SEs are unavailable and fully-corrected scores are only available for 192 participants. For Epoch 3, age-corrected, percentile, and fully-corrected scores are only available for 191 participants. Use of the uncorrected standardized score is recommended.

Task 6.24 Educational Testing Service Advanced Vocabulary

- **Description (task duration: self-paced, approximately 10-20 minutes):** This a paper and pencil task. Participants compare a target word with five other words and select the one word that means the same or most nearly the same as the target word. The task is divided into 2 sections. Participants are given 4 minutes per section to select 18 synonyms (or 36 trials total).
- **Scoring:** Participants' scores are penalized for wrong answers; total score equals total number of items correct - .25*(number of items incorrect). Higher scores indicate better vocabulary.

Primary Reference:

Ekstrom, R. B., French, J. W., Harman, H., & Derman, D. (1976). *Kit of factor-referenced cognitive tests* (rev. ed.). Princeton, NJ: Educational Testing Service.

Task 6.25 Shipley Vocabulary

- **Description (task duration: self-paced, approximately 10-20 minutes):** This a paper and pencil task. Participants compare a target word with four other words and select the

one that means the same or most nearly the same as the target word. This task is not timed and there are 40 trials.

- **Scoring:** Final score is the total number of items correct. Higher scores indicate better vocabulary.

Primary Reference:

Zachary, A. & Shipley, W. C. (1986). *Shipley Institute of Living Scale. Revised Manual.* Los Angeles, CA: Western Psychological Services.

Task 6.26 CANTAB Graded Naming Task

- **Description (task duration: approximately 10-15 minutes):** Thirty-line drawings are present on a computer screen, one at a time, with increasing difficulty. Participants must orally identify the exact name of each drawing (e.g., kangaroo, bellows). This task is not timed.
- **Scoring:** Final score is the total number of items correct. Higher scores indicate better vocabulary.

Primary Reference:

Robbins, T.W., et al., (1994). Cambridge Neuropsychological Test Automated Battery (CANTAB): A factor analytic study of a large sample of normal elderly volunteers. *Dementia, 5*, 266-281. https://doi.org/10.1159/000106735

Software Reference:

CANTAB Eclipse. *Cambridge Cognition* (2007). https://www.cambridgecognition.com/cantab/cognitive-tests/graded-naming-test-gnt/

Task 6.27 NIH Toolbox Oral Reading Recognition Test

- **Description (task duration: 3 minutes):** Participants see a series of letters and words presented one at a time on the computer screen and are to give the correct pronunciation for that series of letters or words. Items are presented in order of difficulty; the iPad adjusts the difficulty level of items depending on the participant's performance. The number of items presented will depend on age and performance; for most participants, the measure will last approximately 3 minutes and will contain about 25 items. The iPad will administer each item one by one, in an untimed fashion, until the test is completed. The examiner is responsible for recording whether each response is correct.
- **Scoring:** Participants are given credit for each series of letters or words pronounced correctly. Multiple dependent variables are provided via NIH Toolbox: (1) the *NIH Oral Reading Recognition Task Theta score* represents the overall ability or performance of the participant, (2) the *NIH Oral Reading Recognition Task Standard Error* represents the standard error, (3) the *NIH Oral Reading Recognition Task Uncorrected Standard Score* uses a standard score metric (normative mean=100, SD=15) and compares the participant's score to the entire NIH Toolbox nationally representative normative sample, (4) the *NIH Oral Reading Recognition Task Age-Corrected Standard Score* compares the participant's score to scores of participants of the same age in the NIH Toolbox nationally representative normative sample, (5) the *NIH Oral Reading Recognition Task*

National Percentile (age adjusted) represents the percentage of participants the test-taker scored higher than when being compared to participants of the same age, (6) the *NIH Oral Reading Recognition Task Fully-Corrected T-score* represents the performance of the participant in comparison to the NIH Toolbox nationally representative normative sample, while adjusting for key demographic values.

Primary Reference:

Gershon, Richard C et al. "NIH toolbox for assessment of neurological and behavioral function." *Neurology* vol. 80,11 Suppl 3 (2013): S2-6. doi:10.1212/WNL.0b013e3182872e5f

Software Reference:

NIH Toolbox for the iPad test ver. 2.1 https://nihtoolbox.force.com/s/article/nih-toolbox-scoring-and-interpretation-guide

● ***Note:*** Please note the differences in administration for this task across the three Epochs of data collection. Participants in DLBS Epoch 2 performed the NIH Toolbox Oral Reading Recognition Task on a desktop computer, whereas participants in DLBS Epoch 3 performed the task on an iPad. This change was mandated by developers and standardized scores will differ between the two forms of administration. For additional details, we refer you to the NIH Toolbox website: https://www.healthmeasures.net/explore-measurement-systems/nih-toolbox/obtain-and-administer-measures

Task 6.28 NIH Toolbox Picture Vocabulary

- **Description (task duration: 5 minutes):** Participants are presented with four pictures on the iPad screen and an audio recording saying a word. The participant is instructed to touch the picture that most closely shows the meaning of the word. After the participant makes a choice, another set of pictures automatically appears with the next item and associated audio file. The number of items presented depends on age and performance; for most participants, the measure will last approximately five minutes and will contain about 25 items. The iPad administers each item one by one, in an untimed fashion, until the test is completed.
- **Scoring:** Participants are given credit for each correct pairing of audio recording and picture. Multiple dependent variables are provided via NIH Toolbox: (1) the *NIH Picture Vocabulary Task Theta score* represents the overall ability or performance of the participant, (2) the *NIH Picture Vocabulary Task Standard Error* represents the standard error, (3) the *NIH Picture Vocabulary Task Uncorrected Standard Score* uses a standard score metric (normative mean=100, SD=15) and compares the participant's score to the entire NIH Toolbox nationally representative normative sample, (4) the *NIH Picture Vocabulary Task Age-Corrected Standard Score* compares the participant's score to participants of the same age in the NIH Toolbox nationally representative normative sample, (5) the *NIH Picture Vocabulary Task National Percentile (age adjusted)* represents the percentage of participants the test-taker scored higher than when being compared to participants of the same age, (6) the *NIH Picture Vocabulary Task Fully-Corrected T-score* represents the performance of the participant in comparison to the NIH

Toolbox nationally representative normative sample, while adjusting for key demographic values.

Primary Reference:

Gershon, R. C. et al. "NIH toolbox for assessment of neurological and behavioral function." *Neurology* vol. 80,11 Suppl 3 (2013): S2-6. https://doi.org/10.1212/WNL.0b013e3182872e5f

Software Reference:

NIH Toolbox for the iPad test ver. 2.1 https://nihtoolbox.force.com/s/article/nih-toolbox-scoring-and-interpretation-guide

● ***Note***: Please note the differences in administration for this task across the three Epochs of data collection. Participants in DLBS Epoch 2 performed the NIH Toolbox Picture Vocabulary Task on a desktop computer, whereas participants in DLBS Epoch 3 performed the task on an iPad. This change was mandated by developers and standardized scores will differ between the two forms of administration. For additional details, we refer you to the NIH Toolbox website: https://www.healthmeasures.net/explore-measurement-systems/nih-toolbox/obtain-and-administer-measures

Vocabulary Construct Data Set: Key to Names and Data Structure in Data Set

Item Name	Abbreviation	Description	Measurement
Subject Number	S#	Subject identifier	
Age Interval	AgeInterval	Age at Epoch recoded into 3-year intervals	20-100
Sex	Sex	Participant's biological sex.	m = Male<br>f = Female
Race	Race	Race that the participant self-identifies with.	1 = Asian American/ Pacific Islander<br>2 = Black/African American<br>3 = Multiracial<br>4 = Native American<br>5 = White/Caucasian<br>6 = Other<br>7 = Unknown
Ethnicity	Ethnicity	Ethnicity that the participant self-identifies with.	1 = Hispanic/Latin(o/a)<br>0 = Non-Hispanic
Handedness Score	HandednessScore	Average score of participant hand preference while completing various tasks. Higher scores indicate preference for the right hand.	Score Range: 0-4<br><br>0 = Always left<br>1 = Usually left<br>2 = No preference<br>3 = Usually right<br>4 = Always right
Mini-Mental State Exam Total	MMSE	Total # of items answered correctly.	Score Range: 0-30
Cognitive Battery Epoch 1-2 Interval	CogW1toW2	Interval between cognitive testing day 1 for Epochs 1-2.	# of Years

Cognitive Battery Epoch 2-3 Interval	CogW2toW3	Interval between cognitive testing day 1 for Epochs 2-3.	# of Years
Cognitive Battery Epoch 1-3 Interval	CogW1toW3	Interval between cognitive testing day 1 for Epochs 1-3.	# of Years
Take Home Epoch 1-2 Interval	TakeHomeW1toW2	Interval between Take Home for Epochs 1-2.	# of Years
Take Home Epoch 2-3 Interval	TakeHomeW2toW3	Interval between Take Home for Epochs 2-3.	# of Years
Take Home Epoch 1-3 Interval	TakeHomeW1toW3	Interval between Take Home for Epochs 1-3.	# of Years
MRI Epoch 1-2 Interval	MRIW1toW2	Interval between MRI scan for Epochs 1-2.	# of Years
MRI Epoch 2-3 Interval	MRIW2toW3	Interval between MRI scan for Epochs 2-3.	# of Years
MRI Epoch 1-3 Interval	MRIW1toW3	Interval between MRI scan for Epochs 1-3.	# of Years
Amyloid PET Epoch 1-2 Interval	PETAmyW1toW2	Interval between amyloid PET scan for Epochs 1-2.	# of Years
Amyloid PET Epoch 2-3 Interval	PETAmyW2toW3	Interval between amyloid PET scan for Epochs 2-3.	# of Years
Amyloid PET Epoch 1-3 Interval	PETAmyW1toW3	Interval between amyloid PET scan for Epochs 1-3.	# of Years
Highest Level of Education Completed	EduComp5	This is an ordinal measure of participants' self-reported highest level of education completed.	1 = Less than high school graduate<br>2 = High school graduate/GED<br>3 = Some college/trade/ technical/business school<br>4 = Bachelor's degree<br>5 = Some graduate work<br>6 = Master's degree<br>7 = MD/JD/PhD/other advanced degree
Education Estimated Years Capped	EduYrsEstCap5	This is a conversion of the participant's self-reported highest level of education into a capped estimated number of years it would take to reach this highest level of education.<br><br>The "capped" comes into play when someone spend a longer time than usual for a certain degree but did not complete it. In short, someone with a lot of years of education but did not complete a degree will not	*11 maximum* = Less than High school<br>*12* = High School<br>*15 maximum* = Some College<br>*16* = Bachelor's degree<br>*20 maximum* = Some Graduate Work<br>*18* = Master's degree<br>*21* = MD/JD/PhD/ Advanced degree

		score higher than someone who did complete the degree.	
Construct Name	ConstructName	Vocabulary	
Construct Number	ConstructNumber	Construct 6	
Epoch	Wave	Denotes the data collection Epoch. See individual differences data set for more detail, including testing date intervals.	1 = Epoch 1<br>2 = Epoch 2<br>3 = Epoch 3
Has Data	HasData	Yes = 1<br>No = 2	
Number of Tasks in Construct	NumTasks	How many tasks make up the Vocabulary construct	5 tasks for Vocabulary
**Task 24—ETS Vocabulary**	**Task24**	1 = Has data<br>2 = Task data partial<br>3 = No task data	
ETS Advanced Vocabulary Total	ETSVocab24	Dependent Variable: Total # of items correct - .25*(# of items incorrect)	Score Range: 0-36
**Task 25—Shipley Vocabulary**	**Task25**	1 = Has data<br>2 = Task data partial<br>3 = No task data	
Shipley Vocabulary Total	ShipVocab25	Dependent Variable: Total # of items correct	Score Range: 0-40
**Task 26—CANTAB Graded Naming Task**	**Task26**	1 = Has data<br>2 = Task data partial<br>3 = No task data	
CANTAB Graded Naming Task Total	CantabGnt26	Dependent Variable: Total # of items named correctly	Score Range: 0-30
**Task 27—Oral Reading Recognition Task**	**Task27**	1 = Has data<br>2 = Task data partial<br>3 = No task data	
NIH Oral Reading Recognition Task Theta	NIHOralReadTheta27	Item Response Theory (IRT) is used to score ORRT. A score known as a theta score is calculated for each participant; it represents the relative overall ability or performance of the participant.	Mean = 0,<br>Standard Deviation = 1
NIH Oral Reading Recognition Task Standard Error	NIHOralReadSE27	Standard Error	
NIH Oral Reading Recognition Task Uncorrected Standard Score	NIHOralReadUn27	It compares the performance of the test-taker to those in the entire NIH Toolbox nationally representative normative sample, regardless of age or any other variable.	Normative Mean = 100,<br>Standard Deviation = 15
NIH Oral Reading	NIHOralReadAge27	This score compares the score of the test-taker to those in the	Mean = 100,<br>Standard Deviation = 15

Recognition Task Age-Corrected Standard Score		NIH Toolbox nationally representative normative sample at the same age, where a score of 100 indicates performance that was at the national average for the test-taking participant's age. Age-corrected standard scores were derived for adults (ages 18-85).	
NIH Oral Reading Recognition Task National Percentile (age adjusted)	NIHOralReadPercent27	A Percentile represents the percentage of people nationally above whom the participant's score ranks (the comparison group will be based on whichever normative score is used)	Percentile Rank: 0-100
NIH Oral Reading Recognition Task Fully-Corrected T-score	NIHOralReadFully27	This score compares the score of the test-taker to those in the NIH Toolbox nationally representative normative sample, while adjusting for key demographic variables (education, gender, and race/ethnicity) collected during the NIH Toolbox national norming study.	Mean = 50,<br>Standard Deviation = 10
**Task 28—Picture Vocabulary**	**Task28**	1 = Has data<br>2 = Task data partial<br>3 = No task data	
NIH Picture Vocabulary Task Theta	NIHPicVocabTheta28	Item Response Theory (IRT) is used to score the TPVT. A score known as a theta score is calculated for each participant; it represents the relative overall ability or performance of the participant.	Mean = 0,<br>Standard Deviation = 1
NIH Picture Vocabulary Task Standard Error	NIHPicVocabSE28	Standard Error	
NIH Picture Vocabulary Task Uncorrected Standard Score	NIHPicVocabUn28	It compares the performance of the test-taker to those in the entire NIH Toolbox nationally representative normative sample, regardless of age or any other variable.	Normative Mean = 100,<br>Standard Deviation = 15
NIH Picture Vocabulary Task Age-Corrected Standard Score	NIHPicVocabAge28	This score compares the score of the test-taker to those in the NIH Toolbox nationally representative normative sample at the same age, where a score of 100 indicates performance that was at the national average for the test-taking participant's age. Age-	Mean = 100,<br>Standard Deviation = 15

		corrected standard scores were derived for adults (ages 18-85).	
NIH Picture Vocabulary Task National Percentile (age adjusted)	NIHPicVocabPercent28	A Percentile represents the percentage of people nationally above whom the participant's score ranks (the comparison group will be based on whichever normative score is used)	Percentile Rank: 0-100
NIH Picture Vocabulary Task Fully-Corrected T-score	NIHPicVocabFully28	This score compares the score of the test-taker to those in the NIH Toolbox nationally representative normative sample, while adjusting for key demographic variables (education, gender, and race/ethnicity) collected during the NIH Toolbox national norming study.	Mean = 50,<br>Standard Deviation = 10

Construct 7: Verbal Fluency

Definition

The construct of verbal fluency measures verbal knowledge, but also addresses speed of retrieval from semantic memory and thus, has a speed/working memory component. For this reason, verbal fluency is treated as a separate construct from vocabulary. The task requires participants to generate as many words as possible in 60 seconds relating to a letter or a category.

● ***Caution:*** Please note that there are differences in the administration of the phonemic letter task across Epochs. Also note that the semantic category task was not presented in Epoch 1.

References

Spreen, O., & Benton, A. L. (1977). *Neurosensory Center Comprehensive Examination for Aphasia: Manual of instructions (NCCEA)* (rev. ed.). Victoria, BC: University of Victoria.

Salthouse, T. A. (2019). Trajectories of normal cognitive aging. *Psychology and Aging, 34*(1), 17–24.

Hedden, T., Lautenschlager, G., & Park, D. C. (2005). Contributions of Processing Ability and Knowledge to Verbal Memory Tasks across the Adult Life-Span. *The Quarterly Journal of Experimental Psychology Section A*, *58*(1), 169-190.

Sample Sizes by Epoch and Task (subjects with partial data in parentheses)

Assessment	Epoch 1	Epoch 2	Epoch 3
**Controlled Oral Word Association**	462	331	213
**Controlled Oral Association: Categories**	0	331	213

Task 7.29 Controlled Oral Word Association (FAS)

Description (task duration: 3 minutes): This task assesses the spontaneous production of words under a phonemic search condition. Participants are presented with three blocks of letters (F, A, and S) and are asked to **write down (Epoch 1) or say out loud (Epoch 2 and Epoch 3)** as many words beginning with that specific letter as possible in 60 seconds. Responses are recorded for accuracy and each unique word response receives a score of correct (1) or incorrect (0). Proper nouns and repeated words with a different suffix (e.g., friend, friends, friendly) are counted as incorrect. The variables of interest for this task are the number of correct words produced for the F, A, and S blocks as well as a total score.

● ***Caution:*** In Epoch 1, participants were instructed to write down their responses for 60 seconds. In Epoch 2 and Epoch 3, participants were instructed to orally respond, and responses would be recorded for later scoring and validation.

Primary Reference:

Bechtoldt, H.P., Benton, A.L. & Fogel, M.L. (1962). An application of factor analysis in neuropsychology. *The Psychological Record*, *12*, 147–156.

Task 7.30 Controlled Oral Association: Categories

Description (task duration: 2 minutes): This task is similar to the letter task but assesses the spontaneous production of words under a semantic search condition. Participants are presented with two blocks of categories (animals and vegetables) and asked to verbally respond with all the items they can think of that fit into that specific category in 60 seconds. Responses are recorded for accuracy and each unique word response receives a score of correct (1) or incorrect (0). The variables of interest for this task are the total number of correct words produced for the animal and vegetable blocks as well as the total score. It is important to note that this task was *not administered in Epoch 1*.

Primary Reference:

Bechtoldt, H.P., Benton, A.L. & Fogel, M.L. (1962). An application of factor analysis in neuropsychology. *The Psychological Record*, *12*, 147–156.

Verbal Fluency Data Set: Key to Names and Data Structure in Data Set

Item Name	Abbreviation	Description	Measurement
Subject Number	S#	Subject identifier	
Age Interval	AgeInterval	Age at Epoch recoded into 3-year intervals	20-100
Sex	Sex	Participant's biological sex.	m = Male<br>f = Female
Race	Race	Race that the participant self-identifies with.	1 = Asian American/ Pacific Islander<br>2 = Black/African American<br>3 = Multiracial<br>4 = Native American<br>5 = White/Caucasian<br>6 = Other<br>7 = Unknown
Ethnicity	Ethnicity	Ethnicity that the participant self-identifies with.	1 = Hispanic/Latin(o/a)<br>0 = Non-Hispanic
Handedness Score	HandednessScore	Average score of participant hand preference while completing various tasks. Higher scores indicate preference for the right hand.	Score Range: 0-4<br><br>0 = Always left<br>1 = Usually left<br>2 = No preference<br>3 = Usually right<br>4 = Always right
Mini-Mental State Exam Total	MMSE	Total # of items answered correctly.	Score Range: 0-30

Cognitive Battery Epoch 1-2 Interval	CogW1toW2	Interval between cognitive testing day 1 for Epochs 1-2.	# of Years
Cognitive Battery Epoch 2-3 Interval	CogW2toW3	Interval between cognitive testing day 1 for Epochs 2-3.	# of Years
Cognitive Battery Epoch 1-3 Interval	CogW1toW3	Interval between cognitive testing day 1 for Epochs 1-3.	# of Years
Take Home Epoch 1-2 Interval	TakeHomeW1toW2	Interval between Take Home for Epochs 1-2.	# of Years
Take Home Epoch 2-3 Interval	TakeHomeW2toW3	Interval between Take Home for Epochs 2-3.	# of Years
Take Home Epoch 1-3 Interval	TakeHomeW1toW3	Interval between Take Home for Epochs 1-3.	# of Years
MRI Epoch 1-2 Interval	MRIW1toW2	Interval between MRI scan for Epochs 1-2.	# of Years
MRI Epoch 2-3 Interval	MRIW2toW3	Interval between MRI scan for Epochs 2-3.	# of Years
MRI Epoch 1-3 Interval	MRIW1toW3	Interval between MRI scan for Epochs 1-3.	# of Years
Amyloid PET Epoch 1-2 Interval	PETAmyW1toW2	Interval between amyloid PET scan for Epochs 1-2.	# of Years
Amyloid PET Epoch 2-3 Interval	PETAmyW2toW3	Interval between amyloid PET scan for Epochs 2-3.	# of Years
Amyloid PET Epoch 1-3 Interval	PETAmyW1toW3	Interval between amyloid PET scan for Epochs 1-3.	# of Years
Highest Level of Education Completed	EduComp5	This is an ordinal measure of participants' self-reported highest level of education completed.	1 = Less than high school graduate<br>2 = High school graduate/GED<br>3 = Some college/trade/ technical/business school<br>4 = Bachelor's degree<br>5 = Some graduate work<br>6 = Master's degree<br>7 = MD/JD/PhD/other advanced degree
Education Estimated Years Capped	EduYrsEstCap5	This is a conversion of the participant's self-reported highest level of education into a capped estimated number of years it would take to reach this highest level of education.<br><br>The "capped" comes into play when someone spend a longer time than usual	*11 maximum* = Less than High school<br>*12* = High School<br>*15 maximum* = Some College<br>*16* = Bachelor's degree<br>*20 maximum* = Some Graduate Work<br>*18* = Master's degree<br>*21* = MD/JD/PhD/ Advanced degree

		for a certain degree but did not complete it. In short, someone with a lot of years of education but did not complete a degree will not score higher than someone who did complete the degree.	
Construct Name	ConstructName	Verbal Fluency	
Construct Number	ConstructNumber	Construct 7	
Epoch	Wave	Denotes the data collection Epoch. See individual differences data set for more detail, including testing date intervals.	1 = Epoch 1<br>2 = Epoch 2<br>3 = Epoch 3
Has Data	HasData	1 = Yes, returned for Epoch; 2 = No, did not return for Epoch	
Number of Tasks in Construct	NumTasks	How many tasks make up the Verbal Fluency construct	2 Tasks for Verbal Fluency
**Task 29—Controlled Oral Association Letters**	**Task 29**	1 = Has data<br>2 = Task data partial<br>3 = No task data	
Written Controlled Oral Association Letters *[Epoch 1 Only]*	WContOralAssocF29	Total # correct for words beginning with letter F	DLBS Score Range: 0-24
Written Controlled Oral Association Letters *[Epoch 1 Only]*	WContOralAssocA29	Total # correct for words beginning with letter A	DLBS Score Range: 0-21
Written Controlled Oral Association Letters *[Epoch 1 Only]*	WContOralAssocS29	Total # correct for words beginning with letter S	DLBS Score Range: 0-24
Written Controlled Oral Association Letter *[Epoch 1 Only]*	WContOralAssocLetterTot29	Total # of words correct summed across F, A, and S blocks	DLBS Score Range: 0-66
Controlled Oral Association Letters	ContOralAssocF29	Total # correct for words beginning with letter F	Score Range: 0-28
Controlled Oral Association Letters	ContOralAssocA29	Total # correct for words beginning with letter A	Score Range: 0-29

Controlled Oral Association Letters	ContOralAssocS29	Total # correct for words beginning with letter S	Score Range: 0-32
Controlled Oral Association Letters	ContOralAssocTot29	Total # of words correct summed across F, A, and S blocks	Score Range: 0-81
**Task 30—Controlled Oral Association Categories**	**Task30**	1 = Has data<br>2 = Task data partial<br>3 = No task data	
Controlled Oral Association Categories	ContOralAssocCatAni30	Total # correct for animals produced	Score Range: 0-36
Controlled Oral Association Categories	ContOralAssocCatVeg30	Total # correct for vegetables produced	Score Range: 0-30
Controlled Oral Association	ContOralAssocCatTot30	Total # of words correct summed across animal and vegetable blocks	Score Range: 0-66

Structural Brain Data

The Dallas Lifespan Brain Study

Revised: 2025-06-18

Using the Structural data

The structural data includes all task information, data coding, and data spreadsheets for the MRI structural measures in the Dallas Lifespan Brain Study.

The structural data in the KTTK is parcellated into 4 morphometric parameters which includes cortical thickness, gray matter volume, surface area, and subcortical volume. Additionally, we include summary global measures.

Each of the cortical parameters include regional parcellations conducted independently in the two hemispheres. To access any of the tasks within each construct, select the task of interest. The key to the names and data structure used for data coding of each construct spreadsheet is also included in this document and can be accessed by selecting "Data Coding Sheet" included under each construct listed below. Finally, the spreadsheet for each construct can be found listed below and accessed by selecting "Spreadsheet of data" listed under each construct.

Structural Data
- Structural MRI Data Processing Description
- Data Coding Sheets

Structural MRI Data

Table of Contents

Sample Sizes By Epoch and Task

Section 1: Brief Descriptions of Structural Data Processing

Section 2: Access to Structural Summary Data

Sample Sizes by Epoch and Assessment

Assessment	Epoch 1	Epoch 2	Epoch 3
**Structural Data**	464	295	190

Structural Data Processing Description

For all three Epochs of DLBS data collection, MRI scans were processed cross-sectionally through FreeSurfer ver. 5.3 (Martinos Center for Biomedical Imaging, MA, USA) with regional parcellations based on the Desikan-Killiany atlas (Desikan et al., 2006). Extensively trained operators inspected the reconstructed white and grey matter surfaces and performed manual edits when necessary. Quality control was extensive. A second highly trained and independent group reviewed each parcellation for accuracy. For each region automatically parcellated by FreeSurfer, cortical thickness was computed as the distance between the pial surface and grey-white matter boundary, grey matter volume was computed as the volume between those two boundaries, and cortical surface area was computed as the surface area of the grey-white matter boundary.

References

Desikan, R. S., Ségonne, F., Fischl, B., Quinn, B. T., Dickerson, B. C., Blacker, D., ... & Albert, M. S. (2006). An automated labeling system for subdividing the human cerebral cortex on MRI scans into based regions of interest. *NeuroImage 31*(3), 968–980. https://doi.org/10.1016/j.neuroimage.2006.01.021

Cortical Thickness Data Set: Key to Names and Data Structure in Data Set

Item Name	Abbreviation	Description	Measurement
Subject Number	S#	Subject identifier	
AIRC Number	AIRC_ID	AIRC subject identifier	
Construct Name	ConstructName	Cortical Thickness	
Epoch	Wave	Denotes the data collection Epoch. See individual differences data set	1 = Epoch 1<br>2 = Epoch 2<br>3 = Epoch 3

		for more detail, including testing date intervals.	
Has Data	HasData	1 = Yes, returned for Epoch; 2 = No, did not return for Epoch	
Number of Scores in Construct	NumScores	How many scores are available	68
Cortical Thickness	Thickness	1 = Has data<br>2 = Task data partial<br>3 =No task data	
Left banks of superior temporal	LhBanksstsThick	Thickness for ROI	Score Range: 1-5
Left caudal anterior cingulate	LhCaudalanteriorcingulateThick	Thickness for ROI	Score Range: 1-5
Left caudal middle frontal	LhCaudalmiddlefrontalThick	Thickness for ROI	Score Range: 1-5
Left cuneus	LhCuneusThick	Thickness for ROI	Score Range: 1-5
Left entorhinal	LhEntorhinalThick	Thickness for ROI	Score Range: 1-5
Left fusiform	LhFusiformThick	Thickness for ROI	Score Range: 1-5
Left inferior parietal	LhInferiorparietalThick	Thickness for ROI	Score Range: 1-5
Left inferior temporal	LhInferiortemporalThick	Thickness for ROI	Score Range: 1-5
Left cingulate isthmus	LhIsthmuscingulateThick	Thickness for ROI	Score Range: 1-5
Left lateral occipital	LhLateraloccipitalThick	Thickness for ROI	Score Range: 1-5
Left lateral orbitofrontal	LhLateralorbitofrontalThick	Thickness for ROI	Score Range: 1-5
Left lingual	LhLingualThick	Thickness for ROI	Score Range: 1-5
Left medial orbitofrontal	LhMedialorbitofrontalThick	Thickness for ROI	Score Range: 1-5
Left middle temporal	LhMiddletemporalThick	Thickness for ROI	Score Range: 1-5
Left parahippocampal	LhParahippocampalThick	Thickness for ROI	Score Range: 1-5
Left paracentral	LhParacentralThick	Thickness for ROI	Score Range: 1-5
Left parsopercularis	LhParsopercularisThick	Thickness for ROI	Score Range: 1-5
Left parsorbitalis	LhParsorbitalisThick	Thickness for ROI	Score Range: 1-5
Left pars triangularis	LhParstriangularisThick	Thickness for ROI	Score Range: 1-5
Left pericalcarine	LhPericalcarineThick	Thickness for ROI	Score Range: 1-5
Left postcentral	LhPostcentralThick	Thickness for ROI	Score Range: 1-5
Left posterior cingulate	LhPosteriorcingulateThick	Thickness for ROI	Score Range: 1-5
Left precentral	LhPrecentralThick	Thickness for ROI	Score Range: 1-5
Left precuneus	LhPrecuneusThick	Thickness for ROI	Score Range: 1-5
Left rostral anterior cingulate	LhRostralanteriorcingulateThick	Thickness for ROI	Score Range: 1-5
Left rostral middle frontal	LhRostralmiddlefrontalThick	Thickness for ROI	Score Range: 1-5
Left superior frontal	LhSuperiorfrontalThick	Thickness for ROI	Score Range: 1-5
Left superior parietal	LhSuperiorparietalThick	Thickness for ROI	Score Range: 1-5
Left superior temporal	LhSuperiortemporalThick	Thickness for ROI	Score Range: 1-5
Left supramarginal	LhSupramarginalThick	Thickness for ROI	Score Range: 1-5
Left frontal pole	LhFrontalpoleThick	Thickness for ROI	Score Range: 1-5
Left temporal pole	LhTemporalpoleThick	Thickness for ROI	Score Range: 1-5
Left transverse temporal	LhTransversetemporalThick	Thickness for ROI	Score Range: 1-5
Left insula	LhInsulaThick	Thickness for ROI	Score Range: 1-5
Right banks of superior temporal	RhBanksstsThick	Thickness for ROI	Score Range: 1-5
Right caudal anterior cingulate	RhCaudalanteriorcingulateThick	Thickness for ROI	Score Range: 1-5
Right caudal middle frontal	RhCaudalmiddlefrontalThick	Thickness for ROI	Score Range: 1-5
Right cuneus	RhCuneusThick	Thickness for ROI	Score Range: 1-5
Right entorhinal	RhEntorhinalThick	Thickness for ROI	Score Range: 1-5

Right fusiform	RhFusiformThick	Thickness for ROI	Score Range: 1-5
Right inferior parietal	RhInferiorparietalThick	Thickness for ROI	Score Range: 1-5
Right inferior temporal	RhInferiortemporalThick	Thickness for ROI	Score Range: 1-5
Right cingulate isthmus	RhIsthmuscingulateThick	Thickness for ROI	Score Range: 1-5
Right lateral occipital	RhLateraloccipitalThick	Thickness for ROI	Score Range: 1-5
Right lateral orbitofrontal	RhLateralorbitofrontalThick	Thickness for ROI	Score Range: 1-5
Right lingual	RhLingualThick	Thickness for ROI	Score Range: 1-5
Right medial orbitofrontal	RhMedialorbitofrontalThick	Thickness for ROI	Score Range: 1-5
Right middle temporal	RhMiddletemporalThick	Thickness for ROI	Score Range: 1-5
Right parahippocampal	RhParahippocampalThick	Thickness for ROI	Score Range: 1-5
Right paracentral	RhParacentralThick	Thickness for ROI	Score Range: 1-5
Right parsopercularis	RhParsopercularisThick	Thickness for ROI	Score Range: 1-5
Right parsorbitalis	RhParsorbitalisThick	Thickness for ROI	Score Range: 1-5
Right pars triangularis	RhParstriangularisThick	Thickness for ROI	Score Range: 1-5
Right pericalcarine	RhPericalcarineThick	Thickness for ROI	Score Range: 1-5
Right postcentral	RhPostcentralThick	Thickness for ROI	Score Range: 1-5
Right posterior cingulate	RhPosteriorcingulateThick	Thickness for ROI	Score Range: 1-5
Right precentral	RhPrecentralThick	Thickness for ROI	Score Range: 1-5
Right precuneus	RhPrecuneusThick	Thickness for ROI	Score Range: 1-5
Right rostral anterior cingulate	RhRostralanteriorcingulateThick	Thickness for ROI	Score Range: 1-5
Right rostral middle frontal	RhRostralmiddlefrontalThick	Thickness for ROI	Score Range: 1-5
Right superior frontal	RhSuperiorfrontalThick	Thickness for ROI	Score Range: 1-5
Right superior parietal	RhSuperiorparietalThick	Thickness for ROI	Score Range: 1-5
Right superior temporal	RhSuperiortemporalThick	Thickness for ROI	Score Range: 1-5
Right supramarginal	RhSupramarginalThick	Thickness for ROI	Score Range: 1-5
Right frontal pole	RhFrontalpoleThick	Thickness for ROI	Score Range: 1-5
Right temporal pole	RhTemporalpoleThick	Thickness for ROI	Score Range: 1-5
Right transverse temporal	RhTransversetemporalThick	Thickness for ROI	Score Range: 1-5
Right insula	RhInsulaThick	Thickness for ROI	Score Range: 1-5

Grey Matter Volume Data Set: Key to Names and Data Structure in Data Set

Item Name	Abbreviation	Description	Measurement
Subject Number	S#	Subject identifier	
AIRC Number	AIRC_ID	AIRC subject identifier	
Construct Name	ConstructName	GM Volume	
Epoch	Wave	Denotes the data collection Epoch. See individual differences data set for more detail, including testing date intervals.	1 = Epoch 1<br>2 = Epoch 2<br>3 = Epoch 3
Has Data	HasData	1 = Yes, returned for Epoch; 2 = No, did not return for Epoch	
Number of Scores in Construct	NumScores	How many scores are available	68
Grey Matter Volume	Volume	1 = Has data<br>2 = Task data partial<br>3 =No task data	

Left banks of superior temporal	LhBankssstsVol	Volume for ROI	Score Range: 1-35000
Left caudal anterior cingulate	LhCaudalanteriorcingulateVol	Volume for ROI	Score Range: 1-35000
Left caudal middle frontal	LhCaudalmiddlefrontalVol	Volume for ROI	Score Range: 1-35000
Left cuneus	LhCuneusVol	Volume for ROI	Score Range: 1-35000
Left entorhinal	LhEntorhinalVol	Volume for ROI	Score Range: 1-35000
Left fusiform	LhFusiformVol	Volume for ROI	Score Range: 1-35000
Left inferior parietal	LhInferiorparietalVol	Volume for ROI	Score Range: 1-35000
Left inferior temporal	LhInferiortemporalVol	Volume for ROI	Score Range: 1-35000
Left cingulate isthmus	LhIsthmuscingulateVol	Volume for ROI	Score Range: 1-35000
Left lateral occipital	LhLateraloccipitalVol	Volume for ROI	Score Range: 1-35000
Left lateral orbitofrontal	LhLateralorbitofrontalVol	Volume for ROI	Score Range: 1-35000
Left lingual	LhLingualVol	Volume for ROI	Score Range: 1-35000
Left medial orbitofrontal	LhMedialorbitofrontalVol	Volume for ROI	Score Range: 1-35000
Left middle temporal	LhMiddletemporalVol	Volume for ROI	Score Range: 1-35000
Left parahippocampal	LhParahippocampalVol	Volume for ROI	Score Range: 1-35000
Left paracentral	LhParacentralVol	Volume for ROI	Score Range: 1-35000
Left parsopercularis	LhParsopercularisVol	Volume for ROI	Score Range: 1-35000
Left parsorbitalis	LhParsorbitalisVol	Volume for ROI	Score Range: 1-35000
Left pars triangularis	LhParstriangularisVol	Volume for ROI	Score Range: 1-35000
Left pericalcarine	LhPericalcarineVol	Volume for ROI	Score Range: 1-35000
Left postcentral	LhPostcentralVol	Volume for ROI	Score Range: 1-35000
Left posterior cingulate	LhPosteriorcingulateVol	Volume for ROI	Score Range: 1-35000
Left precentral	LhPrecentralVol	Volume for ROI	Score Range: 1-35000
Left precuneus	LhPrecuneusVol	Volume for ROI	Score Range: 1-35000
Left rostral anterior cingulate	LhRostralanteriorcingulateVol	Volume for ROI	Score Range: 1-35000
Left rostral middle frontal	LhRostralmiddlefrontalVol	Volume for ROI	Score Range: 1-35000
Left superior frontal	LhSuperiorfrontalVol	Volume for ROI	Score Range: 1-35000
Left superior parietal	LhSuperiorparietalVol	Volume for ROI	Score Range: 1-35000
Left superior temporal	LhSuperiortemporalVol	Volume for ROI	Score Range: 1-35000
Left supramarginal	LhSupramarginalVol	Volume for ROI	Score Range: 1-35000
Left frontal pole	LhFrontalpoleVol	Volume for ROI	Score Range: 1-35000
Left temporal pole	LhTemporalpoleVol	Volume for ROI	Score Range: 1-35000
Left transverse temporal	LhTransversetemporalVol	Volume for ROI	Score Range: 1-35000
Left insula	LhInsulaVol	Volume for ROI	Score Range: 1-35000
Right banks of superior temporal	RhBankssstsVol	Volume for ROI	Score Range: 1-35000
Right caudal anterior cingulate	RhCaudalanteriorcingulateVol	Volume for ROI	Score Range: 1-35000
Right caudal middle frontal	RhCaudalmiddlefrontalVol	Volume for ROI	Score Range: 1-35000
Right cuneus	RhCuneusVol	Volume for ROI	Score Range: 1-35000
Right entorhinal	RhEntorhinalVol	Volume for ROI	Score Range: 1-35000
Right fusiform	RhFusiformVol	Volume for ROI	Score Range: 1-35000
Right inferior parietal	RhInferiorparietalVol	Volume for ROI	Score Range: 1-35000
Right inferior temporal	RhInferiortemporalVol	Volume for ROI	Score Range: 1-35000
Right cingulate isthmus	RhIsthmuscingulateVol	Volume for ROI	Score Range: 1-35000
Right lateral occipital	RhLateraloccipitalVol	Volume for ROI	Score Range: 1-35000
Right lateral orbitofrontal	RhLateralorbitofrontalVol	Volume for ROI	Score Range: 1-35000
Right lingual	RhLingualVol	Volume for ROI	Score Range: 1-35000
Right medial orbitofrontal	RhMedialorbitofrontalVol	Volume for ROI	Score Range: 1-35000
Right middle temporal	RhMiddletemporalVol	Volume for ROI	Score Range: 1-35000

Right parahippocampal	RhParahippocampalVol	Volume for ROI	Score Range: 1-35000
Right paracentral	RhParacentralVol	Volume for ROI	Score Range: 1-35000
Right parsopercularis	RhParsopercularisVol	Volume for ROI	Score Range: 1-35000
Right parsorbitalis	RhParsorbitalisVol	Volume for ROI	Score Range: 1-35000
Right pars triangularis	RhParstriangularisVol	Volume for ROI	Score Range: 1-35000
Right pericalcarine	RhPericalcarineVol	Volume for ROI	Score Range: 1-35000
Right postcentral	RhPostcentralVol	Volume for ROI	Score Range: 1-35000
Right posterior cingulate	RhPosteriorcingulateVol	Volume for ROI	Score Range: 1-35000
Right precentral	RhPrecentralVol	Volume for ROI	Score Range: 1-35000
Right precuneus	RhPrecuneusVol	Volume for ROI	Score Range: 1-35000
Right rostral anterior cingulate	RhRostralanteriorcingulateVol	Volume for ROI	Score Range: 1-35000
Right rostral middle frontal	RhRostralmiddlefrontalVol	Volume for ROI	Score Range: 1-35000
Right superior frontal	RhSuperiorfrontalVol	Volume for ROI	Score Range: 1-35000
Right superior parietal	RhSuperiorparietalVol	Volume for ROI	Score Range: 1-35000
Right superior temporal	RhSuperiortemporalVol	Volume for ROI	Score Range: 1-35000
Right supramarginal	RhSupramarginalVol	Volume for ROI	Score Range: 1-35000
Right frontal pole	RhFrontalpoleVol	Volume for ROI	Score Range: 1-35000
Right temporal pole	RhTemporalpoleVol	Volume for ROI	Score Range: 1-35000
Right transverse temporal	RhTransversetemporalVol	Volume for ROI	Score Range: 1-35000
Right insula	RhInsulaVol	Volume for ROI	Score Range: 1-35000

Surface Area Data Set: Key to Names and Data Structure in Data Set

Item Name	Abbreviation	Description	Measurement
Subject Number	S#	Subject identifier	
AIRC Number	AIRC_ID	AIRC subject identifier	
Construct Name	ConstructName	Surface Area	
Epoch	Wave	Denotes the data collection Epoch. See individual differences data set for more detail, including testing date intervals.	1 = Epoch 1<br>2 = Epoch 2<br>3 = Epoch 3
Has Data	HasData	1 = Yes, returned for Epoch; 2 = No, did not return for Epoch	
Number of Scores in Construct	NumScores	How many scores are available	68
Surface Area	Area	1 = Has data<br>2 = Task data partial<br>3 =No task data	
Left banks of superior temporal	LhBanksstsArea	Surface Area for ROI	Score Range: 1-9999
Left caudal anterior cingulate	LhCaudalanteriorcingulateArea	Surface Area for ROI	Score Range: 1-9999
Left caudal middle frontal	LhCaudalmiddlefrontalArea	Surface Area for ROI	Score Range: 1-9999
Left cuneus	LhCuneusArea	Surface Area for ROI	Score Range: 1-9999
Left entorhinal	LhEntorhinalArea	Surface Area for ROI	Score Range: 1-9999
Left fusiform	LhFusiformArea	Surface Area for ROI	Score Range: 1-9999
Left inferior parietal	LhInferiorparietalArea	Surface Area for ROI	Score Range: 1-9999

Left inferior temporal	LhInferiortemporalArea	Surface Area for ROI	Score Range: 1-9999
Left cingulate isthmus	LhIsthmuscingulateArea	Surface Area for ROI	Score Range: 1-9999
Left lateral occipital	LhLateraloccipitalArea	Surface Area for ROI	Score Range: 1-9999
Left lateral orbitofrontal	LhLateralorbitofrontalArea	Surface Area for ROI	Score Range: 1-9999
Left lingual	LhLingualArea	Surface Area for ROI	Score Range: 1-9999
Left medial orbitofrontal	LhMedialorbitofrontalArea	Surface Area for ROI	Score Range: 1-9999
Left middle temporal	LhMiddletemporalArea	Surface Area for ROI	Score Range: 1-9999
Left parahippocampal	LhParahippocampalArea	Surface Area for ROI	Score Range: 1-9999
Left paracentral	LhParacentralArea	Surface Area for ROI	Score Range: 1-9999
Left parsopercularis	LhParsopercularisArea	Surface Area for ROI	Score Range: 1-9999
Left parsorbitalis	LhParsorbitalisArea	Surface Area for ROI	Score Range: 1-9999
Left pars triangularis	LhParstriangularisArea	Surface Area for ROI	Score Range: 1-9999
Left pericalcarine	LhPericalcarineArea	Surface Area for ROI	Score Range: 1-9999
Left postcentral	LhPostcentralArea	Surface Area for ROI	Score Range: 1-9999
Left posterior cingulate	LhPosteriorcingulateArea	Surface Area for ROI	Score Range: 1-9999
Left precentral	LhPrecentralArea	Surface Area for ROI	Score Range: 1-9999
Left precuneus	LhPrecuneusArea	Surface Area for ROI	Score Range: 1-9999
Left rostral anterior cingulate	LhRostralanteriorcingulateArea	Surface Area for ROI	Score Range: 1-9999
Left rostral middle frontal	LhRostralmiddlefrontalArea	Surface Area for ROI	Score Range: 1-9999
Left superior frontal	LhSuperiorfrontalArea	Surface Area for ROI	Score Range: 1-9999
Left superior parietal	LhSuperiorparietalArea	Surface Area for ROI	Score Range: 1-9999
Left superior temporal	LhSuperiortemporalArea	Surface Area for ROI	Score Range: 1-9999
Left supramarginal	LhSupramarginalArea	Surface Area for ROI	Score Range: 1-9999
Left frontal pole	LhFrontalpoleArea	Surface Area for ROI	Score Range: 1-9999
Left temporal pole	LhTemporalpoleArea	Surface Area for ROI	Score Range: 1-9999
Left transverse temporal	LhTransversetemporalArea	Surface Area for ROI	Score Range: 1-9999
Left insula	LhInsulaArea	Surface Area for ROI	Score Range: 1-9999
Right banks of superior temporal	RhBanksstsArea	Surface Area for ROI	Score Range: 1-9999
Right caudal anterior cingulate	RhCaudalanteriorcingulateArea	Surface Area for ROI	Score Range: 1-9999
Right caudal middle frontal	RhCaudalmiddlefrontalArea	Surface Area for ROI	Score Range: 1-9999
Right cuneus	RhCuneusArea	Surface Area for ROI	Score Range: 1-9999
Right entorhinal	RhEntorhinalArea	Surface Area for ROI	Score Range: 1-9999
Right fusiform	RhFusiformArea	Surface Area for ROI	Score Range: 1-9999
Right inferior parietal	RhInferiorparietalArea	Surface Area for ROI	Score Range: 1-9999
Right inferior temporal	RhInferiortemporalArea	Surface Area for ROI	Score Range: 1-9999
Right cingulate isthmus	RhIsthmuscingulateArea	Surface Area for ROI	Score Range: 1-9999
Right lateral occipital	RhLateraloccipitalArea	Surface Area for ROI	Score Range: 1-9999
Right lateral orbitofrontal	RhLateralorbitofrontalArea	Surface Area for ROI	Score Range: 1-9999
Right lingual	RhLingualArea	Surface Area for ROI	Score Range: 1-9999
Right medial orbitofrontal	RhMedialorbitofrontalArea	Surface Area for ROI	Score Range: 1-9999
Right middle temporal	RhMiddletemporalArea	Surface Area for ROI	Score Range: 1-9999
Right parahippocampal	RhParahippocampalArea	Surface Area for ROI	Score Range: 1-9999
Right paracentral	RhParacentralArea	Surface Area for ROI	Score Range: 1-9999
Right parsopercularis	RhParsopercularisArea	Surface Area for ROI	Score Range: 1-9999

Right parsorbitalis	RhParsorbitalisArea	Surface Area for ROI	Score Range: 1-9999
Right pars triangularis	RhParstriangularisArea	Surface Area for ROI	Score Range: 1-9999
Right pericalcarine	RhPericalcarineArea	Surface Area for ROI	Score Range: 1-9999
Right postcentral	RhPostcentralArea	Surface Area for ROI	Score Range: 1-9999
Right posterior cingulate	RhPosteriorcingulateArea	Surface Area for ROI	Score Range: 1-9999
Right precentral	RhPrecentralArea	Surface Area for ROI	Score Range: 1-9999
Right precuneus	RhPrecuneusArea	Surface Area for ROI	Score Range: 1-9999
Right rostral anterior cingulate	RhRostralanteriorcingulateArea	Surface Area for ROI	Score Range: 1-9999
Right rostral middle frontal	RhRostralmiddlefrontalArea	Surface Area for ROI	Score Range: 1-9999
Right superior frontal	RhSuperiorfrontalArea	Surface Area for ROI	Score Range: 1-9999
Right superior parietal	RhSuperiorparietalArea	Surface Area for ROI	Score Range: 1-9999
Right superior temporal	RhSuperiortemporalArea	Surface Area for ROI	Score Range: 1-9999
Right supramarginal	RhSupramarginalArea	Surface Area for ROI	Score Range: 1-9999
Right frontal pole	RhFrontalpoleArea	Surface Area for ROI	Score Range: 1-9999
Right temporal pole	RhTemporalpoleArea	Surface Area for ROI	Score Range: 1-9999
Right transverse temporal	RhTransversetemporalArea	Surface Area for ROI	Score Range: 1-9999
Right insula	RhInsulaArea	Surface Area for ROI	Score Range: 1-9999

Subcortical Volume Data Set: Key to Names and Data Structure in Data Set

Item Name	Abbreviation	Description	Measurement
Subject Number	S#	Subject identifier	
AIRC Number	AIRC_ID	AIRC subject identifier	
Construct Name	ConstructName	Subcortical Volume	From aseg.stats file
Epoch	Wave	Denotes the data collection Epoch. See individual differences data set for more detail, including testing date intervals.	1 = Epoch 1<br>2 = Epoch 2<br>3 = Epoch 3
Has Data	HasData	1 = Yes, returned for Epoch; 2 = No, did not return for Epoch	
Number of Scores in Construct	NumScores	How many scores are available	38
Subcortical Volumes	SubVolumes	1 = Has data<br>2 = Task data partial<br>3 =No task data	
Left-Lateral-Ventricle	LhLatVentVol	Volume for ROI	Score Range: 1-99999
Left-Inf-Lat-Vent	LhInfLatVentVol	Volume for ROI	Score Range: 1-99999
Left-Cerebellum-White-Matter	LhCerebellumWMVol	Volume for ROI	Score Range: 1-99999
Left-Cerebellum-Cortex	LhCerebellumCortexVol	Volume for ROI	Score Range: 1-99999
Left-Thalamus-Proper	LhThalamusProperVol	Volume for ROI	Score Range: 1-99999
Left-Caudate	LhCaudateVol	Volume for ROI	Score Range: 1-99999
Left-Putamen	LhPutamenVol	Volume for ROI	Score Range: 1-99999

Left-Pallidum	LhPallidumVol	Volume for ROI	Score Range: 1-99999
3rd-Ventricle	ThirdVentVol	Volume for ROI	Score Range: 1-99999
4th-Ventricle	FourthVentVol	Volume for ROI	Score Range: 1-99999
Brain-Stem	BrainStemVol	Volume for ROI	Score Range: 1-99999
Left-Hippocampus	LhHippocampusVol	Volume for ROI	Score Range: 1-99999
Left-Amygdala	LhAmygdalaVol	Volume for ROI	Score Range: 1-99999
Left-Accumbens-area	LhAccumbensVol	Volume for ROI	Score Range: 1-99999
Left-VentralDC	LhVentralDCVol	Volume for ROI	Score Range: 1-99999
Left-vessel	LhVesselVol	Volume for ROI	Score Range: 1-99999
Left-choroid-plexus	LhChoroidPVol	Volume for ROI	Score Range: 1-99999
Right-Lateral-Ventricle	RhLatVentVol	Volume for ROI	Score Range: 1-99999
Right-Inf-Lat-Vent	RhInfLatVentVol	Volume for ROI	Score Range: 1-99999
Right-Cerebellum-White-Matter	RhCerebellumWMVol	Volume for ROI	Score Range: 1-99999
Right-Cerebellum-Cortex	RhCerebellumCortexVol	Volume for ROI	Score Range: 1-99999
Right-Thalamus-Proper	RhThalamusProperVol	Volume for ROI	Score Range: 1-99999
Right-Caudate	RhCaudateVol	Volume for ROI	Score Range: 1-99999
Right-Putamen	RhPutamenVol	Volume for ROI	Score Range: 1-99999
Right-Pallidum	RhPallidumVol	Volume for ROI	Score Range: 1-99999
Right-Hippocampus	RhHippocampusVol	Volume for ROI	Score Range: 1-99999
Right-Amygdala	RhAmygdalaVol	Volume for ROI	Score Range: 1-99999
Right-Accumbens-area	RhAccumbensVol	Volume for ROI	Score Range: 1-99999
Right-VentralDC	RhVentralDCVol	Volume for ROI	Score Range: 1-99999
Right-vessel	RhVesselVol	Volume for ROI	Score Range: 1-99999
Right-choroid-plexus	RhChoroidPVol	Volume for ROI	Score Range: 1-99999
5th-Ventricle	FifthVentVol	Volume for ROI	Score Range: 1-99999
Optic-Chiasm	OpticChiasmVol	Volume for ROI	Score Range: 1-99999
CC_Posterior	CCPosteriorVol	Volume for ROI	Score Range: 1-99999
CC_Mid_Posterior	CCMidPosteriorVol	Volume for ROI	Score Range: 1-99999
CC_Central	CCCentralVol	Volume for ROI	Score Range: 1-99999
CC_Mid_Anterior	CCMidAnteriorVol	Volume for ROI	Score Range: 1-99999
CC_Anterior	CCAnteriorVol	Volume for ROI	Score Range: 1-99999

Global Variables Data Set: Key to Names and Data Structure in Data Set

Item Name	Abbreviation	Description	Measurement
Subject Number	S#	Subject identifier	
AIRC Number	AIRC_ID	AIRC subject identifier	
Construct Name	ConstructName	Global Variables	
Epoch	Wave	Denotes the data collection Epoch. See individual differences data set for more detail, including testing date intervals.	1 = Epoch 1<br>2 = Epoch 2<br>3 = Epoch 3
Has Data	HasData	1 = Yes, returned for Epoch; 2 = No, did not return for Epoch	
Number of Scores in Construct	NumScores	How many scores are available	28
Global Variables	Global	1 = Has data<br>2 = Task data partial<br>3 =No task data	

Left hemisphere	LhMeanThick	Mean thickness across all left ROIs	Score Range: 1-5
Right hemisphere	RhMeanThick	Mean thickness across all left ROIs	Score Range: 1-5
Left hemisphere	LhWhiteSurfArea	Total Surface Area across all left ROIs	Score Range: 1-115000
Right hemisphere	RhWhiteSurfArea	Total Surface Area across all right ROIs	Score Range: 1-115000
Estimated Total Intracranial Volume	TotalIntracranialVol	Intracranial Volume	Score Range: 1-2500000
CSF	CSFVol	CSF Volume	Score Range: 1-9999
WM-hypointensities	WMHypointensitiesVol	White Matter Hypointensity Volume	Score Range: 1-99999
non-WM-hypointensities	NonWMHypointensitiesVol	Non-WM Hypointensity Volume	Score Range: 1-99999
BrainSegVol	BrainSegVol	BrainSeg Volume	Score Range: 1-140000
BrainSegVolNotVent	BrainSegVolNotVent	BrainSeg Volume	Score Range: 1-140000
BrainSegVolNotVentSurf	BrainSegVolNotVentSurf	BrainSeg Volume	Score Range: 1-140000
lhCortexVol	LhCortexVol	Left Hemisphere Volume	Score Range: 1-280000
rhCortexVol	RhCortexVol	Right Hemisphere Volume	Score Range: 1-280000
CortexVol	CortexVol	Total Cortex Volume	Score Range: 1-560000
lhCorticalWhiteMatterVol	LhCortWMVol	Left Hemisphere White Matter Volume	Score Range: 1-350000
rhCorticalWhiteMatterVol	RhCortWMVol	Right Hemisphere White Matter Volume	Score Range: 1-350000
CorticalWhiteMatterVol	CortWMVol	Total White Matter Volume	Score Range: 1-650000
SubCortGrayVol	SubCortGMVol	Subcortical Gray Matter Volume	Score Range: 1-75000
TotalGrayVol	TotalGMVol	Total Gray Matter Volume	Score Range: 1-750000
SupraTentorialVol	SupraTentorialVol	SupraTentorial Volume	Score Range: 1-1200000
SupraTentorialVolNotVent	SupraTentorialVolNotVent	SupraTentorial Volume	Score Range: 1-1200000
SupraTentorialVolNotVentVox	SupraTentorialVolNotVentVox	SupraTentorial Volume	Score Range: 1-1200000
MaskVol	MaskVol	Mask Volume	Score Range: 1-2100000
BrainSegVol-to-eTIV	BrainSegVolToeTIV	BrainSeg Volume	Score Range: 0.1-2
MaskVol-to-eTIV	MaskVolToeTIV	Mask Volume	Score Range: 0.1-2
lhSurfaceHoles	LhSurfaceHolesVol	Left Hemisphere Surface Hole Volume	Score Range: 1-300
rhSurfaceHoles	RhSurfaceHolesVol	Right Hemisphere Surface Hole Volume	Score Range: 1-300
SurfaceHoles	SurfaceHolesVol	Total Surface Hole Volume	Score Range: 1-300

Amyloid, Tau, and Genotyping

The Dallas Lifespan Brain Study

Using the Amyloid, Tau and Genotyping data

The amyloid and tau data include all task information, data coding, and data spreadsheets for each of the amyloid and tau constructs in the Dallas Lifespan Brain Study.

The amyloid and tau data in the KTTK is organized by 3 constructs which includes Amyloid, Tau, and Genotyping (APOE, BDNF, COMT, DRD2).

To access any of the constructs, select the construct of interest. The key to the names and data structure used for data coding of each construct spreadsheet is also included in this document and can be accessed by selecting "Data Coding Sheet" included under each construct listed below. Finally, the spreadsheet for each construct can be found listed below and accessed by selecting "Spreadsheet of data" listed under each construct.

Amyloid
- PET Processing Data Description
- Data Coding Sheet

Tau
- PET Processing Data Description
- Data Coding Sheet

Genotyping
- Data Description
- Data Coding Sheet

PET-Amyloid

Table of Contents

Sample Sizes by Epoch and Task

Section 1: Brief Descriptions of PET Data Processing

Section 2: Access to PET Summary Data

Sample Sizes by Epoch and Assessment

Assessment	Epoch 1	Epoch 2	Epoch 3
**PET-Amyloid**	295	180[b]	76[a]

[a]Note: Sample size includes 4 participants who only have Epoch 1 and 3 PET-amyloid data, and 73 who have complete Epoch 1-3 PET-amyloid data.
[b]Note: Eight participants did not have an Epoch 2 MRI, so their PET data were registered to their Epoch 1 MRI. They all had an MRI-PET interval over 3 years (see MRItoAmyloid variable).

PET Data Processing Description

The Dallas Lifespan Brain Study (DLBS) began in 2008 and utilized new in vivo imaging techniques indicative of AD pathology to determine the development process of aging and cognition. One such method scanned for beta-amyloid using the radioligand ^{18}F-AV-45, also known as florbetapir. The corresponding data set includes SUVRs for eight key regions that were averaged to form a global SUVR value and PET counts for a wide range of FreeSurfer regions. The eight regions included: anterior cingulate, posterior cingulate, lateral prefrontal, orbitofrontal, precuneus, lateral parietal, lateral occipital, and lateral temporal cortices.

PET processing: First, PET runs were registered to the first run in the PET sequence to provide motion correction. Second, for each subject with at least two Epochs of data, a mean anatomical template was created using FreeSurfer 5.3's *mri_robust_template* procedure. For subjects with only one Epoch of data, their original T1 was used. Third, the PET data and FreeSurfer parcellations described above were registered to this mean template (or T1) and the relevant PET counts were extracted. Finally, SUVRs were formed using a whole-cerebellum reference.

FreeSurfer processing: For all three Epochs of DLBS data collection, MRI scans were processed cross-sectionally through FreeSurfer ver. 5.3 (Martinos Center for Biomedical Imaging, MA, USA) with regional parcellations based on the Desikan-Killiany atlas (Desikan et al., 2006). Extensively trained operators inspected the reconstructed white and grey matter surfaces and performed manual edits when necessary. Left and right hemisphere parcellations were combined to form bilateral volume of interest masks for the amyloid data.

Amyloid-PET Data Set: Key to Names and Data Structure in Data Set

Item Name	Abbreviation	Description	Measurement
Subject Number	S#	Subject identifier	
Construct Name	ConstructName	Amyloid	
Construct Number	ConstructNumber	Construct 17	
Epoch	Wave	Denotes the data collection Epoch. See individual differences data set for more detail, including testing date intervals.	1 = Epoch 1<br>2 = Epoch 2<br>3 = Epoch 3
Has Data	HasData	1 = Yes, returned for Epoch; 2 = No, did not return for Epoch	
Number of Scores in Construct	NumScores	How many scores are available	59
Amyloid PET	Amyloid	1 = Has data<br>2 = Task data partial<br>3 =No task data	
Interval from MRI to Amyloid scan	MRItoAmyloid	Interval, in years, between MRI and amyloid scan	Score Range: 0-4.5
Caudal anterior cingulate count	CaudalAnteriorCingulateCount	Tracer count for region	Score Range: 1000-50,000
Caudal middle frontal count	CaudalMiddleFrontalCount	Tracer count for region	Score Range: 1000-50,000
Cerebellum cortex count	CerebellumCortexCount	Tracer count for region	Score Range: 1000-50,000
Cerebellum white matter count	CerebellumWhiteMatterCount	Tracer count for region	Score Range: 1000-50,000
Cerebral white matter count	CerebralWhiteMatterCount	Tracer count for region	Score Range: 1000-50,000
Cuneus count	CuneusCount	Tracer count for region	Score Range: 1000-50,000
Entorhinal count	EntorhinalCount	Tracer count for region	Score Range: 1000-50,000
Frontal pole count	FrontalPoleCount	Tracer count for region	Score Range: 1000-50,000
Fusiform count	FusiformCount	Tracer count for region	Score Range: 1000-50,000
Hippocampus count	HippocampusCount	Tracer count for region	Score Range: 1000-50,000
Inferior parietal count	InferiorParietalCount	Tracer count for region	Score Range: 1000-50,000
Inferior temporal count	InferiorTemporalCount	Tracer count for region	Score Range: 1000-50,000
Insula count	InsulaCount	Tracer count for region	Score Range: 1000-50,000
Isthmus cingulate count	IsthmusCingulateCount	Tracer count for region	Score Range: 1000-50,000
Lateral occipital count	LateralOccipitalCount	Tracer count for region	Score Range: 1000-50,000

Lateral orbitofrontal count	LateralOrbitofrontalCount	Tracer count for region	Score Range: 1000-50,000
Lingual count	LingualCount	Tracer count for region	Score Range: 1000-50,000
Medial orbitofrontal count	MedialOrbitofrontalCount	Tracer count for region	Score Range: 1000-50,000
Middle temporal count	MiddleTemporalCount	Tracer count for region	Score Range: 1000-50,000
Negative Mask	NEGMask	Tracer count for precentral, postcentral, and pericalcarine	Score Range: 1000-50,000
Paracentral count	ParacentralCount	Tracer count for region	Score Range: 1000-50,000
Parahippocampal count	ParahippocampalCount	Tracer count for region	Score Range: 1000-50,000
Pars opercularis count	ParsOpercularisCount	Tracer count for region	Score Range: 1000-50,000
Pars orbitalis count	ParsOrbitalisCount	Tracer count for region	Score Range: 1000-50,000
Pars triangularis count	ParsTriangularisCount	Tracer count for region	Score Range: 1000-50,000
Pericalcarine count	PericalcarineCount	Tracer count for region	Score Range: 1000-50,000
Postcentral count	PostcentralCount	Tracer count for region	Score Range: 1000-50,000
Posterior cingulate count	PosteriorCingulateCount	Tracer count for region	Score Range: 1000-50,000
Precentral count	PrecentralCount	Tracer count for region	Score Range: 1000-50,000
Precuneus count	PrecuneusCount	Tracer count for region	Score Range: 1000-50,000
Cerebellum and cerebral white matter count	CerebellumCerebralWMCount	Tracer count for whole cerebellum and cerebral white matter	Score Range: 1000-50,000
Rostral anterior cingulate count	RostralAnteriorCingulateCount	Tracer count for region	Score Range: 1000-50,000
Rostral middle frontal count	RostralMiddleFrontalCount	Tracer count for region	Score Range: 1000-50,000
Superior frontal count	SuperiorFrontalCount	Tracer count for region	Score Range: 1000-50,000
Superior parietal count	SuperiorParietalCount	Tracer count for region	Score Range: 1000-50,000
Superior temporal count	SuperiorTemporalCount	Tracer count for region	Score Range: 1000-50,000
Supramarginal count	SupramarginalCount	Tracer count for region	Score Range: 1000-50,000
Temporal pole count	TemporalPoleCount	Tracer count for region	Score Range: 1000-50,000
Transverse temporal count	TransverseTemporalCount	Tracer count for region	Score Range: 1000-50,000
Combined anterior cingulate VOI count	VOIAnteriorCingulateCount	Count for VOI	Score Range: 1000-50,000

Combined cerebellum VOI count	VOICerebellumCount	Count for VOI	Score Range: 1000-50,000
Combined dorsolateral prefrontal VOI count	VOIDorsolateralPrefrontalCount	Count for VOI	Score Range: 1000-50,000
Combined inferior frontal VOI count	VOIInferiorFrontalCount	Count for VOI	Score Range: 1000-50,000
Combined inferior parietal supramarginal VOI count	VOIInferiorParietalSupramarginalCount	Count for VOI	Score Range: 1000-50,000
Combined lateral parietal VOI count	VOILateralParietalCount	Count for VOI	Score Range: 1000-50,000
Combined lateral temporal VOI count	VOILateralTemporalCount	Count for VOI	Score Range: 1000-50,000
Combined middle frontal VOI count	VOIMiddleFrontalCount	Count for VOI	Score Range: 1000-50,000
Combined orbitofrontal VOI count	VOIOrbitofrontalCount	Count for VOI	Score Range: 1000-50,000
Combined posterior cingulate isthmus VOI count	VOIPosteriorCingulateIsthmusCount	Count for VOI	Score Range: 1000-50,000
Anterior cingulate SUVR	VOIAnteriorCingulateSUVR	Count for VOI / VOICerebellumCount	Score Range: 0.6-2.1
Dorsolateral prefrontal SUVR	VOIDorsolateralPrefrontalSUVR	Count for VOI / VOICerebellumCount	Score Range: 0.6-2.1
Lateral parietal SUVR	VOILateralParietalSUVR	Count for VOI / VOICerebellumCount	Score Range: 0.6-2.1
Lateral temporal SUVR	VOILateralTemporalSUVR	Count for VOI / VOICerebellumCount	Score Range: 0.6-2.1
Orbitofrontal SUVR	VOIOrbitofrontalSUVR	Count for VOI / VOICerebellumCount	Score Range: 0.6-2.1
Posterior cingulate isthmus SUVR	VOIPosteriorCingulateIsthmusSUVR	Count for VOI / VOICerebellumCount	Score Range: 0.6-2.1
Lateral occipital SUVR	LateralOccipitalSUVR	Count for VOI / VOICerebellumCount	Score Range: 0.6-2.1
Precuneus SUVR	PrecuneusSUVR	Count for VOI / VOICerebellumCount	Score Range: 0.6-2.1
Global SUVR	GlobalSUVR	Average SUVR across the eight regions described above	Score Range: 0.6-2.1

PET-Tau

Table of Contents

Sample Sizes By Epoch and Task

Section 1: Brief Descriptions of PET Data Processing

Section 2: Access to PET Summary Data

Sample Sizes by Epoch and Assessment

Assessment	Epoch 2	Epoch 3
**PET-Tau**	60	124[a]

[a]Note: Three participants did not have an Epoch 3 MRI, so their PET data were registered to their Epoch 2 MRI. These all had an MRI-PET interval over 5 years (see MRItoTau variable).

PET Data Processing Description

The Dallas Lifespan Brain Study (DLBS) began in 2008 and utilized new in vivo imaging techniques indicative of AD pathology to determine the development process of aging and cognition. One such method scanned for tau using the radioligand ^{18}F-AV-1451, also known as flortaucipir. The corresponding data set includes standardized uptake ratios (SUVRs) for a temporal meta region presented by Jack et al., 2018, which includes inferior temporal, middle temporal, entorhinal, parahippocampus, fusiform, and amygdala. This region was selected due to its sensitivity in detecting tau accumulation in otherwise healthy aging.

The data set also includes tau SUVRs for the eight regions that we previously used to assess global cortical amyloid (see Construct 17: PET-Amyloid), as well as PET counts for a wide range of FreeSurfer regions. We caution against examining tau in these eight cortical amyloid regions except to compare with deposition of amyloid, as tau does not typically accumulate across the cortex in this widespread manner in cognitively normal participants. The eight global SUVR regions were: anterior cingulate, posterior cingulate, lateral prefrontal, orbitofrontal, precuneus, lateral parietal, lateral occipital, and lateral temporal cortices.

PET processing: First, PET runs were registered to the first run in the PET sequence to provide motion correction. Second, their PET data and FreeSurfer parcellations described above were registered to their most recent MRI scan and the relevant PET counts were extracted. Finally, SUVRs were formed using a whole-cerebellum reference. We note that COVID-19-related research delays resulted in a larger interval between MRI and PET scan for Epoch 3 (see MRItoTau variable). Additionally, PET data were processed cross-sectionally because different PET scanners were used for Epoch 2 and Epoch 3 data.

FreeSurfer processing: For all three Epochs of DLBS data collection, MRI scans were processed cross-sectionally through FreeSurfer ver. 5.3 (Martinos Center for Biomedical Imaging, MA, USA) with regional parcellations based on the Desikan-Killiany atlas (Desikan et al., 2006). Extensively trained operators inspected the reconstructed white and grey matter surfaces and performed manual edits when necessary. Left and right hemisphere parcellations were combined to form bilateral volume of interest masks for the tau data.

Reference

Jack, C. R., Wiste, H. J., Schwarz, C. G., Lowe, V. J., Senjem, M. L., Vemuri, P., ... & Petersen, R. C. (2018). Longitudinal tau PET in ageing and Alzheimer's disease. *Brain, 141,* 1517-1528. https://doi.org/10.1093/brain/awy059

Tau-PET Data Set: Key to Names and Data Structure in Data Set

Item Name	Abbreviation	Description	Measurement
Subject Number	S#	Subject identifier	
Construct Name	ConstructName	Tau	
Construct Number	ConstructNumber	Construct 18	
Epoch	Wave	Denotes the data collection Epoch. See individual differences data set for more detail, including testing date intervals.	1 = Epoch 1<br>2 = Epoch 2<br>3 = Epoch 3
Has Data	HasData	1 = Yes, returned for Epoch; 2 = No, did not return for Epoch	
Number of Scores in Construct	NumScores	How many scores are available	69
Tau PET	Tau	1 = Has data<br>2 = Data partial<br>3 =No task data	
Interval from MRI to Tau scan	MRItoTau	Interval, in years, between MRI and tau scan	Score Range: –0.02 to 6.7
Amygdala count	AmygdalaCount	Tracer count for region	Score Range: 1,000-20,000
Caudal anterior cingulate count	CaudalAnteriorCingulateCount	Tracer count for region	Score Range: 1,000-20,000
Caudal middle frontal count	CaudalMiddleFrontalCount	Tracer count for region	Score Range: 1,000-20,000
Cerebellum cortex count	CerebellumCortexCount	Tracer count for region	Score Range: 1,000-20,000
Cerebellum white matter count	CerebellumWhiteMatterCount	Tracer count for region	Score Range: 1,000-20,000
Cerebral white matter count	CerebralWhiteMatterCount	Tracer count for region	Score Range: 1,000-20,000
Cuneus count	CuneusCount	Tracer count for region	Score Range: 1,000-20,000
Entorhinal count	EntorhinalCount	Tracer count for region	Score Range: 1,000-20,000

Frontal pole count	FrontalPoleCount	Tracer count for region	Score Range: 1,000-20,000
Fusiform count	FusiformCount	Tracer count for region	Score Range: 1,000-20,000
Hippocampus count	HippocampusCount	Tracer count for region	Score Range: 1,000-20,000
Inferior parietal count	InferiorParietalCount	Tracer count for region	Score Range: 1,000-20,000
Inferior temporal count	InferiorTemporalCount	Tracer count for region	Score Range: 1,000-20,000
Insula count	InsulaCount	Tracer count for region	Score Range: 1,000-20,000
Isthmus cingulate count	IsthmusCingulateCount	Tracer count for region	Score Range: 1,000-20,000
Lateral occipital count	LateralOccipitalCount	Tracer count for region	Score Range: 1,000-20,000
Lateral orbitofrontal count	LateralOrbitofrontalCount	Tracer count for region	Score Range: 1,000-20,000
Lingual count	LingualCount	Tracer count for region	Score Range: 1,000-20,000
Medial orbitofrontal count	MedialOrbitofrontalCount	Tracer count for region	Score Range: 1,000-20,000
Middle temporal count	MiddleTemporalCount	Tracer count for region	Score Range: 1,000-20,000
Negative Mask	NEGMask	Tracer count for precentral, postcentral, and pericalcarine	Score Range: 1,000-20,000
Paracentral count	ParacentralCount	Tracer count for region	Score Range: 1,000-20,000
Parahippocampal count	ParahippocampalCount	Tracer count for region	Score Range: 1,000-20,000
Pars opercularis count	ParsOpercularisCount	Tracer count for region	Score Range: 1,000-20,000
Pars orbitalis count	ParsOrbitalisCount	Tracer count for region	Score Range: 1,000-20,000
Pars triangularis count	ParsTriangularisCount	Tracer count for region	Score Range: 1,000-20,000
Pericalcarine count	PericalcarineCount	Tracer count for region	Score Range: 1,000-20,000
Postcentral count	PostcentralCount	Tracer count for region	Score Range: 1,000-20,000
Posterior cingulate count	PosteriorCingulateCount	Tracer count for region	Score Range: 1,000-20,000
Precentral count	PrecentralCount	Tracer count for region	Score Range: 1,000-20,000
Precuneus count	PrecuneusCount	Tracer count for region	Score Range: 1,000-20,000
Cerebellum and cerebral white matter count	CerebellumCerebralWMCount	Tracer count for whole cerebellum and cerebral white matter	Score Range: 1,000-20,000
Rostral anterior cingulate count	RostralAnteriorCingulateCount	Tracer count for region	Score Range: 1,000-20,000

Rostral middle frontal count	RostralMiddleFrontalCount	Tracer count for region	Score Range: 1,000-20,000
Superior frontal count	SuperiorFrontalCount	Tracer count for region	Score Range: 1,000-20,000
Superior parietal count	SuperiorParietalCount	Tracer count for region	Score Range: 1,000-20,000
Superior temporal count	SuperiorTemporalCount	Tracer count for region	Score Range: 1,000-20,000
Supramarginal count	SupramarginalCount	Tracer count for region	Score Range: 1,000-20,000
Temporal pole count	TemporalPoleCount	Tracer count for region	Score Range: 1,000-20,000
Transverse temporal count	TransverseTemporalCount	Tracer count for region	Score Range: 1,000-20,000
Combined anterior cingulate VOI count	VOIAnteriorCingulateCount	Count for VOI	Score Range: 1,000-20,000
Combined cerebellum VOI count	VOICerebellumCount	Count for VOI	Score Range: 1,000-20,000
Combined dorsolateral prefrontal VOI count	VOIDorsolateralPrefrontalCount	Count for VOI	Score Range: 1,000-20,000
Combined inferior frontal VOI count	VOIInferiorFrontalCount	Count for VOI	Score Range: 1,000-20,000
Combined inferior parietal supramarginal VOI count	VOIInferiorParietalSupramarginalCount	Count for VOI	Score Range: 1,000-20,000
Combined lateral parietal VOI count	VOILateralParietalCount	Count for VOI	Score Range: 1,000-20,000
Combined lateral temporal VOI count	VOILateralTemporalCount	Count for VOI	Score Range: 1,000-20,000
Combined middle frontal VOI count	VOIMiddleFrontalCount	Count for VOI	Score Range: 1,000-20,000
Combined orbitofrontal VOI count	VOIOrbitofrontalCount	Count for VOI	Score Range: 1,000-20,000
Combined posterior cingulate isthmus VOI count	VOIPosteriorCingulateIsthmusCount	Count for VOI	Score Range: 1,000-20,000
Anterior cingulate SUVR	VOIAnteriorCingulateSUVR	Count for VOI / VOICerebellumCount	Score Range: 0.7-2.2
Dorsolateral prefrontal SUVR	VOIDorsolateralPrefrontalSUVR	Count for VOI / VOICerebellumCount	Score Range: 0.7-2.2
Lateral parietal SUVR	VOILateralParietalSUVR	Count for VOI / VOICerebellumCount	Score Range: 0.7-2.2
Lateral temporal SUVR	VOILateralTemporalSUVR	Count for VOI / VOICerebellumCount	Score Range: 0.7-2.2
Orbitofrontal SUVR	VOIOrbitofrontalSUVR	Count for VOI / VOICerebellumCount	Score Range: 0.7-2.2
Posterior cingulate isthmus SUVR	VOIPosteriorCingulateIsthmusSUVR	Count for VOI / VOICerebellumCount	Score Range: 0.7-2.2
Lateral occipital SUVR	LateralOccipitalSUVR	Count for VOI / VOICerebellumCount	Score Range: 0.7-2.2
Precuneus SUVR	PrecuneusSUVR	Count for VOI / VOICerebellumCount	Score Range: 0.7-2.2
Global SUVR	GlobalSUVR	Average SUVR across the eight	Score Range: 0.7-2.2

		regions described above	
Inferior temporal SUVR	InferiorTemporalSUVR	Count for VOI / VOICerebellumCount	Score Range: 0.7-2.2
Middle temporal SUVR	MiddleTemporalSUVR	Count for VOI / VOICerebellumCount	Score Range: 0.7-2.2
Superior temporal SUVR	SuperiorTemporalSUVR	Count for VOI / VOICerebellumCount	Score Range: 0.7-2.2
Entorhinal SUVR	EntorhinalSUVR	Count for VOI / VOICerebellumCount	Score Range: 0.7-2.2
Parahippocampus SUVR	ParahippocampalSUVR	Count for VOI / VOICerebellumCount	Score Range: 0.7-2.2
Fusiform SUVR	FusiformSUVR	Count for VOI / VOICerebellumCount	Score Range: 0.7-2.2
Hippocampus SUVR	HippocampusSUVR	Count for VOI / VOICerebellumCount	Score Range: 0.7-2.2
Amygdala SUVR	AmygdalaSUVR	Count for VOI / VOICerebellumCount	Score Range: 0.7-2.2
Temporal meta region SUVR	TemporalMetaSUVR	Average SUVR across the six regions described above	Score Range: 0.7-2.2

Genotyping

Table of Contents

Sample Sizes By Epoch and Task

Section 1: Brief Descriptions of Blood Sample Collection and Inventory, and DNA Isolation and Genotyping

Section 2: Access to Genotyping Summary Data

Sample Sizes by Epoch and Assessment

Assessment	Epoch 1	Epoch 2	Epoch 3
**Genotyping**	419	0	0

Blood Sample Collection and Inventory

Genetics samples were collected via blood for those participants completing PET scanning and via buccal swab (through the mail or in-person) for those participants unable to have a blood draw. All details below were given by Dr. Ramon Diaz-Arrastia during his time at UTSW:

All samples were drawn in tubes with appropriate barcode labeling for accurate identification and tracking according to the protocols below. In order to ensure secure, accurate tracking of all samples once they arrive at UT-Southwestern, Freezerworks Version 5 software program from Dataworks Development, Inc. was used to integrate both the vial labeling and storage tasks into one program, which streamlines data transfer by exchanging data with other programs electronically while ensuring data integrity. The Freezerworks 5.0 program meets regulatory requirements, including 21 CFR Part 11, with a robust audit trail and 45 CFR Part 164 standards for safeguarding of electronic protected health information including administrative and technical safeguards. Freezerworks version 5 contains a basic user identification system to assign three levels of data access: system administrator, data entry, and view only with a unique Username and Password login security maintained by the system administrator to determine the appropriate security level. Further control of unwarranted access to data is provided by encrypting stored information within the database using proprietary methods of the database engine. All necessary training for all study personnel was provided. Upon receipt, all samples were scanned by a barcode reader to confirm receipt and to facilitate subsequent storage and tracking. Each site had appropriate access to the database to maintain consistency and accuracy of the information on samples from their site and to facilitate tracking. A total of 10 mL of whole blood was collected from each participant using a Vacutainer phlebotomy system: 5 mL in a serum separator tube (tiger top) and 5 mL in an EDTA-containing tube (purple top). Blood was centrifuged at (2500 rpm x 10 minutes) within 10 minutes of collection, and serum (from the tiger top tube) was distributed into five 0.5 mL aliquots. Plasma (from the purple top tube) were

distributed into another five 0.5 mL aliquots. Remaining blood cells from the purple top tube were distributed into two 1 mL aliquots. All aliquots were frozen within 30 minutes of collection at -80ºC. Vials containing each aliquot were labeled with barcodes generated by the Freezerworks v. 5 software package. Unused DNA and biological fluids (serum and DNA) were stored at -80ºC the UT Southwestern Alzheimer's Disease Center. These samples are available for future scientifically meritorious studies.

● ***Note:*** Samples are now stored at the Center for Vital Longevity.

DNA Isolation and Genotyping

Data are available for APOE, BDNF, COMT, and DRD2. All details below were given by Dr. Ramon Diaz-Arrastia during his time at UTSW:

Venous blood samples were collected into EDTA-anti-coagulated tubes and genomic DNA was isolated by standard protocols [1]. We routinely obtain 50 – 70 g of DNA from 2 mL of whole blood. Fragments containing each of the polymorphisms were amplified from genomic DNA by polymerase chain reaction (PCR) using Taq DNA polymerase (Roche Diagnostics; Indianapolis, IN) and a thermal profile, reaction conditions and primer sequences optimized for each polymorphism. All amplifications were carried out in an ABI 7900HT thermal cycler (Applied Biosystems, Inc; Foster City, CA). Genotypes were determined by a number of methods, depending upon the nature of the polymorphism. For single nucleotide polymorphisms (SNPs; ApoE, BDNF, COMT, DRD2) genotypes were determined by real-time PCR using TaqMan probes unique for each SNP (Applied Biosystems, Inc; Foster City, CA)(Table 1).

Genomic DNA was extracted from cheek swabs using Qiagen DNA Blood kits (#51162; Qiagen Inc., Valencia, CA, USA). To identify the six APOE genotypes comprising the APOE *E2, *E3 and *E4 alleles, two single nucleotide polymorphisms (SNPs) were assayed using the TaqMan method [Applied Biosystems Inc. (ABI), Foster City, CA, USA]. SNP-specific primers and probes were designed by ABI (TaqMan genotyping assays) and assays were performed according to the manufacturer's instructions in 5 µl total volumes in 384-well plates. The polymorphisms distinguish the *E2 allele from the *E3 and *E4 alleles at amino acid position 158 (NCBI rs7412) and the *E4 allele from the *E2 and *E3 alleles at amino acid position 112 (NCBI rs429358).

Table 1

SNP	Assay ID
ApoE 112	C___3084793_20
ApoE 158	C____904973_10
BDNF	C__11592758_10
COMT	C__25746809_50
DDRD2	C___7486676_10

[1]Miller SA, Dykes DD, Polesky HF: A simple salting out procedure for extracting DNA from human nucleated cells. Nucleic Acids Res 16:1215, 1988

Genotyping Data Set: Key to Names and Data Structure in Data Set

Item Name	Abbreviation	Description	Measurement
Subject Number	S#	Subject identifier	
Construct Name	ConstructName	Genotyping	
Construct Number	ConstructNumber	Construct 19	
Epoch	Wave	Denotes the data collection Epoch. See individual differences data set for more detail, including testing date intervals.	1 = Epoch 1<br>2 = Epoch 2<br>3 = Epoch 3
Has Data	HasData	1 = Yes, returned for Epoch; 2 = No, did not return for Epoch	
Number of Scores in Construct	NumScores	How many scores are available	11
Position 112 polymorphism	APOE112	Polymorphisms distinguishing E2-E4 alleles at amino acid position 112 (NCBI rs429358)	CC to TT
Position 158 polymorphism	APOE158	Polymorphisms distinguishing E2-E4 alleles at amino acid position 158 (NCBI rs7412)	CC to TT
APOE Genotype	APOEGenotype	Specific APOE allele combination	e2/e2 to e4/e4
E4 Allele Carrier Status	E4CarrierStatus	Whether they had at least one e4 allele	1 = yes<br>0 = no
Total APOE4 Alleles	TotalE4Alleles	Total e4 alleles carried	Score Range: 0-2
BDNF Polymorphism	BDNF	BDNF polymorphism	AA, GA, GG
BDNF Genotype	BDNFGenotype	BDNF genotype	Val/Val, Met/Met, Val/Met
COMT Polymorphism	COMT	COMT polymorphism	AA, GA, GG
COMT Genotype	COMTGenotype	COMT genotype	Val/Val, Met/Met, Val/Met
DRD2 Polymorphism	DRD2	DRD2 polymorphism	AA, GA, GG
DRD2 Genotype	DRD2Genotype	DRD2 genotype	Al1/Al1, Al2/Al2, Al1/Al2

Functional MRI Data

The Dallas Lifespan Brain Study

Revised: 2025-06-18

Using the Functional MRI Data

Three in-scanner cognitive tasks were administered to participants during fMRI acquisition at each epoch of data collection. These three tasks – the Subsequent Memory Task, Semantic Judgement Task, and Face/Place Passive Viewing Task – are described below, and are additionally detailed in primary publications cited in each task's respective description. In addition to these three cognitive tasks, all participants also contributed resting state data during each epoch, and also had the opportunity to undergo hypercapnia manipulation at their option to assess cerebrovascular reactivity at each epoch. We note that the subsequent memory tasks were designed to focus on hippocampal and frontal regions; the semantic judgement task focused on frontal parietal regions, and the passive viewing category task was directed primarily towards mental/visual areas.

We provide only raw scans for all of the fMRI tasks described in this section (in the 'func' directory in the OpenNeuro repository, as described in the "How to access these data" section at the beginning of this document).

fMRI Subsequent Memory Task

Description: This task was designed to assess how fMRI BOLD signal during the incidental encoding of images relates to performance in a subsequent recognition test. Using an incidental encoding paradigm, participants were presented with outdoor landscape scenes and had to determine whether there was water present in each scene by pressing a yes or no button. The task consisted of three runs with a random ordering of 96 outdoor scenes (32 scenes per run) presented in an event-related design. Each image was presented for 3s, and intertrial intervals (ITIs) were jittered between 4 and 14 seconds. The total scan time was 20 minutes. Responses were recorded using a fiber-optic button box held in the right hand.

Visual stimuli were presented using E-prime software (Psychology Software Tools, Pittsburgh, PA, USA), projected through the back of the scanner and viewed through a mirror attached to the head coil. Approximately twenty minutes after the encoding trial, an off-line recognition task was administered outside the scanner. A total of 192 pictures were presented, consisting of 96 previously encountered target scenes and 96 closely matched lure scenes. Participants were instructed to make one of three judgements: 1) "high confidence remember" to indicate confident recognition of the exact picture; 2) "low confidence remember" to indicate recognition with low confidence; 3) "new item" to indicate that the picture was not previously presented. The recognition task allowed participants to respond at their own pace with a maximum response time of 4 seconds for each trial.

Primary Reference (Subsequent Memory Task):

Gutchess, A.H., & Park, D.C. (2009). Effects of aging on associative memory for related and unrelated pictures. *European Journal of Cognitive Psychology, 21,* 235-254.

Kennedy, K. M., Rodrigue, K. M., Bischof, G. N., Hebrank, A. C., Reuter-Lorenz, P. A., & Park, D. C. (2015). Age trajectories of functional activation under conditions of low and high processing demands: An adult lifespan fMRI study of the aging brain. *Neuroimage, 104,* 21-34.

fMRI Semantic Judgement Task

Description: This block-design fMRI task was designed to investigate patterns of fMRI BOLD signal during semantic judgements with varying levels of difficulty and ambiguity. The experiment consisted of 8 blocks of easy (non-ambiguous) items and 8 blocks of hard (ambiguous) items, with 8 words per block, drawn from a word bank including 64 ambiguous and 64 non-ambiguous words, respectively. Examples of easy (non-ambiguous) words included "walrus", "truck", and "asphalt". Examples of hard (ambiguous) words included "speaker", "virus", and "sponge". Upon viewing each word, participants made a living or non-living semantic judgment by pressing a button with their right index finger for "yes" (living) or middle finger for "no" (not living). The easy and hard blocks were presented in a pseudo-randomized order, and the words were randomly distributed among the easy and hard condition blocks.

Visual stimuli were presented using E-prime software (Psychology Software Tools, Pittsburgh, PA, USA) projected through the back of the scanner and viewed through a mirror attached to the head coil. During each stimulus presentation, the semantic decision response time was recorded. Each word was displayed for 2500 ms followed by a 500 ms fixation period (crosshair). Additionally, three 24-second fixation blocks were included as a baseline for the scan session. The total scan time was 7.7 min, including a 6-second fixation interval before the first stimulus block. Responses were recorded using a fiber-optic button box held in the right hand.

● ***Note:*** Subject-specific stimulus onset files for the words task were found to be inaccurate. Therefore, a study-wide stimulus onset asynchrony file is provided in this project's root directory (task-Words_run-1_events.tsv).

Primary References (Semantic Judgement Task):

Kennedy, K. M., Rodrigue, K. M., Bischof, G. N., Hebrank, A. C., Reuter-Lorenz, P. A., & Park, D. C. (2015). Age trajectories of functional activation under conditions of low and high processing demands: An adult lifespan fMRI study of the aging brain. *Neuroimage, 104,* 21-34.

Chen, X., Farrell, M. E., Rundle, M. M., Chan, M. Y., Moore, W., Wig, G. S., & Park, D. C. (2021). The relationship of functional hippocampal activity, amyloid deposition, and longitudinal memory decline to memory complaints in cognitively healthy older adults. *Neurobiology of Aging, 105,* 318–326.

fMRI Face/Place Passive Viewing Task ('vv')

Description: This task was designed to assess age-related changes in dedifferentiation, including the neural selectivity to various visual stimuli. Participants viewed images from seven categories: human faces, primate faces, domestic cat faces, wild cat faces, houses, chairs, and phase-scrambled control stimuli. Each category consisted of 64 gray-scale photographs (400 pixels wide x 300 pixels tall), except for domestic and wild cat faces, which had 32 photographs each. The images of human faces were sourced from the face library of Minear and Park[69], and included individuals of different ages, races, and genders. Animal photographs (primate, domestic, and wild cats) were obtained from the internet and cropped to ensure clear visibility of the animal's face. Similar to human faces, only animal images with front-facing views and neutral expressions were selected. Houses were photographed from various locations across the United States, and photographs of chairs were sourced from furniture websites. To create control stimuli, phase-scrambled images were generated by scrambling the phase information in all the

experimental stimuli. This process preserved the spatial frequency information while rendering the visual content meaningless.

The images were presented in 16-second blocks, with each image displayed for 2 seconds. A total of 8 blocks were presented for each category, distributed across two separate runs with four blocks per run. The order of the blocks was randomized for each participant to minimize potential order effects. The stimuli were presented using E-prime software (Psychology Software Tools) and displayed through a back-projection system.

Primary Reference (Face/Place Passive Viewing Task):
Park, D. C., Polk, T. A., Park, R., Minear, M., Savage, A., & Smith, M. R. (2004). Aging reduces neural specialization in ventral visual cortex. *Proceedings of the National Academy of Sciences of the United States of America*, *101*(35), 13091–13095.

fMRI Resting State

Description: In each epoch, participants completed awake resting-state scans, which were meant to allow extraction of "intrinsic" patterns of functional connectivity. During this time, participants were instructed to relax while fixating on a white crosshair against a black background. At the end of the run, the experimenter verified that participants complied with the instructions and did not fall asleep during the functional scan via verbal confirmation. One run of 154 BOLD acquisition was collected in Epoch 1. Two runs of resting-state data were collected in Epochs 2 and 3. The duration of the run was increased from 154 BOLD volumes to 180 volumes part-way through Epoch 2.

Primary Reference (Resting State):
Chan, M. Y., Park, D. C., Savalia, N. K., Petersen, S. E., & Wig, G. S. (2014). Decreased segregation of brain systems across the healthy adult lifespan. *PNAS Proceedings of the National Academy of Sciences of the United States of America, 111(46),* E4997–E5006.

fMRI Hypercapnia

Description. Cerebrovascular reactivity (CVR) was assessed at each epoch via a hypercapnia block-design model. Participants inhaled a 5% CO_2 gas mixture (5% CO_2, 74% N_2, and 21% O_2) while BOLD MR images were simultaneously captured. During the CVR scan, participants wore a nose clip and alternated between breathing room air and the prepared gas (60 seconds CO_2, followed by 60 seconds of room air, repeated three times) through a mouthpiece. Additionally, the concentration of CO_2 in the lungs (end-tidal CO_2 or EtCO2), which closely reflects arterial CO_2 levels, was recorded throughout the breathing task using a capnograph. The total duration of the CVR scan was 7 minutes.

Primary Reference (Hypercapnia):
Lu, H., Xu, F., Rodrigue, K. M., Kennedy, K. M., Chen, Y., Flicker, B., Hebrank, A. C., Uh, J., & Park, D. C. (2011). Alterations in cerebral metabolic rate and blood supply across the adult lifespan. *Cerebral Cortex, 21(6),* 1426–1434.

Health & Psychosocial Data

The Dallas Lifespan Brain Study

Revised: 2025-06-18

Using the Health and Psychosocial data

The health and psychosocial data include all task information, data coding, and data spreadsheets for each of the health and psychosocial constructs in the Dallas Lifespan Brain Study.

The health and psychosocial data in the KTTK is organized by 3 constructs which includes Physical Health, Mental Health and AD screening, and Psychosocial.

Each of the 3 constructs has various tasks associated with it and there are a total of 20 tasks that can be found listed below. To access any of the tasks within each construct, select the task of interest. The key to the names and data structure used for data coding of each construct spreadsheet is also included in this document and can be accessed by selecting "Data Coding Sheet" included under each construct listed below. Finally, the spreadsheet for each construct can be found listed below and accessed by selecting "Spreadsheet of data" listed under each construct.

8 Physical Health
- Task 8.32: Fitness Survey
- Task 8.33: Sf-36
- Task 8.34: Blood Pressure
- Task 8.35: NIH Toolbox Motor Assessment
- Data Coding Sheet

9 Mental Health and AD Screening
- Task 9.36: Geriatric Depression Scale
- Task 9.37: Center for Epidemiological Studies-Depression (CESD)
- Task 9.38: Alzheimer's Disease Assessment Scale—Cognitive Subscale (ADAS-Cog)
- Mental Health and AD Screening Data Set: Key to Names and Data Structure in Data Set
- Mental Health and AD Screening Data Set: Key to Additional Raw Data Available
- Mental Health and AD Screening Data Set: Instruments
- Data Coding Sheet

10 Psychosocial
- Task 10.39: Martin and Park Environmental Demands (MPED) Questionnaire
- Task 10.40: Daily Activities Questionnaire
- Task 10.41: Lifetime Cognitive Activities
- Task 10.42: Need for Cognition Survey (NFC)
- Task 10.43: Metamemory in Adulthood (MIA) Questionnaire
- Task 10.44: Self-Concept Clarity (SCC) Survey
- Task 10.45: Satisfaction with Life Scale
- Task 10.46: Revised Neuroticism-Extraversion-Openness Personality Inventory (NEO-PI-R)
- Task 10.47: Big 5 Inventory

Task 10.48: Personality Survey
Task 10.49: NIH Toolbox Emotion Measures
Task 10.50: Scale of Positive and Negative Experience (SPANE)
Task 10.51: Psychological Well-being (SWQ)
Data Coding Sheet

Construct 8: Physical Health

Table of Contents

Section 1: Brief Descriptions of Questionnaires Presented to Subjects on Health
- Fitness Survey
- SF-36
- Blood Pressure
- NIH Toolbox Motor Assessment

Section 2: Access to Health Questionnaire Summary Data
- Fitness Survey
- SF-36
- Blood Pressure
- NIH Toolbox Motor Assessment

Section 3: Access to Additional Raw Data Available
- Fitness Survey
- SF-36
- Blood Pressure
- NIH Toolbox Motor Assessment

Section 4: Instruments
- Fitness Survey
- SF-36
- Blood Pressure
- NIH Toolbox Motor Assessment

Sample Sizes by Epoch and Assessment (subjects with partial data in parentheses)

Assessment	Epoch 1	Epoch 2	Epoch 3
**Fitness Survey**	460	291	153
**SF-36**	460(7)	289	142
**Blood Pressure**	454(43)[a]	329(51)[a]	213(4)
**NIH Toolbox Motor**	0	301(301)[b]	202(17)[b]

Notes on data completeness:
[a]Blood Pressure: For Epoch 1, 43 participants are missing data for at least one blood pressure assessment, though these missing data are relatively evenly distributed across day and time of assessment. For Epoch 2, only 294 participants have blood pressure data for the end of day 1. Averaging blood pressure across all available assessments is recommended for greater reliability.
[b]NIH Toolbox Motor: For Epoch 2, scores for the non-dominant hand are unavailable, raw scores are only available for 201 participants for pegboard and 197 for grip strength, and fully-corrected scores are only available for 197 for pegboard. Age-corrected, percentile, and fully-corrected scores are unavailable for grip strength. For Epoch 3, pegboard and grip strength scores for the non-dominant hand, age-corrected, percentile, and fully corrected are only available for 186 participants. Use of the uncorrected standardized score of the dominant hand is recommended.

Task Descriptions

Assessment 8.32: Fitness Survey

Description: Participants were asked about their daily fitness activities. This questionnaire has 9 questions concerning the following: exercise, recreation, physical activities other than regular job duties, and daily activities including time spent at work. The first four items were used to develop a summary score, and these items assessed how many times per week the participant performed at least 20 minutes of strenuous exercise (heavy breathing/sweating), at least 30 minutes of non-strenuous exercise, performed muscle strengthening exercises, and how often they watched TV (reverse scored).

Scoring: A summary score, Fitness Total, is provided as an average of the first 4 survey items with the question regarding watching television reverse scored.

Primary Reference:

Revised version based on the physical activities section of the Youth Risk Behavior Surveillance System (YRBSS) 1999. https://www.cdc.gov/healthyyouth/data/yrbs/questionnaires.htm

Kann, L., Kinchen, S. A., Williams, B. I., Ross, J. G., Lowry, R., Grunbaum, J. A., & Kolbe, L. J. (2000). Youth risk behavior surveillance—United States, 1999. *Journal of School Health*, *70*(7), 271-285. https://doi.org/10.1111/j.1746-1561.2000.tb07252.x

- ***Note:*** Please note this is a revised version of the YRBSS.

Assessment 8.33: SF-36

Description: Participants completed 36 questions to measure functional health and well-being. There are 8 health domain scales: Physical Functioning, Role Limitations due to Physical Problems, Bodily Pain, General Health, Vitality, Social Functioning, Role Limitations due to Emotional Problems, and Mental Health.

Scoring: This assessment produces scores from the 8 domains listed above each ranging from 0-100, with higher scores indicating better health. Norm-based scores for the 8 domains are also provided, using the means and standard deviations from the Medical Outcomes Study (N = 2471). Scores were computed manually using the procedure provided by the Rand Corporation.

Primary Reference:

Ware J., Kosinski M., Bjorner J., Turner-Bowker D., Gandek B., & Maruish M. Development. User's Manual for the SF-36v2® Health Survey. Lincoln (RI): QualityMetric Incorporated; 2007.

Scoring Procedure:
https://www.rand.org/health-care/surveys_tools/mos/36-item-short-form/scoring.html

● ***Note:*** Please note whenever researchers publish/report the outcomes for SF36, we must mention that "a modified version of the SF-36v2® was used."

Assessment 8.34: Blood Pressure

Description: Participant's blood pressure (systolic and diastolic) was taken twice at each of the two cognitive sessions, once at each MRI session, and twice at each PET session. The tester assessed the participant's blood pressure and ensured that it was within an expected range. If the participant's blood pressure was outside of the expected range, blood pressure protocol was followed to ensure the participant had access to EMS if needed. Blood pressure data from the two cognitive sessions are available in this data set.

Primary Reference: No official citation.

Assessment 8.35: NIH Toolbox Motor Assessment

Description: Participants completed two tasks to assess their motor skills, the 9-Hole Pegboard Dexterity task and the Grip Strength Task. For the 9-Hole Pegboard Dexterity Task, participants were timed while they used one hand to place pegs in a pegboard one at a time and then removed those same pegs one at a time. First, their dominant hand was tested and then their non-dominant hand was tested. For the Grip Strength Task, participants were asked to squeeze a dynamometer as hard as they could for three seconds. Their grip strength (in lbs.) was recorded for the left and right hands.

Scoring: The pegboard and grip strength tasks include a raw score for both hands—seconds to complete task or pounds of pressure, respectively—as well as scores standardized to the NIH Toolbox nationally-representative normative sample. The standardized scores include an uncorrected score, percentile range, age-corrected score, and a score that is corrected for age, sex, education, and race/ethnicity ("fully-corrected").

Primary Reference:

Gershon, R. C., Wagster, M. V., Hendrie, H. C., Fox, N. A., Cook, K. F., & Nowinsky, C. J. (2013). NIH Toolbox for assessment of neurological and behavioral function. *Neurology*, *80*(11), S1-S92. https://doi.org/10.1212/WNL.0b013e3182872e5f

Software Reference:

NIH Toolbox for the iPad test ver. 2.1. https://nihtoolbox.force.com/s/article/nih-toolbox-scoring-and-interpretation-guide

Physical Health Data Set: Key to Names and Data Structure in Data Set

Item Name	Abbreviation	Description	Measurement
Subject Number	S#	Subject identifier	
Construct Name	ConstructName	Physical Health	
Construct Number	ConstructNumber	Construct 8	
Epoch	Wave	Denotes the data collection Epoch. See individual differences data set for more detail, including testing date intervals.	1 = Epoch 1<br>2 = Epoch 2<br>3 = Epoch 3
Has Data	HasData	1 = Yes, returned for Epoch;<br>2 = No, did not return for Epoch	
Number of Assessments in Data Set	NumAssess	How many assessments make up the physical health data set	4 Assessments for Physical
Assessment 32—Fitness Survey	Assess32	1 = Has data<br>2 = Assessment data partial<br>3 = No data	
Fitness Total	FitTot32	Average of questions 1-4 with Q4 reverse scored	Score Range: 1-7
Assessment 33—SF-36	Assess33	1 = Has data<br>2 = Assessment data partial<br>3 = No data	
Physical Functioning Average Score	SFPhysFuncAvg33	Average of Q3-12 put on a 0-100 scale. A higher score indicates better physical function.	Score Range: 0-100
Role Limitations: Physical Health Problems Average Score	SFLimitPhysAvg33	Average of Q13-16 put on a 0-100 scale. A higher score indicates fewer physical limitations.	Score Range: 0-100
Bodily Pain Average Score	SFBodPainAvg33	Average of Q21, 22 put on a 0-100 scale. A higher score indicates less bodily pain.	Score Range: 0-100

General Health Perceptions Average Score	SFHealthPerceptAvg33	Average of Q1, 33-36 put on a 0-100 scale. A higher score indicates more positive health perceptions.	Score Range: 0-100
Health Change Score	SFHealthChange33	Question 2 put on a 0-100 scale. A higher score indicates better self-reported health compared to one year ago.	Score Range: 0-100
Vitality, Energy, and Fatigue Average Score	SFVitEnerFatAvg33	Average of Q23, 27, 29, 31 put on a 0-100 scale. A higher score indicates better vitality and energy and reduced fatigue.	Score Range: 0-100
Social Functioning Average Score	SFSocFunctAvg33	Average of Q20, 32 put on a 0-100 scale. A higher score indicates better social functioning.	Score Range: 0-100
Role Limitations: Emotional Average Score	SFLimitEmoAvg33	Average of Q18-19 put on a 0-100 scale. A higher score indicates fewer limitations due to emotional problems.	Score Range: 0-100
Mental Health Average Score	SFMentHealAvg33	Average of Q24, 25, 26, 28, 30 put on a 0-100 scale. A higher score indicates better mental health.	Score Range: 0-100
Physical Functioning: Norm-based Score	SFPhysFuncNorm33	Domain score was z-transformed based on norms from the Medical Outcomes Study. Scores above 0 are interpreted as above the US population average and scores below 0 are interpreted as below the US population average.	Score Range: -3-3
Role Limitations: Physical Health Problems Norm-based Score	SFLimitPhysNorm33	Domain score was z-transformed based on norms from the Medical Outcomes Study. Scores above 0 are interpreted as above the US population average and scores below 0 are interpreted as below the US population average.	Score Range: -3-3
Bodily Pain: Norm-based Score	SFBodPainNorm33	Domain score was z-transformed based on norms from the Medical Outcomes Study. Scores above 0 are interpreted as above the US population	Score Range: -3-3

		average and scores below 0 are interpreted as below the US population average.	
General Health Perceptions: Norm-based Score	SFHealthPerceptNorm33	Domain score was z-transformed based on norms from the Medical Outcomes Study. Scores above 0 are interpreted as above the US population average and scores below 0 are interpreted as below the US population average.	Score Range: -3-3
Health Change: Norm-based Score	SFHealthChangeNorm33	Domain score was z-transformed based on norms from the Medical Outcomes Study. Scores above 0 are interpreted as above the US population average and scores below 0 are interpreted as below the US population average.	Score Range: -3-3
Vitality, Energy, and Fatigue: Norm-based Score	SFVitEnerFatNorm33	Domain score was z-transformed based on norms from the Medical Outcomes Study. Scores above 0 are interpreted as above the US population average and scores below 0 are interpreted as below the US population average.	Score Range: -3-3
Social Functioning: Norm-based Score	SFSocFunctNorm33	Domain score was z-transformed based on norms from the Medical Outcomes Study. Scores above 0 are interpreted as above the US population average and scores below 0 are interpreted as below the US population average.	Score Range: -3-3
Role Limitations: Emotional: Norm-based Score	SFLimitEmoNorm33	Domain score was z-transformed based on norms from the Medical Outcomes Study. Scores above 0 are interpreted as above the US population average and scores below 0 are interpreted as below the US population average.	Score Range: -3-3

Mental Health: Norm-based Score	SFMentHealNorm33	Domain score was z-transformed based on norms from the Medical Outcomes Study. Scores above 0 are interpreted as above the US population average and scores below 0 are interpreted as below the US population average.	Score Range: -3-3
Assessment 34—Blood Pressure	Assess34	1 = Has data<br>2 = Assessment data partial<br>3 = No data	
Day 1, Time 1, systolic	BPDay1Time1Sys34	Systolic pressure recorded early on the first day of cognitive testing	Score Range: 73-209
Day 1, Time 1, diastolic	BPDay1Time1Dia34	Diastolic pressure recorded early on the first day of cognitive testing	Score Range: 49-119
Day 1, Time 2, systolic	BPDay1Time2Sys34	Systolic pressure recorded late on the first day of cognitive testing	Score Range: 83-207
Day 1, Time 2, diastolic	BPDay1Time2Dia34	Diastolic pressure recorded late on the first day of cognitive testing	Score Range: 50-134
Day 2, Time 1, systolic	BPDay2Time1Sys34	Systolic pressure recorded early on the second day of cognitive testing	Score Range: 80-211
Day 2, Time 1, diastolic	BPDay2Time1Dia34	Diastolic pressure recorded early on the second day of cognitive testing	Score Range: 51-125
Day 2, Time 2, systolic	BPDay2Time2Sys34	Systolic pressure recorded late on the second day of cognitive testing	Score Range: 83-216
Day 2, Time 2, diastolic	BPDay2Time2Dia34	Diastolic pressure recorded late on the second day of cognitive testing	Score Range: 42-122
Assessment 35—NIH Toolbox 9-Hole Pegboard Dexterity Test	Assess35	1 = Has data<br>2 = Assessment data partial<br>3 = No data	
Dominant hand pegboard raw score	NIHPegDomRaw35	Time, in seconds, to put in and take out all pegs using one's dominant hand.	Score Range: 12-39
Non-dominant hand pegboard raw score	NIHPegNDomRaw35	Time, in seconds, to put in and take out all pegs using one's non-dominant hand.	Score Range: 11-40
Dominant hand pegboard uncorrected standardized score	NIHPegDomUn35	This score compares the score of the test-taker to those in the NIH Toolbox nationally representative normative sample.	Score Range: 59-149

Dominant hand pegboard age-correct score	NIHPegDomAge35	This score compares the score of the test-taker to those in the NIH Toolbox nationally representative normative sample at the same age, where a score of 100 indicates performance that was at the national average for the test-taking participant's age. Age-corrected standard scores were derived for adults (ages 18-85).	Score Range: 65-152
Dominant hand pegboard percentile rank	NIHPegDomPercent35	A Percentile represents the percentage of people nationally above whom the participant's score ranks (the comparison group will be based on whichever normative score is used)	Score Range: 0-100
Dominant hand pegboard fully-corrected score	NIHPegDomFully35	This score compares the score of the test-taker to those in the NIH Toolbox nationally representative normative sample, while adjusting for key demographic variables (age, education, gender, and race/ethnicity) collected during the NIH Toolbox national norming study.	Score Range: 30-152
Non-dominant hand pegboard uncorrected standardized score	NIHPegNDomUn35	This score compares the score of the test-taker to those in the NIH Toolbox nationally representative normative sample.	Score Range: 68-122
Non-dominant hand pegboard age-corrected score	NIHPegNDomAge35	This score compares the score of the test-taker to those in the NIH Toolbox nationally representative normative sample at the same age, where a score of 100 indicates performance that was at the national average for the test-taking participant's age. Age-corrected standard scores were derived for adults (ages 18-85).	Score Range: 72-159
Non-dominant hand pegboard percentile rank	NIHPegNDomPercent35	A Percentile represents the percentage of people	Score Range: 0-100

		nationally above whom the participant's score ranks (the comparison group will be based on whichever normative score is used)	
Non-dominant hand pegboard fully-corrected score	NIHPegNDomFully35	This score compares the score of the test-taker to those in the NIH Toolbox nationally representative normative sample, while adjusting for key demographic variables (age, education, gender, and race/ethnicity) collected during the NIH Toolbox national norming study.	Score Range: 32-86
Dominant hand grip strength raw score	NIHGripDomRaw35	Grip strength, in pounds, when squeezing a dynamometer using one's dominant hand.	Score Range: 9-146
Non-dominant hand grip strength raw score	NIHGripNDomRaw35	Grip strength, in pounds, when squeezing a dynamometer using one's non-dominant hand.	Score Range: 9-146
Dominant hand grip strength uncorrected standardized score	NIHGripDomUn35	This score compares the score of the test-taker to those in the NIH Toolbox nationally representative normative sample.	Score Range: 71-141
Dominant hand grip strength age-corrected score	NIHGripDomAge35	This score compares the score of the test-taker to those in the NIH Toolbox nationally representative normative sample at the same age, where a score of 100 indicates performance that was at the national average for the test-taking participant's age. Age-corrected standard scores were derived for adults (ages 18-85).	Score Range: 55-138
Dominant hand grip strength percentile rank	NIHGripDomPercent35	A Percentile represents the percentage of people nationally above whom the participant's score ranks (the comparison group will be based on whichever normative score is used)	Score Range: 0-100

Dominant hand grip strength fully-corrected score	NIHGripDomFully35	This score compares the score of the test-taker to those in the NIH Toolbox nationally representative normative sample, while adjusting for key demographic variables (age, education, gender, and race/ethnicity) collected during the NIH Toolbox national norming study.	Score Range: -3-73
Non-dominant hand grip strength uncorrected standardized score	NIHGripNDomUn35	This score compares the score of the test-taker to those in the NIH Toolbox nationally representative normative sample.	Score Range: 70-143
Non-dominant hand grip strength age-corrected score	NIHGripNDomAge35	This score compares the score of the test-taker to those in the NIH Toolbox nationally representative normative sample at the same age, where a score of 100 indicates performance that was at the national average for the test-taking participant's age. Age-corrected standard scores were derived for adults (ages 18-85).	Score Range: 49-148
Non-dominant hand grip strength percentile rank	NIHGripNDomPercent35	A Percentile represents the percentage of people nationally above whom the participant's score ranks (the comparison group will be based on whichever normative score is used)	Score Range: 0-100
Non-dominant hand grip strength fully-corrected score	NIHGripNDomFully35	This score compares the score of the test-taker to those in the NIH Toolbox nationally representative normative sample, while adjusting for key demographic variables (age, education, gender, and race/ethnicity) collected during the NIH Toolbox national norming study.	Score Range: -15-83

Physical Health Data Set: Key to Additional Raw Data Available

Item Name	Abbreviation	Description	Measurement
Subject Number	S#	Subject identifier	
Construct Name	ConstructName	Psychosocial	
Construct Number	ConstructNumber	Construct 9	
Epoch	Wave	Denotes the data collection Epoch. See individual differences data set for more detail, including testing date intervals.	1 = Epoch 1<br>2 = Epoch 2<br>3 = Epoch 3
Has Data	HasData	1 = Yes, returned for Epoch;<br>2 = No, did not return for Epoch	
Number of Tasks in Construct	NumTasks	How many tasks make up the physical construct	4 tasks for the physical construct
Assessment 32—Fitness Survey	Assess32	1 = Has data<br>2 = Assessment data partial<br>3 = No data	
20 minute exercise that made you sweat or breathe hard	20Sweat32	During the past year, approximately how many times per week did you exercise or participate in physical activity, that was not part of your job, for at least 20 minutes that made you sweat and breathe hard?	0 = 0 times<br>1 = 1 or 2 times per week<br>2 = 2 or 3 times per week<br>3 = 3 or 4 times per week<br>4 = 4 or 5 times per week<br>5 = 5 or 6 times per week<br>6 = 6 or 7 times per week
30 minute exercise that did not make you sweat or breathe hard	30NoSweat32	During the past year, approximately how many times per week did you participate in physical activity, that was not part of your job, for at least 30 minutes that did not make you sweat or breathe hard?	0 = 0 times<br>1 = 1 or 2 times per week<br>2 = 2 or 3 times per week<br>3 = 3 or 4 times per week<br>4 = 4 or 5 times per week<br>5 = 5 or 6 times per week<br>6 = 6 or 7 times per week
Exercises that strengthened and toned your muscle or increased flexibility	StrengthTrain32	During the past year, approximately how many times per week did you do exercises that were not part of your job, that strengthened and toned	0 = 0 times<br>1 = 1 or 2 times per week<br>2 = 2 or 3 times per week

		your muscles or increased your flexibility?	3 = 3 or 4 times per week<br>4 = 4 or 5 times per week<br>5 = 5 or 6 times per week<br>6 = 6 or 7 times per week
How many hours do you watch TV?	HoursTV32	On an average day, how many hours do you watch TV?	6 = I do not watch TV on an average day.<br>5 = Less than 1 hour per day<br>4 = 1 hour per day<br>3 = 2 hours per day<br>2 = 3 hours per day<br>1 = 4 hours per day<br>0 = 5 or more hours per day
How many hours do you spend in physical activity that makes you sweat and breathe hard?	HoursPhysActSweat32	In a typical day, including time spent at work, how many hours do you spend in physical activity that makes you sweat and breathe hard?	*currently unscored
How many hours do you spend in physical activity that does not make you sweat or breathe hard?	HourPhysActNoSweat32	In a typical day, including time spent at work, how many hours do you spend in physical activity that does not make you sweat or breathe hard?	*currently unscored
How many hours do you spend in sedentary actions that involve no physical effort, such as sitting in front of a computer or spending time on the phone?	HoursSedAct32	In a typical day, including time spent at work, how many hours do you spend in sedentary actions that involve no physical effort, such as sitting in front of a computer or spending time on the phone?	*currently unscored
During the past year, on how many group sports teams did you play?	GroupSports32	During the past year, on how many group sports teams did you play? (Include any teams run by your school, work, religious or community groups.)	*currently unscored
During the past year, in how many sporting contests did you participate in?	SportContests32	During the past year, in how many sporting contests did you participate, such as running, biking or triathlon races, softball, tennis or volleyball tournaments, etc…?	*currently unscored
Assessment 33—SF-36	Assess33	1 = Has data<br>2 = Assessment data partial<br>3 = No data	
General health	SFGenHealth33	In general, would you say your health is:	Score Range: Excellent; Very good; Good; Fair; Poor

How is your health compared to your health a year ago	SFOneYearHealth33	Compared to one year ago, how would you rate your health in general now?	Score Range: Much better now than one year ago; Somewhat better now than one year ago; About the same as one year ago; Somewhat worse now than one year ago; Much worse now than one year ago
Limited in Vigorous activities	SFVigActivities33	Because of your health now, are you limited in Vigorous activities such as running / lifting heavy objects / participating in strenuous sports (like swimming laps)	Score Range: Yes, limited a lot; Yes, limited a little; No, not limited at all
Limited in moderate activities	SFModActivities33	Because of your health now, are you limited in moderate activities such as moving a table / pushing a vacuum cleaner / bowling / or playing golf	Score Range: Yes, limited a lot; Yes, limited a little; No, not limited at all
Limited in carrying groceries	SFCarryGroceries33	Because of your health now, are you limited in lifting or carrying groceries?	Score Range: Yes, limited a lot; Yes, limited a little; No, not limited at all
Limited in climbing several flights of stairs	SFClimbSevStair33	Because of your health now, are you limited in climbing several flights of stairs?	Score Range: Yes, limited a lot; Yes, limited a little; No, not limited at all
Limited in climbing one flight of stairs	SFClimbOneStair33	Because of your health now, are you limited in climbing one flight of stairs?	Score Range: Yes, limited a lot; Yes, limited a little; No, not limited at all
Limited in bending / kneeling / stooping	SFBendStoop33	Because of your health now, are you limited in bending / kneeling / stooping?	Score Range: Yes, limited a lot; Yes, limited a little; No, not limited at all
Limited in walking more than a mile	SFWalkMilePlus33	Because of your health now, are you limited in walking more than a mile?	Score Range: Yes, limited a lot; Yes, limited a little; No, not limited at all
Limited in walking several hundred yards	SFWalkSevHundYard33	Because of your health now, are you limited in walking several hundred yards?	Score Range: Yes, limited a lot; Yes, limited a little; No, not limited at all
Limited in walking one hundred yards	SFWalkHundYard33	Because of your health now, are you limited in walking one hundred yards?	Score Range: Yes, limited a lot; Yes, limited a little; No, not limited at all
Limited in bathing or dressing self	SFBathDress33	Because of your health now, are you limited in bathing or dressing yourself?	Score Range: Yes, limited a lot; Yes,

			limited a little; No, not limited at all
Cut down amount of time spent on work or other activities due to physical health	SFCutDownWork33	During the past four weeks, how much of the time have you had to cut down on the amount of time you spent on work or other activities as a result of your physical health?	Score Range: All of the time; Most of the time; Some of the time; A little of the time; None of the time
Accomplished less than you would like because of physical health	SFAccomplishLess33	During the past four weeks, how much of the time have you accomplished less than you would like as a result of your physical health?	Score Range: All of the time; Most of the time; Some of the time; A little of the time; None of the time
How much time were you limited in the kind of work or other activities due to physical health	SFLimitWorkOthPhys33	During the past four weeks, how much of the time were you limited in the kind of work or other activities you did as a result of your physical health?	Score Range: All of the time; Most of the time; Some of the time; A little of the time; None of the time
Difficulty performing work or other activities due to physical health	SFDiffWorkOthPhys33	During the past four weeks, how much of the time did you have difficulty performing work or other activities (for example/ it took extra effort) as a result of your physical health?	Score Range: All of the time; Most of the time; Some of the time; A little of the time; None of the time
Cut down on amount of time spent work due to emotional problems	SFLimitWorkEmo33	During the past four weeks, how much of the time have you had to cut down on the amount of time you spent on work or other activities as a result of any emotional problems (such as feeling depressed or anxious)?	Score Range: All of the time; Most of the time; Some of the time; A little of the time; None of the time
Accomplished less than you would like due to emotional problems	SFAccomplishLessEmo33	During the past four weeks, how much of the time have you accomplished less than you would like as a result of any emotional problems (such as feeling depressed or anxious)?	Score Range: All of the time; Most of the time; Some of the time; A little of the time; None of the time
Work or do other activities less carefully due to emotional problems	SFLessCareEmo33	During the past four weeks, how much of the time did you do work or other activities less carefully than usual as a result of any emotional problems (such as feeling depressed or anxious)?	Score Range: All of the time; Most of the time; Some of the time; A little of the time; None of the time
To what extent has physical health or emotional problems interfered with social activities	SFLimitedSocialPhysEmo33	During the past four weeks, to what extent has your physical health or emotional problems interfered with your normal social activities with family/friends/neighbors/or groups?	Score Range: Not at all; Slightly; Moderately; Quite a bit; Extremely

Bodily pain in the past four weeks	SFBodPain33	How much bodily pain have you had during the past four weeks?	Score Range: None; Very mild; Mild; Moderate; Severe; Very severe
Pain interference with normal work in the past four weeks	SFPainInterWork33	During the past four weeks, how much did pain interfere with your normal work (including both work outside the home and housework)?	Score Range: Not at all; A little bit; Moderately; Quite a bit; Extremely
Full of life in the past four weeks	SFFullOfLife33	How much of the time during the past four weeks did you feel full of life?	Score Range: All of the time; Most of the time; Some of the time; A little of the time; None of the time
Very nervous in the past four weeks	SFVeryNervous33	How much of the time during the past four weeks have you been very nervous?	Score Range: All of the time; Most of the time; Some of the time; A little of the time; None of the time
Down in the dumps in the past four weeks	SFDownInTheDumps33	How much of the time during the past four weeks have you felt so down in the dumps that nothing could cheer you up?	Score Range: All of the time; Most of the time; Some of the time; A little of the time; None of the time
Calm and peaceful in the past four weeks	SFCalmAndPeace33	How much of the time during the past four weeks have you felt calm and peaceful?	Score Range: All of the time; Most of the time; Some of the time; A little of the time; None of the time
A lot of energy in the past four weeks	SFLotOfEnergy33	How much of the time during the past four weeks did you have a lot of energy?	Score Range: All of the time; Most of the time; Some of the time; A little of the time; None of the time
Downhearted and depressed in the past four weeks	SFDownDepressed33	How much of the time during the past four weeks have you felt downhearted and depressed?	Score Range: All of the time; Most of the time; Some of the time; A little of the time; None of the time
Worn out in the past four weeks	SFWornOut33	How much of the time during the past four weeks did you feel worn out?	Score Range: All of the time; Most of the time; Some of the time; A little of the time; None of the time
Happy in the past four weeks	SFHappy33	How much of the time during the past four weeks have you been happy?	Score Range: All of the time; Most of the time; Some

			of the time; A little of the time; None of the time
Tired in the past four weeks	SFTired33	How much of the time during the past four weeks did you feel tired?	Score Range: All of the time; Most of the time; Some of the time; A little of the time; None of the time
How much of the time	SFTimeLimitedSocialPhysEmo33	During the past four weeks, how much of the time has your physical health or emotional problems interfered with your social activities (like visiting friends/ relatives/ etc.)?	Score Range: All of the time; Most of the time; Some of the time; A little of the time; None of the time
Sick easier than others	SFSickEasier33	I seem to get sick a little easier than other people	Score Range: Definitely true; Mostly true; Don’t know; Mostly false; Definitely false
As healthy as anybody I know	SFHealthAsAny33	I am as healthy as anybody I know	Score Range: Definitely true; Mostly true; Don’t know; Mostly false; Definitely false
Expect health to get worse	SFExpectHeathWorse33	I expect my health to get worse	Score Range: Definitely true; Mostly true; Don’t know; Mostly false; Definitely false
Health is excellent	SFExHealth33	My health is excellent	Score Range: Definitely true; Mostly true; Don’t know; Mostly false; Definitely false
**Assessment 34—Blood Pressure**	Assess34	1 = Has data<br>2 = Assessment data partial<br>3 = No data	
Mean Systolic	BPMeanSys34	Mean of questions 1, 3, 5, & 7	Score Range: 85-200
Mean Diastolic	BPMeanDia34	Mean of questions 2, 4, 6, & 8	Score Range: 56-121

Physical Health Data Set: Instruments

Assessment 8.32 Fitness Survey

The first four questions of this survey are about exercise, recreation, or physical activities other than your regular job duties. For each question, choose the response that best describes your answer.

1. During the past year, approximately how many times per week did you exercise or participate in physical activity, that was not part of your job, for at least 20 minutes that made you sweat and breathe hard?
2. During the past year, approximately how many times per week did you participate in physical activity that was not part of your job, for at least 30 minutes that did not make you sweat or breathe hard?
3. During the past year, approximately how many times per week did you do exercises, that were not part of your job, that strengthened and toned you muscles or increased your flexibility?
4. On an average day, how many hours do you watch TV?

The next questions are about daily activities, including time spent at work. For each question, choose the response that best describes your answer.

5. In a typical day, including time spent at work, how many hours do you spend in physical activity that makes you sweat and breathe hard?
6. In a typical day, including time spent at work, how many hours do you spend in physical activity that does not make you sweat or breathe hard?
7. In a typical day, including time spent at work, how many hours do you spend in sedentary actions that involve no physical effort, such as sitting in front of a computer or spending time on the phone?
8. During the past year, on how many group sports teams did you play? (Include any teams run by your school, work, religious or community groups.)
9. During the past year, in how many sporting contests did you participate, such as running, biking, or triathlon race, softball, tennis or volleyball tournaments, etc…?

Assessment 8.33 SF-36

This survey asks for your views about your health. This information will help keep track of how you feel and how well you are able to do your usual activities. For each question, select the response that best describes your answer.

1. In general, would you say your health is:
2. Compared to one year ago, how would you rate your health in general now?
3. Because of your health now, are you limited in **Vigorous** activities such as running/ lifting heavy objects/ participating in strenuous sports (like swimming laps)?
4. Because of your health now, are you limited in **Moderate** activities such as moving a table/ pushing a vacuum cleaner/ bowling/ or playing golf?
5. Because of your health now, are you limited in lifting or carrying groceries?
6. Because of your health now, are you limited in climbing **several** flights of stairs?
7. Because of your health now, are you limited in climbing **one** flight of stairs?
8. Because of your health now, are you limited in bending/ kneeling/ stooping?

9. Because of your health now, are you limited in walking **more than a mile?**
10. Because of your health now, are you limited in walking **several hundred years?**
11. Because of your health now, are you limited in walking **one hundred yards?**
12. Because of your health now, are you limited in bathing or dressing yourself?
13. During the **past four weeks,** how much of the time have you had to cut down on the amount of time you spent on work or other activities **as a result of your physical health?**
14. During the **past four weeks,** how much of the time have you **accomplished less** than you would like **as a result of your physical health?**
15. During the **past four weeks,** how much of the time were you limited in the **kind** of work or other activities you did **as a result of your physical health?**
16. During the **past four weeks,** how much of the time did you have difficulty performing work or other activities (for example/ it took extra effort) **as a result of your physical health?**
17. During the **past four week,** how much of the time have you had to cut down on the **amount of time** you spent on work or other activities **as a result of any emotional problems** (such as feeling depressed or anxious)?
18. During the **past four weeks,** how much of the time have you **accomplished less** than you would like **as a result of any emotional problems** (such as feeling depressed or anxious)?
19. During the **past four weeks,** how much of the time did you do work or other activities **less carefully than usual as a result of any emotional problems** (such as feeling depressed or anxious)?
20. During the **past four week,** to what extent has your **physical health or emotional problems** interfered with your normal social activities with family/friends/neighbors/or groups?
21. How much **bodily** pain have you had during the **past four weeks?**
22. During the **past four weeks,** how much did **pain** interfere with your normal work (including both work outside the home and housework)?
23. How much of the time during the **past four weeks** did you feel full of life?
24. How much of the time during the **past four weeks** have you been very nervous?
25. How much of the time during the **past four weeks** have you felt so down in the dumps that nothing could cheer you up?
26. How much of the time during the **past four weeks** have you felt calm and peaceful?
27. How much of the time during the **past four weeks** did you have a lot of energy?
28. How much of the time during the **past four weeks** have you felt downhearted and depressed?
29. How much of the time during the **past four weeks** did you feel worn out?
30. How much of the time during the **past four weeks** have you been happy?
31. How much of the time during the **past four weeks** did you feel tired?
32. During the **past four weeks,** how much of the time have your **physical health of emotional problems** interfered with your social activities (like visiting friends/ relatives/ etc.)?

How much would you agree with the following statements?

33. I seem to get sick a little easier than other people.
34. I am as healthy as anybody I know.

35. I expect my health to get worse.
36. My health is excellent.

Assessment 8.34 Blood Pressure

ID:

Blood Pressure Measurement Sheet

Cognitive Battery DAY 1

T1 ________ /________

T2 ________ / ________

Assessment 8.35 NIH Toolbox Motor Assessment

Please refer to publications for questionnaires.

Construct 9: Mental Health and AD Screening

Table of Contents

Sample Sizes By Epoch and Task

Section 1: Brief Descriptions of Questionnaires Presented to Subjects on Mental Health
- Geriatric Depression Scale (GDS)
- Center for Epidemiological Studies- Depression (CESD)
- Alzheimer's Disease Assessment Scale- Cognitive Subscale (ADAS-Cog)

Section 2: Access to Mental Health Questionnaire Summary Data
- Geriatric Depression Scale (GDS)
- Center for Epidemiological Studies- Depression (CESD)
- Alzheimer's Disease Assessment Scale- Cognitive Subscale (ADAS-Cog)

Section 3: Access to Additional Raw Data Available
- Geriatric Depression Scale (GDS)
- Center for Epidemiological Studies-Depression (CESD)
- Alzheimer's Disease Assessment Scale- Cognitive Subscale (ADAS-Cog)

Section 4: Instruments
- Geriatric Depression Scale (GDS)
- Center for Epidemiological Studies- Depression (CESD)
- Alzheimer's Disease Assessment Scale- Cognitive Subscale (ADAS-Cog)

Sample Sizes by Epoch and Assessment (subjects with partial data in parentheses)

Assessment	Epoch 1	Epoch 2	Epoch 3
**GDS**	460	289	145
**CESD**	460	290(1)	147
**ADAS-Cog**	127(15)[a]	331(1)	213

[a]ADAS-Cog: For Epoch 1, only 112 participants have data for the recall and "rest of cognition" portions of the ADAS-Cog.

Task Descriptions

Assessment 9.36 Geriatric Depression Scale

Description: Participants responded to 21 questions regarding their views about their moods to assess symptoms of depression. Note the original questionnaire has 30 questions and our version only includes the first 21.

Scoring: A higher total score represents greater depressive symptoms. This total is determined by reverse scoring and then summing items 1, 5, 7, 9, 15, 19, and 21.

Primary Reference:

Yesavage, J.A., Brink, T.L., Rose, T.L., Lum, O., Huang, V., Adey, M., & Leirer, V.O. (1982). Development and validation of a geriatric depression screening scale: a preliminary report. *J. Psychiatr. Res. 17*(1), 37–49. https://doi.org/10.1016/0022-3956(82)90033-4

● ***Note:*** Please note this is not the official 30 or 15 item scale and cannot be used to classify depressed participants.

Assessment 9.37 Center for Epidemiological Studies-Depression (CESD)

Description: Participants were asked how they felt or behaved during the past week to assess symptoms of depression. This questionnaire has 20 questions.

Scoring: The total scores range from 0 to 60, with higher scores indicating more symptoms of depression. This total is determined by reverse scoring and then summing items 4, 8, 12, and 16.

Primary Reference:

Radloff, L. (1977). A self-report depression scale for research in the general population. *Appl. Psychol. Meas. 1*(3), 385-401. https://doi.org/10.1177/014662167700100306

Assessment 9.38 Alzheimer's Disease Assessment Scale—Cognitive Subscale (ADAS-Cog)

Description: ADAS-Cog is a rating of the severity of cognitive symptoms of Alzheimer's Disease. This assessment includes 11 tasks that assess the cognitive domains of language, memory, and praxis (ability to follow instructions). These tasks include:

- *Word Recall:* The participant read aloud a list of 10 words printed on white cards and was then asked immediately to verbally recall as many of those words as they could. This was done for three trials.
- *Naming Objects and Fingers: This* task required participants to name the fingers of their dominant hand and twelve objects: flower, bed, whistle, pencil, rattle, mask, scissors, comb, wallet, harmonica, stethoscope, and tongs.
- *Commands:* This task asked the participant to perform 5 tasks, each ranging from 1-5 steps.
- *Constructional Praxis:* The test administrator showed the participant four geometric shapes (a circle, two overlapping rectangles, a rhombus, and a cube) and participants were asked to copy those shapes onto a separate sheet of paper.
- *Ideational Praxis:* The participant was told to pretend to send themselves a letter. The instructions they were asked to follow are: fold a letter, put letter in envelope, seal envelope, address envelope, indicate where a stamp goes.
- *Orientation:* The participant was asked to give the following information: their full name, the month, the date, the year, the day, the season, the place, and the time of day.
- *Word Recognition:* The participant read 12 words aloud from the white paper cards, and then viewed a new list with those 12 old words intermixed with 12 new words. For this new list of 24 words, the participant was asked to identify which items they had previously seen. This was conducted for three trials.

- *Language:* The participant's language ability was assessed by the test administrator throughout the completion of the previous tasks.
- *Comprehension of Spoken Language:* The participant's comprehension of spoken language was assessed by the administrator during the previous tasks and assessed how well a participant understood speech.
- *Word Finding Difficulty:* The participant's word finding ability was assessed by the administrator during the previous tasks and showed if/ how much difficulty a participant had finding desired words.
- *Remembering Test Instructions:* The participant's ability to remember test instructions was assessed by the administrator and showed how many times the participant had to be reminded of instructions throughout the administration of the previous tasks.

Scoring: The three Word Recall trials, Object Naming, Commands, Construction Praxis, Ideation Praxis, Orientation, and Word Recognition are reverse scored. These measures are summarized by a recall, rest of cognition, impairment, and total score, as described below in the dataset key.

Primary Reference:

Rosen W. G., Mohs R. C., & Davis K. L (1984). A new rating scale for Alzheimer's disease. *Am J Psychiatry, 141*(11):1356–1364. https://doi.org/10.1176/ajp.141.11.1356

Mental Health and AD Screening Data Set: Key to Names and Data Structure in Data Set

Item Name	Abbreviation	Description	Measurement
Subject Number	S#	Subject identifier	
Construct Name	ConstructName	Mental Health and AD Screening	
Construct Number	ConstructNumber	Construct 9	
Epoch	Wave	Denotes the data collection Epoch. See individual differences data set for more detail, including testing date intervals.	1 = Epoch 1<br>2 = Epoch 2<br>3 = Epoch 3
Has Data	HasData	1 = Yes, returned for Epoch; 2 = No, did not return for Epoch	
Number of Assessments in Data Set	NumAssess	How many assessments make up the Mental Health data set	3 Assessments for Mental Health
Assessment 36—Geriatric Depression Scale	Assess36	1 = Has data<br>2 = Assessment data partial<br>3 = No data	
Geriatric Depression Scale Total	GDSTot36	Sum of the 21 questions, yes = 1, no = 0. Items 1, 5, 7, 9, 15,	Score Range: 0-21

		19, and 21 are reverse scored. A higher score indicates greater depressive symptoms.	
Assessment 37—Center for Epidemiological Studies-Depression	Assess37	1 = Has data<br>2 = Assessment data partial<br>3 = No data	
Center for Epidemiological Studies-Depression Total	CESDTot37	Sum of the 20 questions. Items 4, 8, 12, 16 are reverse scored. A higher score indicates greater depressive symptoms.	Score Range: 0-60
Center for Epidemiological Studies-Depression Answered	CESDAnswered37	Total number of questions answered	Score Range: 0-20
Assessment 38—Alzheimer's Disease Assessment Scale (ADAS)-Cognitive Subscale	Assess38	1 = Has data<br>2 = Assessment data partial<br>3 = No data	
ADAS Word Recall	ADASRcll38	Mean number of words not recalled during the three trials	Score Range: 0-10
ADAS Rest of Task	ADASRestOfCog38	Sum of Naming Object, Commands, Constructional Praxis, Ideational Praxis, Orientation, and Word Recognition (questions 4-9)	Score Range: 0-40
ADAS Impairment	ADASImpair38	Sum of language impairment, comprehension of spoken language, level of word finding difficulty, and remembering test instruction (questions 10-13)	Score Range: 0-20
ADAS Total	ADASTot38	Sum of Mean Word Recall, Rest of Cog, and Impairment. Higher scores indicate greater impairment.	Score Range: 0-70

Mental Health and AD Screening Data Set: Key to Additional Raw Data Available

Item Name	Abbreviation	Description	Measurement
Subject Number	S#	Subject identifier	
Construct Name	ConstructName	Mental Health and AD Screening	
Construct Number	ConstructNumber	Construct 9	
Epoch	Wave	Denotes which Epoch the data were collected. See individual differences data set for more detail.	1 = Epoch 1<br>2 = Epoch 2<br>3 = Epoch 3
Has Data	HasData	1 = Yes, returned for Epoch; 2 = No, did not return for Epoch	
Number of Tasks in Construct	NumTasks	How many tasks make up the Mental Health construct	3 tasks for Mental Health
Assessment 36—Geriatric Depression Scale	Assess36	1 = Has data<br>2 = Assessment data partial<br>3 = No data	
Basically satisfied with life	LifeSatisfact36	Are you basically satisfied with your life?	Score Range: Yes; No
Dropped activities or interests	DropActInt36	Have you dropped many of your activities or interests?	Score Range: Yes; No
Feeling that life is empty	LifeEmpty36	Do you feel that your life is empty?	Score Range: Yes; No
Bored often	Bored36	Do you often get bored?	Score Range: Yes; No
Hopeful about the future	HopeFut36	Are you hopeful about the future?	Score Range: Yes; No
Bothered by thoughts	BotherThoughts36	Are you bothered by thoughts you can't get out of your head?	Score Range: Yes; No
In good spirits	GoodSpirit36	Are you in good spirits most of the time?	Score Range: Yes; No
Afraid that something bad is going to happen	SomethingBad36	Are you afraid that something bad is going to happen to you?	Score Range: Yes; No
Feel happy	Happy36	Do you feel happy most of the time?	Score Range: Yes; No
Feel helpless	Helpless36	Do you often feel helpless?	Score Range: Yes; No
Restless and Fidgety	RestlessFidget36	Do you often get restless and fidgety?	Score Range: Yes; No
Prefer to stay at home	StayatHome36	Do you prefer to stay at home, rather than going out and doing new things?	Score Range: Yes; No
Worry about the future	WorryAbtFut36	Do you frequently worry about the future?	Score Range: Yes; No
Problems with memory	MemProb36	Do you feel you have more problems with memory than most?	Score Range: Yes; No
Wonderful to be alive	Alive36	Do you think it is wonderful to be alive now?	Score Range: Yes; No

Feel downhearted and blue	DownheartedBlue36	Do you often feel downhearted and blue?	Score Range: Yes; No
Feel worthless	Worthless36	Do you feel pretty worthless the way you are now?	Score Range: Yes; No
Worry about the past	WorryAbtPast36	Do you worry a lot about the past?	Score Range: Yes; No
Find life exciting	LifeExciting36	Do you find life very exciting?	Score Range: Yes; No
Hard to get started on new projects	StartNewProj36	Is it hard for you to get started on new projects?	Score Range: Yes; No
Feel full of energy	FullEnergy36	Do you feel full of energy?	Score Range: Yes; No
Assessment 37—Center for Epidemiological Studies-Depression	Assess37	1 = Has data<br>2 = Assessment data partial<br>3 = No data	
I was bothered by things that usually don't bother me	Bothered37	During the last week, I was bothered by things that usually don't bother me.	Score Range: Rarely (Less than 1 Day); Some (1-2 Days); Occasionally (3-4 Days); Frequently (5-7 Days)
I did not feel like eating, my appetite was poor	PoorAppetite37	During the last week, I did not feel like eating, my appetite was poor.	Score Range: Rarely (Less than 1 Day); Some (1-2 Days); Occasionally (3-4 Days); Frequently (5-7 Days)
I felt that I could not shake off the blues even with the help from my family or friends	CouldNotShakeBlues37	During the last week, I felt that I could not shake off the blues even with the help from my family or friends.	Score Range: Rarely (Less than 1 Day); Some (1-2 Days); Occasionally (3-4 Days); Frequently (5-7 Days)
I felt that I was just as good as other people	JustAsGood37	During the last week, I felt that I was just as good as other people.	Score Range: Rarely (Less than 1 Day); Some (1-2 Days); Occasionally (3-4 Days); Frequently (5-7 Days)
I had trouble keeping my mind on what I was doing	TroubleKeepMind37	During the last week, I had trouble keeping my mind on what I was doing.	Score Range: Rarely (Less than 1 Day); Some (1-2 Days); Occasionally (3-4 Days); Frequently (5-7 Days)
I felt depressed	Depressed37	During the last week, I felt depressed.	Score Range: Rarely (Less than 1 Day); Some (1-2 Days); Occasionally (3-4 Days); Frequently (5-7 Days)
I felt that everything I did was an effort	EverythingEffort37	During the last week, I felt that everything I did was an effort.	Score Range: Rarely (Less than 1 Day); Some (1-2 Days); Occasionally (3-4 Days); Frequently (5-7 Days)
I felt hopeful about the future	HopefulFuture37	During the last week, I felt hopeful about the future.	Score Range: Rarely (Less than 1 Day); Some (1-2 Days); Occasionally (3-4 Days); Frequently (5-7 Days)
I thought my life had been a failure	LifeFailure37	During the last week, I thought my life had been a failure.	Score Range: Rarely (Less than 1 Day); Some (1-2

			Days); Occasionally (3-4 Days); Frequently (5-7 Days)
I felt fearful	Fearful37	During the last week, I felt fearful.	Score Range: Rarely (Less than 1 Day); Some (1-2 Days); Occasionally (3-4 Days); Frequently (5-7 Days)
My sleep was restless	SleepRestless37	During the last week, my sleep was restless.	Score Range: Rarely (Less than 1 Day); Some (1-2 Days); Occasionally (3-4 Days); Frequently (5-7 Days)
I was happy	Happy37	During the last week, I was happy.	Score Range: Rarely (Less than 1 Day); Some (1-2 Days); Occasionally (3-4 Days); Frequently (5-7 Days)
I talked less than usual	TalkedLess37	During the last week, I talked less than usual.	Score Range: Rarely (Less than 1 Day); Some (1-2 Days); Occasionally (3-4 Days); Frequently (5-7 Days)
I felt lonely	Lonely37	During the last week, I felt lonely.	Score Range: Rarely (Less than 1 Day); Some (1-2 Days); Occasionally (3-4 Days); Frequently (5-7 Days)
People were unfriendly	PeopleUnfriendly37	During the last week, people were unfriendly.	Score Range: Rarely (Less than 1 Day); Some (1-2 Days); Occasionally (3-4 Days); Frequently (5-7 Days)
I enjoyed life	EnjoyedLife37	During the last week, I enjoyed life.	Score Range: Rarely (Less than 1 Day); Some (1-2 Days); Occasionally (3-4 Days); Frequently (5-7 Days)
I had crying spells	CryingSpells37	During the last week, I had crying spells.	Score Range: Rarely (Less than 1 Day); Some (1-2 Days); Occasionally (3-4 Days); Frequently (5-7 Days)
I felt sad	Sad37	During the last week, I felt sad.	Score Range: Rarely (Less than 1 Day); Some (1-2 Days); Occasionally (3-4 Days); Frequently (5-7 Days)
I felt that people dislike me	PeopleDislikeMe37	During the last week, I felt that people dislike me.	Score Range: Rarely (Less than 1 Day); Some (1-2 Days); Occasionally (3-4 Days); Frequently (5-7 Days)
I could not get going	CouldNotGetGoing37	During the last week, I could not get going.	Score Range: Rarely (Less than 1 Day); Some (1-2 Days); Occasionally (3-4 Days); Frequently (5-7 Days)
Assessment 38—Alzheimer's Disease Assessment Scale (ADAS)-Cognitive Subscale	Assess38	1 = Has data<br>2 = Assessment data partial<br>3 = No data	
ADAS Word Recall Trial 1	ADASRcll1_38	Number of words recalled during trial 1	Score Range: 0-10

ADAS Word Recall Trial 2	ADASRcll2_38	Number of words recalled during trial 2	Score Range: 0-10
ADAS Word Recall Trial 3	ADASRcll3_38	Number of words recalled during trial 3	Score Range: 0-10
ADAS Naming Objects and Fingers	ADASNaming38	Number of fingers and objects named incorrectly	Score Range: 0-5<br>0 = 0-2 incorrect<br>1 = 3-5 incorrect<br>2 = 6-8 incorrect<br>3 = 9-11 incorrect<br>4 = 12-14 incorrect<br>5 = 15-17 incorrect
ADAS Commands	ADASComnds38	Number of commands incorrectly performed	Score Range: 0-5
ADAS Constructional Praxis	ADASConsPrax38	Number of forms drawn incorrectly	Score Range: 0-5<br>0 = 0 incorrect<br>1 = 1 incorrect<br>2 = 2 incorrect<br>3 = 3 incorrect<br>4 = 4 incorrect (but one or more section was drawn)<br>5 = No figures drawn, no recognizable attempt at drawing any side/section of any figure
ADAS Ideational Praxis	ADASIdeaPrax38	Number of components completed incorrectly	Score Range: 0-5
ADAS Orientation	ADASOrient38	Number of correct responses. One point is given for each incorrect response.	Score Range: 0-8
ADAS Word Recognition	ADASRcg38	Mean number of correct responses given throughout the three trials	Score Range: 0-12
ADAS Language	ADASLang38	Language Ability. Lower scores indicate more fluent speech.	Score Range: 0-5<br>0 = subject speaks clearly and/or is understandable<br>1 = very mild: one instance of lack of understandability<br>2 = mild: subject has difficulty less than 25% of the time<br>3 = moderate: subject has difficulty 25-50% of the time<br>4 = moderately severe: subject has difficulty more than 50% of the time<br>5 = severe: one- or two-word utterances; fluent, but empty speech; mute
ADAS Comprehension of Spoken Language	ADASSpokenLang38	How well the participant understands spoken language. Lower scores indicate greater comprehension.	Score Range: 0-5<br>0 = None: subject understands.

			1 = Very Mild: one or two instances of misunderstanding.<br>2 = Mild: 3–5 instances of misunderstanding.<br>3 = Moderate: requires several repetitions and rephrasing.<br>4 = Moderately Severe: subject only occasionally responds correctly, i.e., yes-or-no questions.<br>5 = Severe: subject rarely responds to questions appropriately; not due to poverty of speech.
ADAS Word Finding Difficulty	ADASWordFindDiff38	The level of difficulty a participant demonstrates in finding desired words	Score Range: 0-5<br>0 = no evidence of word finding difficulty in spontaneous speech<br>1 = very mild: 1 or 2 instances, not clinically significant;<br>2 = mild: noticeable circumlocution or synonym substitution;<br>3 = moderate: loss of words without compensation on occasion;<br>4 = moderately severe: frequent loss of words without compensation;<br>5 = severe: nearly total loss of content of words; speech sounds empty; 1–2-word utterances
ADAS Remembering Test Instructions	ADASRemInstruct38	Number of times a participant needs to be reminded of instructions	Score Range: 0-5<br>0 = subject never needs extra reminders of instructions<br>1 = very mild: forgets once<br>2 = mild: must be reminded 2 times<br>3 = moderate: must be reminded 3 or 4 times<br>4 = moderately severe: must be reminded 5 or 6 times<br>5 = severe: must be reminded 7 or more times

Mental Health and AD Screening Data Set: Instruments

Assessment 9.36 Geriatric Depression Scale

This survey asks for your views about your moods. For each question, choose the response that best describes your answer.

1. Are you basically satisfied with your life?
2. Have you dropped many of your activities or interests?
3. Do you feel that your life is empty?
4. Do you often get bored?
5. Are you hopeful about the future?
6. Are you bothered by thoughts you can't get out of your head?
7. Are you in good spirits most of the time?
8. Are you afraid that something bad is going to happen to you?
9. Do you feel happy most of the time?
10. Do you often feel hopeless?
11. Do you often get restless and fidgety?
12. Do you prefer to stay at home, rather than going out and doing new things?
13. Do you frequently worry about the future?
14. Do you feel you have more problems with memory than most?
15. Do you think it is wonderful to be alive right now?
16. Do you often feel downhearted and blue?
17. Do you feel pretty worthless the way you are now?
18. Do you worry a lot about the past?
19. Do you find life very exciting?
20. Is it hard for you to get started on new projects?
21. Do you feel full of energy?

Assessment 9.37 Center for Epidemiological Studies-Depression (CESD)

For this survey, respond about how you felt or behaved **DURING THE PAST WEEK.** For each question, choose the one response that best describes your answer.

1. During the last week, I was bothered by things that usually don't bother me.
2. During the last week, I did not feel like eating my appetite was poor.
3. During the last week, I felt that I could not shake off the blues even with the help from my family or friends.
4. During the last week, I felt that I was just as good as other people.
5. During the last week, I had trouble keeping my mind on what I was doing.
6. During the last week, I felt depressed.
7. During the last week, I felt that everything I did was an effort.
8. During the last week, I felt hopeful about the future.
9. During the last week, I thought my life had been a failure.
10. During the last week, I felt fearful.
11. During the last week, my sleep was restless.
12. During the last week, I was happy.

13. During the last week, I talked less than usual.
14. During the last week, I felt lonely.
15. During the last week, people were unfriendly.
16. During the last week, I enjoyed life.
17. During the last week, I had crying spells.
18. During the last week, I felt sad.
19. During the last week, I felt that people dislike me.
20. During the last week, I could not get going.

Assessment 9.38 Alzheimer's Disease Assessment Scale- Cognitive Subscale (ADAS-Cog)

For ADAS-Cog instrument and scoring manual, please see the FDA website:
https://www.fda.gov/media/122843/download

Construct 10: Psychosocial

Table of Contents

Sample Sizes By Epoch and Task

Section 1: Brief Descriptions of Psychosocial Questionnaires Presented to Subjects
- Martin and Park Environmental Demands (MPED) Questionnaire
- Daily Activities Questionnaire
- Lifetime Cognitive Activities
- Need for Cognition Survey (NFC)
- Metamemory in Adulthood (MIA) Questionnaire
- Self-Concept Clarity (SCC) Survey
- Satisfaction with Life Scale
- Revised Neuroticism-Extraversion-Openness Personality Inventory (NEO-PI-R)
- Big 5 Inventory
- Personality Survey
- NIH Toolbox Emotion Measures
- Scale of Positive and Negative Experience (SPANE)
- Psychological Well-being (SWQ)

Section 2: Access to Psychosocial Questionnaire Summary Data
- Martin and Park Environmental Demands (MPED) Questionnaire
- Daily Activities Questionnaire
- Lifetime Cognitive Activities
- Need for Cognition Survey (NFC)
- Metamemory in Adulthood (MIA) Questionnaire
- Self-Concept Clarity (SCC) Survey
- Satisfaction with Life Scale
- Revised Neuroticism-Extraversion-Openness Personality Inventory (NEO-PI-R)
- Big 5 Inventory
- Personality Survey
- NIH Toolbox Emotion Measures
- Scale of Positive and Negative Experience (SPANE)
- Psychological Well-being (SWQ)

Section 3: Instruments
- Martin and Park Environmental Demands (MPED) Questionnaire
- Daily Activities Questionnaire
- Lifetime Activities Questionnaire
- Need for Cognition (NFC)
- Metamemory in Adulthood (MIA) Questionnaire
- Self-Concept Clarity (SCC) Survey
- Satisfaction with Life Scale
- Big 5 Inventory
- Personality Survey

NIH Toolbox Emotion Measures
Scale of Positive and Negative Experience (SPANE)
Psychological Well-being (SWQ)

Sample Sizes by Epoch and Assessment (subjects with partial data in parentheses)

Assessment	Epoch 1	Epoch 2	Epoch 3
**MPED**	460	291	152
**DAQ**	460	290(1)	151
**LCA**	460	291	154(1)
**NFC**	460	292	151
**Metamemory**	0	283(1)	132(1)
**SCC**	460	288	144
**SLS**	460	289	149
**NEO-PI-R**	463	0	0
**Big5**	0	288	136
**Personality**	459	0	0
**NIH Emotion**	0	297(297)[a]	201
**SPANE**	90	286	143
**SWQ**	460(73)[b]	288(37)[b]	146(24)[b]

Notes on data completeness:
[a]NIH Emotion: For Epoch 2, the summary scores for negative affect, well-being, and social satisfaction are unavailable.
[b]SWQ: For Epochs 1, 2, and 3, total scores are only available for 387, 251, and 122 participants, respectively.

Task Descriptions

Assessment 10.39 Martin and Park Environmental Demands (MPED) Questionnaire

Description: This assessment evaluates a participant's level of daily busyness and routines. The original published questionnaire had 11 questions: 7 items for busyness and 4 items for routines. The DLBS questionnaire included two additional questions relating to forgetfulness. On one question, they rated how busy they are during an average day using a five-point Likert scale ranging from "not busy at all" to "extremely busy"; the remaining items asked how often they did various actions on a five-point scale ranging from "never" to "very often".

Scoring: The variables of interest are a 7-item total busyness score, total routines score, total forgetfulness score, and a 9-item total busyness score.

Primary Reference:
Martin, M. & Park, D. C. (2003). The Martin and Park Environmental Demands (MPED) Questionnaire: Psychometric properties of a brief instrument to measure self-reported environmental demands. *Aging Clinical and Experimental Research, 15*(1), 77-82. https://doi.org/10.1007/BF03324483

Assessment 10.40 Daily Activities Questionnaire

Description: Participants were asked how often they participated in the activities that follow within the last six months: how often they grocery shop, drive a car, do household repairs, etc. Participants selected the response from: a). Never; b). Less than once every 6 months; c). Once every 6 months; d). 2 or 3 times every 6 months; e). Once a month; f). 2 or 3 once a month; g). Once a week; h). 2 or 3 times a week; i). Daily. This questionnaire has 70 questions.

Scoring: Summary scores are available for each of the seven factors: Physical, Self-Maintenance, Social, Integrative Information Getting, Passive Information Processing, Novel Information Processing, and Travel.

Primary Reference:

Hultsch, D. F., Hertzog, C., Small, B. J., & Dixon, R. A. (1999). Use it or lose it: Engaged lifestyle as a buffer of cognitive decline in aging? *Psychology and Aging, 14*(2), 245-263. https://doi.org/10.1037/0882-7974.14.2.245

● ***Note:*** Please note that Hultsch et al., 1999 called the factor integrative information getting "Hobbies and home maintenance activities".

Assessment 10.41 Lifetime Cognitive Activities

Description: Participants were asked about the frequency with which they participated in cognitively stimulating activities in the past and present. This questionnaire has 26 questions. Each set of questions refers to a specific time period in their life: age 6 (3 items), age 12 (six items), age 18 (six items), age 40 (five items), and current age (five items). Participants selected their response from: a). Once a year; b). Several times a year; c). Several times a month; d). Several times a week; e). Every day or about every day.

Scoring: A total score, lifetime cognitive activities to 18 years old score, lifetime cognitive activities to 40 years old score, and past lifetime cognitive activities for participants over 40 score are available. Higher scores are indicative of more frequent cognitive activity. Additionally, a DLBS-specific score is available to identify participants who are younger than 40 years old.

Primary Reference:

Wilson, R., Barnes, L., & Bennett, D. (2003). Assessment of lifetime participation in cognitively stimulating activities. *Journal of Clinical and Experimental Neuropsychology, 25*(5), 634-642. https://doi.org/10.1076/jcen.25.5.634.14572

Assessment 10.42 Need for Cognition Survey (NFC)

Description: Cacioppo et al. (1984) operationalize the need for cognition as "the tendency for an individual to engage in and enjoy effortful cognitive endeavors" (p. 306). Participants rated whether or not each statement was characteristic of them on a five-point Likert scale ranging from "extremely uncharacteristic" to "extremely characteristic". This questionnaire has 18 questions.

Scoring: A need for cognition total score is available.

Primary Reference:

Cacioppo, J. T., Petty, R. E., & Kao, C. F. (1984). The efficient assessment of need for cognition. *Journal of Personality Assessment, 48*(3), 306-307. https://doi.org/10.1207/s15327752jpa4803_13

Assessment 10.43 Metamemory in Adulthood (MIA) Questionnaire

Description: This assessment measures knowledge and beliefs about one's own memory functioning. This questionnaire has 108 questions. The following abilities are assessed: use of memory strategies (strategy), knowledge of memory tasks (task), knowledge of own memory capacities (capacity), attitudes towards own memory: perception of change, memory and state anxiety (anxiety), memory and achievement motivation (achievement), and locus of control in memory abilities (locus). For the first 18 items, participants rated on a five-point Likert scale how often they did a behavior from "never" to "always"; for the remaining items, they rated on a five-point Likert scale how much they agreed with various statements from "strongly disagree" to "strongly agree".

Scoring: Total scores are available for all seven subscales: strategy, task, capacity, change, anxiety, achievement, and locus.

Primary Reference:

Dixon, R. A., Hultsch, D. F., & Hertzog, C. (1988). The Metamemory in Adulthood (MIA) questionnaire. *Psychopharmacology bulletin, 24*(4), 671-688.

Assessment 10.44 Self-Concept Clarity (SCC) Survey

Description: Participants rated how well statements regarding the clarity of their concept of self applied to them on a five-point Likert scale ranging from "disagree strongly" to "agree strongly" (e.g., "My beliefs about myself seem to change very frequently"). They were asked to rate themselves as they generally are now, not as they wish to be in the future. Self-concept clarity is defined by Campbell et al. (1996) as the extent to which "the contents of an individual's self-concept (e.g., perceived personal attributes) are clearly and confidently defined, internally consistent, and temporally stable" (p. 141). This task consists of 12 questions. The variable of interest is the total score of self-concept clarity.

Scoring: A Self-Concept Clarity total score is available and calculated using questions 1-12.

Primary Reference:

Campbell, J. D., Trapnell, P. D., Heine, S. J., Katz, I. M., Lavallee, L. F., & Lehman, D. R. (1996). Self-concept clarity: Measurement, personality correlates, and cultural boundaries. *Journal of Personality and Social Psychology, 70*(1), 141-156. https://doi.org/10.1037/0022-3514.70.1.141

Assessment 10.45 Satisfaction with Life Scale

Description: Participants were asked for their views about their life in the attempt to assess global life satisfaction. This questionnaire has 5 questions requiring a rating on a seven-point

Likert scale ranging from "strongly disagree" to "strongly agree". A self-reflection composite score is available.

Scoring: A Satisfaction with Life total score is available and calculated using questions 1-5.

Primary Reference:

Diener, E., Emmons, R. A., Larsen, R. J., & Griffin, S. (1985). The Satisfaction With Life Scale. *Journal of Personality Assessment, 49*(1), 71-75. https://doi.org/10.1207/s15327752jpa4901_13

Assessment 10.46 Revised Neuroticism-Extraversion-Openness Personality Inventory (NEO-PI-R)

Description: Please note that this questionnaire was administered on Day 2 of Cognitive Testing, however, it is a comprehensive measure of adult personality, and thus, is included with the psychosocial data. Participants were asked to rate how well a statement corresponds to their personality on a five-point Likert scale from "strongly disagree" to "strongly agree". This questionnaire has 240 questions that assess five major dimensions of personality: Neuroticism, Extraversion, Openness, Agreeableness, and Conscientiousness. In addition, there are six facets or traits that further define each dimension. Only scores are available, refer to publications for questionnaires.

Scoring: Summary scores are available for each of the five dimensions: Neuroticism, Extraversion, Openness, Agreeableness, and Conscientiousness. Each of these dimensions also has six corresponding facet scores (e.g., Neuroticism-Anxiety, Neuroticism-Depression).

Primary References:

Costa P.T., Jr, & McCrae R.R. (1992). Revised NEO Personality Inventory (NEO-PI-R) and NEO Five-Factor Inventory (NEO-FFI) professional manual. Odessa, FL: Psychological Assessment Resources.

Costa, P. T., Jr, & McCrae, R. R. (1995). Domains and facets: hierarchical personality assessment using the revised NEO personality inventory. *Journal of personality assessment, 6*4(1), 21–50.

● ***Note:*** Please note that the NEO-PI-R was only administered in Epoch 1. Personality was assessed in Epochs 2-3 with the Big Five Inventory.

Assessment 10.47 Big 5 Inventory

Description: Participants were asked to indicate how well a statement pertaining to personality applied to them on a five-point Likert scale from "strongly disagree" to "strongly agree". This questionnaire has 44 items and is designed to measure the Big Five dimensions of personality.

Scoring: Summary scores are available for each of the five dimensions: Extraversion, Agreeableness, Conscientiousness, Neuroticism, and Openness.

Primary Reference:

John, O. P., Donahue, E. M., & Kentle, R. L. (1991). The Big Five Inventory - Versions 4a and 54. Berkeley, CA: University of California, Berkeley, Institute of Personality and Social Research. https://doi.org/10.1037/t07550-000

● ***Note:*** Please note that this inventory was first administered in Epoch 2 and was our primary personality inventory for Epochs 2-3.

Assessment 10.48 Personality Survey

Description: Participants rated how accurately different statements pertaining to personality applied to themselves on a five-point Likert scale from "very inaccurate" to "very accurate". They were asked to make these ratings based on how they generally are now, not as they wish to be in the future. This questionnaire has 108 questions.

Scoring: There are two composites: Conscientiousness and Openness. The Conscientiousness composite is made up of seven facets and the Openness composite is comprised of four facets. Summary scores are available for each of the eleven facets: orderliness, virtue, traditionalism, self-control, responsibility, industriousness, intellect, ingenuity, competence, quickness, and creativity.

Primary References:

Roberts, B. W., Chernyshenko, O. S., Stark, S., & Goldberg, L. R. (2005). The structure of conscientiousness: An empirical investigation based on seven major personality questionnaires. *Personnel Psychology, 58*(1), 103–139. https://doi.org/10.1111/j.1744-6570.2005.00301.x

Goldberg, L. R., Johnson, J. A., Eber, H. W., Hogan, R., Ashton, M. C., Cloninger, C. R., et al. (2006). The International Personality Item Pool and the future of public- domain personality measures. *Journal of Research in Personality, 40*, 84–96. http://dx.doi.org/10.1016/j.jrp.2005.08.007.

● ***Note:*** Please note that the individual facets tend to be better and differential predictors of numerous outcomes than the composite measures. Also note that the Personality Survey was only administered in Epoch 1. Personality was assessed in Epochs 2-3 with the Big Five Inventory.

Assessment 10.49 NIH Toolbox Emotion Measures

Description: This battery consists of self-report surveys assessing 17 subdomains of emotion:

- *NIH Toolbox Anger-Affect Survey:* This CAT (computer adaptive test) assesses anger as an emotion. Participants respond using a 5-point scale ranging from "never" to "always."
- *NIH Toolbox Anger-Hostility Survey:* This 5-item fixed form survey assesses attitudes of hostility and cynicism. Participants respond using a 7-point scale ranging from "extremely untrue of me" to "extremely true of me."

- *NIH Toolbox Anger-Physical Aggression Survey:* This 5-item fixed form survey assesses aggression as a behavioral component. Participants respond using a 7-point scale ranging from "extremely untrue of me" to "extremely true of me."
- *NIH Toolbox Emotional Support Survey:* This 8-item fixed form survey assesses emotional support. Participants respond using a 5-point scale ranging from "never" to "always."
- *NIH Toolbox Fear-Affect Survey:* This CAT self-report measure assesses fear and anxious misery. Participants respond using a 5-point scale ranging from "never" to "always."
- *NIH Toolbox Fear-Somatic Arousal Survey:* This 6-item fixed form survey assesses somatic symptoms related to arousal. Participants respond using a 5-point scale ranging from "not at all" to "extremely."
- *NIH Toolbox Friendship Survey:* This 8-item fixed form survey assesses perceptions of friendship. Participants respond using a 5-point scale ranging from "never" to "always."
- *NIH Toolbox General Life Satisfaction Survey:* This CAT assesses global feelings and attitudes about one's life. Participants respond using a 5-point or 7-point scale—depending on item—ranging "strongly disagree" to "strongly agree."
- *NIH Toolbox Instrumental Support Survey:* This 8-item fixed form survey assesses instrumental support. Participants respond using a 5-point scale ranging from "never" to "always."
- *NIH Toolbox Loneliness Survey:* This 5-item fixed form survey assesses perceptions of loneliness. Participants respond using a 5-point scale ranging from "never" to "always."
- *NIH Toolbox Meaning and Purpose Survey:* This CAT self-report assesses the extent to which participants feel that their life matters or makes sense. Participants respond using a 5-point scale ranging from "strongly disagree" to "strongly agree," or from "not at all" to "very much."
- *NIH Toolbox Perceived Hostility Survey*: This fixed form survey assesses perceptions of hostility. Participants respond using a 5-point scale ranging from "never" to "always."
- *NIH Toolbox Perceived Rejection Survey:* This 8-item fixed form survey assesses perceptions of rejection. Participants respond using a 5-point scale ranging from "never" to "always."
- *NIH Toolbox Perceived Stress Survey:* This fixed form survey assesses how unpredictable, uncontrollable and overloaded respondents find their lives. Participants respond using a 5-point scale ranging from "never" to "very often."
- *NIH Toolbox Positive Affect Survey*: This CAT assesses both activated (i.e., happiness, joy) and unactivated (i.e., serenity, peace) aspects of positive affect. Participants respond using a 5-point scale ranging from "not at all" to "very much."
- *NIH Toolbox Sadness Survey:* This CAT self-report measure assesses negative mood, negative views of the self, and negative social cognition. Participants respond using a 5-point scale ranging from "never" to "always."

- *NIH Toolbox Self-Efficacy Survey:* This CAT self-report assesses respondents' sense of global self-efficacy. Participants respond using a 5-point scale ranging from “never” to “very often.”

Scoring: Raw scores, Thetas, T-scores, and SEs are available for all surveys listed above. Scores 1 SD or more below the mean (T ≤ 40) suggest low levels, scores 1 SD or more above the mean (T ≥ 60) suggest high levels, and T-scores ≤ 40 may warrant heightened surveillance or concern.

● ***Caution:*** Participants in DLBS Epoch 2 performed the NIH Toolbox Emotion Measures on a desktop computer, whereas, participants in DLBS Epoch 3 performed the task on an iPad. For additional details, we refer you to the NIH Toolbox website: https://www.healthmeasures.net/explore-measurement-systems/nih-toolbox/obtain-and-administer-measures.

Primary Reference:

Gershon R. C., Wagster M. V., Hendrie H. C., Fox N. A., Cook K. F., & Nowinsky C. J. (2013). NIH Toolbox for assessment of neurological and behavioral function. Neurology, *80*, S1-S92. https://doi.org/10.1212/WNL.0b013e3182872e5f

Software and Scoring Reference:

NIH Toolbox for the iPad test ver. 2.1 https://nihtoolbox.force.com/s/article/nih-toolbox-scoring-and-interpretation-guide

Assessment 10.50 Scale of Positive and Negative Experience (SPANE)

Description: Participants were asked about what they have been doing and experiencing during the past four weeks. They were asked to report how frequently they experienced various emotions on a five-point Likert scale from “very rarely or never” to 5 “very often or always”. This questionnaire has 12 questions.

Scoring: A score for the frequency of positive emotions (6 questions), frequency of negative emotions (6 questions), and a total “balance” score of positive and negative emotions was derived.

Primary Reference:

Diener, E., Wirtz, D., Tov, W., Kim-Prieto, C., Choi, DW, Oishi, S., & Biswas-Diener, R. (2010). New well-being measures: Short scales to assess flourishing and positive and negative feelings. *Social Indicator Research, 97*(2), 143-156. https://doi.org/10.1007/s11205-009-9493-y

● ***Note:*** Please note that this questionnaire was only administered to a partial sample of participants in Epoch 1 (n = 90)

Assessment 10.51 Psychological Well-being (SWQ)

Description: Participants were asked to rate how well statements pertaining to psychological well-being applied to them on five-point Likert scale from “strongly disagree” to “strongly agree”. This questionnaire has 84 questions. There are six factors: positive relations with others,

autonomy, environmental mastery, personal growth, purpose in life, and self-acceptance, as well as a composite score.

Scoring: The following items were used to produce each of the 6 factors (reverse score *)

- Positive Relations with Others (1, 7*, 13*, 19, 25, 31*, 37, 43*, 49, 55*, 61*, 67, 73*, 79)
- Autonomy (2*, 8, 14, 20*, 26, 32*, 38, 44*, 50, 56*, 62*, 68, 74*, 80)
- Environmental Mastery (3, 9*, 15*, 21, 27*, 33, 39, 45*, 51, 57, 63*, 69, 75*, 81)
- Personal Growth (4*, 10, 16, 22*, 28, 34*, 40, 46, 52, 58*, 64, 70, 76*, 82*)
- Purpose in Life (5, 11*, 17*, 23, 29*, 35*, 41*, 47, 53, 59, 65*, 71, 77, 83*)
- Self-acceptance (6, 12, 18*, 24*, 30, 36, 42*, 48, 54*, 60*, 66*, 72, 78, 84*)

Primary Reference:

Ryff, C. D. (1989). Happiness is everything, or is it? Explorations on the meaning of psychological well-being. *Journal of Personality and Social Psychology, 57*(6), 1069-1081. https://doi.org/10.1037/0022-3514.57.6.1069

● ***Note:*** This questionnaire is publicly available, but Dr. Ryff requests that institutions or organizations provide her with the results of their study and any subsequent journal article citations. Dr. Carol Ryff; University of Wisconsin; Institute on Aging; 2245 Medical Sciences Center; 1300 University Avenue; Madison, WI 53706; Phone: (608) 262-1818; Fax: (608) 263-6211; email:cryff@wisc.edu.

Psychosocial Construct: Key to Names and Data Structure in Data Set

Item Name	Abbreviation	Description	Measurement
Subject Number	S#	Subject identifier	
Construct Name	ConstructName	Psychosocial	
Construct Number	ConstructNumber	Construct 10	
Epoch	Wave	Denotes the data collection Epoch. See individual differences data set for more detail, including testing date intervals.	1 = Epoch 1<br>2 = Epoch 2<br>3 = Epoch 3
Has Data	HasData	1 = Yes, returned for Epoch;<br>2 = No, did not return for Epoch	
Number of Assessments in Data Set	NumAssess	How many assessments make up the psychosocial data set	13 Assessments for Psychosocial
Assessment 39—Martin and Park Environmental Demands	Assess39	1 = Has data<br>2 = Assessment data partial<br>3 = No data	
Martin and Park Environmental Demands Busyness Total 7 Items	MPEDBusyness7_39	Summary score for 7-item busyness measure. Average of questions 1-5,	Score Range: 1-5

		8, 9. Higher scores reflect greater busyness.<br>Q. 1: daily busyness<br>Q. 2: too many tasks<br>Q. 3: rushing to places<br>Q. 4: missing rest<br>Q. 5: missing meals<br>Q. 8: rushed mornings<br>Q. 9: delayed bedtime	
Martin and Park Environmental Demands Routines Total	MPEDRoutines39	Summary score for routines measure. Average of questions 10-13. Higher scores reflect greater routine implementation.<br>Q. 10: follow routine<br>Q. 11: routine sleep<br>Q. 12: routine meals<br>Q. 13: routine activities	Score Range: 1-5
Martin and Park Environmental Demands Forgetfulness Total	MPEDForgetful39	Summary score for forgetfulness measure. Average of questions 6 & 7. Higher scores indicate more forgetfulness.<br>Q. 6: forget tasks<br>Q. 7: forget medications	Score Range: 1-5
Martin and Park Environmental Demands Busyness Total 9 Items	MPEDBusyness9_39	Summary score for 9-item busyness measure, which includes the 2 self-developed forgetfulness items. Higher scores reflect greater busyness and this is an average of the below questions.<br>Q. 1: daily busyness<br>Q. 2: too many tasks<br>Q. 3: rushing to places<br>Q. 4: missing rest<br>Q. 5: missing meals<br>Q. 6: forget tasks<br>Q. 7: forget medications<br>Q. 8: rushed mornings<br>Q. 9: delayed bedtime	Score Range: 1-5
Assessment 40—Daily Activities Questionnaire	Assess40	1 = Has data<br>2 = Assessment data partial<br>3 = No data	
Daily Activities Questionnaire Physical	ActivPhysical40	Average score for how often one engages in physical activity, such as gardening, walking, or playing tennis	Score Range: 1-9
Daily Activities Questionnaire Self-maintenance	ActivSelfMain40	Average score for how often one engages in self-maintenance activities,	Score Range: 1-9

		such as preparing a meal or grocery shopping.	
Daily Activities Questionnaire Social	ActivSocial40	Average score for how often one engages in social activities, such as visiting friends.	Score Range: 1-9
Daily Activities Questionnaire Integrative	ActivInteg40	Average score for how often one engages in hobbies and home maintenance activities, such as playing an instrument or repairing mechanical items	Score Range: 1-9
Daily Activities Questionnaire Passive Information Processing	ActivPassInfoProc40	Average score for passive information processing, such as listening to the radio or watching a sporting event. Higher scores denote more passive information processing.	Score Range: 1-9
Daily Activities Questionnaire Novel Information Processing	ActivNovelInfoProc40	Average score for how often one engages in novel processing activities, such as driving a car or doing a crossword puzzle.	Score Range: 1-9
Daily Activities Questionnaire Travel	ActivTravel40	Average score for how often one performs traveling, such as outside one's home state.	Score Range: 1-9
Assessment 41—Lifetime Cognitive Activities Questionnaire	Assess41	1 = Has data<br>2 = Assessment data partial<br>3 = No data	
Lifetime Cognitive Activities Total	LCATot41	Average score for total lifetime cognitive activities. Higher scores denote greater cognitive activity engagement during lifetime.	Score Range: 1-5
Lifetime Cognitive Activities to 18 Years Old	LCA18_41	Average score for lifetime cognitive activities until age 18. Higher scores denote greater engagement in lifetime cognitive activities.	Score Range: 1-5
Lifetime Cognitive Activities to 40 Years Old	LCA40_41	Average score for lifetime cognitive activities until age 40. Higher scores denote greater engagement in lifetime cognitive activities.	Score Range: 1-5
Younger than 40 Years Old	LCAYT40_41	Are you age 41 or older?	Score Range: 0-1<br>0 = Yes<br>1 = No

Past Lifetime Cognitive Activities for Participants Under 40	LCAPastYT40_41	Average score for past lifetime cognitive activities for individuals younger than 40 years-old. Higher scores denote greater cognitive activity engagement.	Score Range: 1-5
Assessment 42—Need for Cognition Survey	Assess42	1 = Has data<br>2 = Assessment data partial<br>3 = No data	
Need for Cognition Total	NFCTot42	Average score questions 1-18, which assess one's need to engage in cognitively effortful endeavors. Higher scores denote greater need for cognition.	Score Range: 1-5
Assessment 43—Metamemory in Adulthood Questionnaire	Assess43	1 = Has data<br>2 = Assessment data partial<br>3 = No data	
Metamemory in Adulthood Questionnaire Strategy	MemStrategy43	Knowledge and use of information about one's remembering abilities so that performance in given instances is potentially improved. High scores indicate greater use of strategy.	Score Range: 0-90
Metamemory in Adulthood Questionnaire Task	MemTask43	Knowledge of basic memory processes, especially that are interesting as evidenced by how most people perform. High scores indicate high knowledge.	Score Range: 0-75
Metamemory in Adulthood Questionnaire Capacity	MemCapacity43	Perception of memory capacities as evidenced by rating of performance on given tasks. High scores indicate greater capacity.	Score Range: 0-85
Metamemory in Adulthood Questionnaire Change	MemChange43	Perception of memory abilities as generally stable or subject to long-term decline. High scores indicate greater stability.	Score Range: 0-85
Metamemory in Adulthood Questionnaire Anxiety	MemAnxiety43	Feelings of stress related to memory performance. High scores indicate greater anxiety.	Score Range: 0-70
Metamemory in Adulthood Questionnaire Achievement	MemAchieve43	Perceived importance of having a good memory and performing well on	Score Range: 0-80

		memory tasks. High scores indicate high achievement.	
Metamemory in Adulthood Questionnaire Locus	MemLocus43	Perceived personal control over remembering abilities. High scores indicate an internal locus of control.	Score Range: 0-50
Assessment 44—Self-Concept Clarity Survey	Assess44	1 = Has data<br>2 = Assessment data partial<br>3 = No data	
Self-Concept Clarity Total	SCCTot44	Average score of how clear, consistent, and stable one's self-concept is. High score indicates greater self-concept clarity.	Score Range: 1-5
Assessment 45—Satisfaction with Life Scale	Assess45	1 = Has data<br>2 = Assessment data partial<br>3 = No data	
Satisfaction with Life Total	SatisfacLifeTot45	Average score of one's life satisfaction. High score indicates greater life satisfaction.	Score Range: 1-7
Assessment 46—Revised Neuroticism-Extraversion-Openness Personality Inventory	Assess46	1 = Has data<br>2 = Assessment data partial<br>3 = No data	
NEO PI-R Neuroticism: Anxiety	NEONeurAnx46	Average score of anxiety. Higher scores here, and for the below NEO variables, indicate stronger trait expression.	Score Range: 0-4
NEO PI-R Neuroticism: Anger Hostility	NEONeurAngerHos46	Average score of anger and hostility.	Score Range: 0-4
NEO PI-R Neuroticism: Depression	NEONeurDep46	Average score of depression.	Score Range: 0-4
NEO PI-R Neuroticism: Self-Consciousness	NEONeurSelfCon46	Average score of self-consciousness.	Score Range: 0-4
NEO PI-R Neuroticism: Impulsiveness	NEONeurImp46	Average score of impulsiveness.	Score Range: 0-4
NEO PI-R Neuroticism: Vulnerability	NEONeurVuln46	Average score of vulnerability.	Score Range: 0-4
NEO PI-R Extraversion: Warmth	NEOExtWarm46	Average score of warmth.	Score Range: 0-4

NEO PI-R Extraversion: Gregariousness	NEOExtGreg46	Average score of gregariousness.	Score Range: 0-4
NEO PI-R Extraversion: Assertiveness	NEOExtAssert46	Average score of assertiveness.	Score Range: 0-4
NEO PI-R Extraversion: Activity	NEOExtActiv46	Average score of activity.	Score Range: 0-4
NEO PI-R Extraversion: Excitement-seeking	NEOExtExcit46	Average score of excitement-seeking.	Score Range: 0-4
NEO PI-R Extraversion: Positive emotions	NEOExtPosEmo46	Average score of positive emotions.	Score Range: 0-4
NEO PI-R Openness: Fantasy	NEOOpenFant46	Average score of openness to fantasy.	Score Range: 0-4
NEO PI-R Openness: Aesthetics	NEOOpenAest46	Average score of openness to aesthetics.	Score Range: 0-4
NEO PI-R Openness: Feelings	NEOOpenFeel46	Average score of openness about feelings.	Score Range: 0-4
NEO PI-R Openness: Actions	NEOOpenAct46	Average score of openness to actions.	Score Range: 0-4
NEO PI-R Openness: Ideas	NEOOpenIdea46	Average score of openness to ideas.	Score Range: 0-4
NEO PI-R Openness: Values	NEOOpenValue46	Average score of openness to values.	Score Range: 0-4
NEO PI-R Agreeableness: Trust	NEOAgreeTrust46	Average score of trust.	Score Range: 0-4
NEO PI-R Agreeableness: Straightforwardness	NEOAgreeStrait46	Average score of straightforwardness.	Score Range: 0-4
NEO PI-R Agreeableness: Altruism	NEOAgreeAltur46	Average score of altruism.	Score Range: 0-4
NEO PI-R Agreeableness: Compliance	NEOAgreeCompli46	Average score of compliance.	Score Range: 0-4
NEO PI-R Agreeableness: Modesty	NEOAgreeMod46	Average score of modesty.	Score Range: 0-4
NEO PI-R Agreeableness: Tender-mindedness	NEOAgreeTend46	Average score of tender-mindedness.	Score Range: 0-4
NEO PI-R Conscientiousness: Competence	NEOConComp46	Average score of competence.	Score Range: 0-4
NEO PI-R Conscientiousness: Order	NEOConOrder46	Average score of order.	Score Range: 0-4
NEO PI-R Conscientiousness: Dutifulness	NEOConDuti46	Average score of dutifulness.	Score Range: 0-4

NEO PI-R Conscientiousness: Achievement striving	NEOConAchieve46	Average score of achievement striving.	Score Range: 0-4
NEO PI-R Conscientiousness: Self-discipline	NEOConSelfDis46	Average score of self-discipline.	Score Range: 0-4
NEO PI-R Conscientiousness: Deliberation	NEOConDelib46	Average score of deliberation.	Score Range: 0-4
NEO PI-R Conscientiousness Total	NEOConTot46	Summary score of conscientiousness, which is a measure of effective planning, organization, and impulse control.	Score Range: 0-4
NEO PI-R Agreeableness Total	NEOAgreeTot46	Summary score of agreeableness or how trusting, altruistic, and cooperative one is with others.	Score Range: 0-4
NEO PI-R Neuroticism Total	NEONeurTot46	Summary score of neuroticism, which is a measure of emotional instability and degree of negative emotions.	Score Range: 0-4
NEO PI-R Extraversion Total	NEOExtTot46	Summary score of extraversion, which is a measure of how sociable, assertive, outgoing, and excitement-seeking one is.	Score Range: 0-4
NEO PI-R Openness Total	NEOOpenTot46	Summary score of openness to new experiences and learning new information.	Score Range: 0-4
Assessment 47—Big-Five Inventory	Assess47	1 = Has data<br>2 = Assessment data partial<br>3 = No data	
Big-Five Inventory Extraversion	Big5Ext47	Average score of extraversion, which is a measure of how sociable, assertive, outgoing, and excitement-seeking one is.	Score Range: 1-5
Big-Five Inventory Agreeableness	Big5Agree47	Average score of agreeableness, or how trusting, altruistic, and cooperative one is with others.	Score Range: 1-5
Big-Five Inventory Conscientiousness	Big5Con47	Average score of conscientiousness, which is a measure of how effective one is in planning, organization, and impulse control.	Score Range: 1-5
Big-Five Inventory Neuroticism	Big5Neur47	Average score of neuroticism, which is a	Score Range: 1-5

		measure of how emotional instable one is and their degree of negative emotions.	
Big-Five Inventory Openness	Big5Open47	Average score of openness to new experiences and learning new information. Higher scores denote greater openness to new experience and learning new information.	Score Range: 1-5
Assessment 48—Personality Survey	Assess48	1 = Has data<br>2 = Assessment data partial<br>3 = No data	
Personality Survey Orderliness	PersonOrder48	Average score of orderliness. Higher scores indicate greater orderliness.	Score Range: 1-5
Personality Survey Virtue	PersonVirtue48	Average score of virtue. Higher scores indicate greater virtue.	Score Range: 1-5
Personality Survey Traditionalism	PersonTrad48	Average score of traditionalism. Higher scores indicate greater traditionalism.	Score Range: 1-5
Personality Survey Self-Control	PersonSelfCont48	Average score of self-control. Higher scores indicate greater self-control.	Score Range: 1-5
Personality Survey Responsibility	PersonRespon48	Average score of responsibility. Higher scores indicate greater responsibility.	Score Range: 1-5
Personality Survey Industriousness	PersonIndust48	Average score of industriousness. Higher scores indicate greater industriousness.	Score Range: 1-5
Personality Survey Intellect	PersonIntel48	Average score of intellect. Higher scores indicate greater intellect.	Score Range: 1-5
Personality Survey Ingenuity	PersonIngen48	Average score of ingenuity. Higher scores indicate greater ingenuity.	Score Range: 1-5
Personality Survey Competence	PersonComp48	Average score of competence. Higher scores indicate greater competence.	Score Range: 1-5
Personality Survey Quickness	PersonQuick48	Average score of quickness. Higher scores indicate greater quickness.	Score Range: 1-5
Personality Survey Creativity	PersonCreat48	Average score of creativity. Higher scores indicate greater creativity.	Score Range: 1-5

**Assessment** 49—NIH Toolbox Emotion Assessment	Assess49	1 = Has data<br>2 = Assessment data partial<br>3 = No data	
NIH Toolbox Anger Affect Raw	NIHAngerAffCatRaw49	Raw scale score	Score Range: 0-30
NIH Toolbox Anger Affect Theta	NIHAngerAffCatTheta49	Theta score	Score Range: -4-4
NIH Toolbox Anger Affect T-Score	NIHAngerAffCatT49	t-score comparing the test-taker to those in the NIH Toolbox nationally representative normative sample	Mean = 50, Standard Deviation = 10
NIH Toolbox Anger Affect SE	NIHAngerAffCatSE49	Standard error of score	Score Range: 1-8
NIH Toolbox Anger-Hostility Raw	NIHAngerHostFfRaw49	Raw scale score	Score Range: 0-30
NIH Toolbox Anger-Hostility Theta	NIHAngerHostFfTheta49	Theta score	Score Range: -4-4
NIH Toolbox Anger-Hostility T-Score	NIHAngerHostFfT49	t-score comparing the test-taker to those in the NIH Toolbox nationally representative normative sample	Mean = 50, Standard Deviation = 10
NIH Toolbox Anger-Hostility SE	NIHAngerHostFfSE49	Standard error of score	Score Range: 1-8
NIH Toolbox Anger-Physical Aggression Raw	NIHAngerPAFfRaw49	Raw scale score	Score Range: 0-35
NIH Toolbox Anger-Physical Aggression Theta	NIHAngerPAFfTheta49	Theta score	Score Range: -4-4
NIH Toolbox Anger-Physical Aggression T-Score	NIHAngerPAFfT49	t-score comparing the test-taker to those in the NIH Toolbox nationally representative normative sample	Mean = 50, Standard Deviation = 10
NIH Toolbox Anger-Physical Aggression SE	NIHAngerPAFfSE49	Standard error of score	Score Range: 1-8
NIH Toolbox Emotional Support Raw	NIHEmoSupportFfRaw49	Raw scale score	Score Range: 0-40
NIH Toolbox Emotional Support Theta	NIHEmoSupportFfTheta49	Theta score	Score Range: -4-4
NIH Toolbox Emotional Support T-Score	NIHEmoSupportFfT49	t-score comparing the test-taker to those in the NIH Toolbox nationally representative normative sample	Mean = 50, Standard Deviation = 10
NIH Toolbox Emotional Support SE	NIHEmoSupportFfSE49	Standard error of score	Score Range: 1-8

NIH Toolbox Fear-Affect CAT Raw	NIHFearAffCatRaw49	Raw scale score	Score Range: 0-30
NIH Toolbox Fear-Affect CAT Theta	NIHFearAffCatTheta49	Theta score	Score Range: -4-4
NIH Toolbox Fear-Affect CAT T-Score	NIHFearAffCatT49	t-score comparing the test-taker to those in the NIH Toolbox nationally representative normative sample	Mean = 50, Standard Deviation = 10
NIH Toolbox Fear-Affect CAT SE	NIHFearAffCatSE49	Standard error of score	Score Range: 1-8
NIH Toolbox Fear-Somatic Arousal Raw	NIHFearArousalFfRaw49	Raw scale score	Score Range: 0-30
NIH Toolbox Fear-Somatic Arousal Theta	NIHFearArousalFfTheta49	Theta score	Score Range: -4-4
NIH Toolbox Fear-Somatic Arousal T-Score	NIHFearArousalFfT49	t-score comparing the test-taker to those in the NIH Toolbox nationally representative normative sample	Mean = 50, Standard Deviation = 10
NIH Toolbox Fear-Somatic Arousal SE	NIHFearArousalFfSE49	Standard error of score	Score Range: 1-8
NIH Toolbox Friendship FF Raw	NIHFriendFfRaw49	Raw scale score	Score Range: 0-40
NIH Toolbox Friendship FF Theta	NIHFriendFfTheta49	Theta score	Score Range: -4-4
NIH Toolbox Friendship FF T-Score	NIHFriendFfT49	t-score comparing the test-taker to those in the NIH Toolbox nationally representative normative sample	Mean = 50, Standard Deviation = 10
NIH Toolbox Friendship FF SE	NIHFriendFfSE49	Standard error of score	Score Range: 1-8
NIH Toolbox General Life Satisfaction CAT Raw	NIHGenLifeSatCatRaw49	Raw scale score	Score Range: 0-60
NIH Toolbox General Life Satisfaction CAT Theta	NIHGenLifeSatCatTheta49	Theta score	Score Range: -4-4
NIH Toolbox General Life Satisfaction CAT T-Score	NIHGenLifeSatCatT49	t-score comparing the test-taker to those in the NIH Toolbox nationally representative normative sample	Mean = 50, Standard Deviation = 10
NIH Toolbox General Life Satisfaction CAT SE	NIHGenLifeSatCatSE49	Standard error of score	Score Range: 1-8

NIH Toolbox Instrumental Support FF Raw	NIHInstSupportFfRaw49	Raw scale score	Score Range: 0-40
NIH Toolbox Instrumental Support FF Theta	NIHInstSupportFfTheta49	Theta score	Score Range: -4-4
NIH Toolbox Instrumental Support FF T-Score	NIHInstSupportFfT49	t-score comparing the test-taker to those in the NIH Toolbox nationally representative normative sample	Mean = 50, Standard Deviation = 10
NIH Toolbox Instrumental Support FF SE	NIHInstSupportFfSE49	Standard error of score	Score Range: 1-8
NIH Toolbox Loneliness FF Raw	NIHLonelinessFfRaw49	Raw scale score	Score Range: 0-30
NIH Toolbox Loneliness FF Theta	NIHLonelinessFfTheta49	Theta score	Score Range: -4-4
NIH Toolbox Loneliness FF T-Score	NIHLonelinessFfT49	t-score comparing the test-taker to those in the NIH Toolbox nationally representative normative sample	Mean = 50, Standard Deviation = 10
NIH Toolbox Loneliness FF SE	NIHLonelinessFfSE49	Standard error of score	Score Range: 1-8
NIH Toolbox Meaning and Purpose CAT Raw	NIHMeaningPurpCatRaw49	Raw scale score	Score Range: 0-60
NIH Toolbox Meaning and Purpose CAT Theta	NIHMeaningPurpCatTheta49	Theta score	Score Range: -4-4
NIH Toolbox Meaning and Purpose CAT T-Score	NIHMeaningPurpCatT49	t-score comparing the test-taker to those in the NIH Toolbox nationally representative normative sample	Mean = 50, Standard Deviation = 10
NIH Toolbox Meaning and Purpose CAT SE	NIHMeaningPurpCatSE49	Standard error of score	Score Range: 1-8
NIH Toolbox Perceived Hostility FF Raw	NIHPerHostFfRaw49	Raw scale score	Score Range: 0-35
NIH Toolbox Perceived Hostility FF Theta	NIHPerHostFfTheta49	Theta score	Score Range: -4-4
NIH Toolbox Perceived Hostility FF T-Score	NIHPerHostFfT49	t-score comparing the test-taker to those in the NIH Toolbox nationally representative normative sample	Mean = 50, Standard Deviation = 10

NIH Toolbox Perceived Hostility FF SE	NIHPerHostFfSE49	Standard error of score	Score Range: 1-8
NIH Toolbox Perceived Rejection FF Raw	NIHPerRejectFfRaw49	Raw scale score	Score Range: 0-30
NIH Toolbox Perceived Rejection FF Theta	NIHPerRejectFfTheta49	Theta score	Score Range: -4-4
NIH Toolbox Perceived Rejection FF T-Score	NIHPerRejectFfT49	t-score comparing the test-taker to those in the NIH Toolbox nationally representative normative sample	Mean = 50, Standard Deviation = 10
NIH Toolbox Perceived Rejection FF SE	NIHPerRejectFfSE49	Standard error of score	Score Range: 1-8
NIH Toolbox Perceived Stress FF Raw	NIHPerStressFfRaw49	Raw scale score	Score Range: 0-45
NIH Toolbox Perceived Stress FF Theta	NIHPerStressFfTheta49	Theta score	Score Range: -4-4
NIH Toolbox Perceived Stress FF T-Score	NIHPerStressFfT49	t-score comparing the test-taker to those in the NIH Toolbox nationally representative normative sample	Mean = 50, Standard Deviation = 10
NIH Toolbox Perceived Stress FF SE	NIHPerStressFfSE49	Standard error of score	Score Range: 1-8
NIH Toolbox Positive Affect CAT Raw	NIHPosAffectCatRaw49	Raw scale score	Score Range: 0-60
NIH Toolbox Positive Affect CAT Theta	NIHPosAffectCatTheta49	Theta score	Score Range: -4-4
NIH Toolbox Positive Affect CAT T-Score	NIHPosAffectCatT49	t-score comparing the test-taker to those in the NIH Toolbox nationally representative normative sample	Mean = 50, Standard Deviation = 10
NIH Toolbox Positive Affect CAT SE	NIHPosAffectCatSE49	Standard error of score	Score Range: 1-8
NIH Toolbox Sadness CAT Raw	NIHSadnessCatRaw49	Raw scale score	Score Range: 0-30
NIH Toolbox Sadness CAT Theta	NIHSadnessCatTheta49	Theta score	Score Range: -4-4
NIH Toolbox Sadness CAT T-Score	NIHSadnessCatT49	t-score comparing the test-taker to those in the NIH Toolbox nationally	Mean = 50, Standard Deviation = 10

		representative normative sample	
NIH Toolbox Sadness CAT SE	NIHSadnessCatSE49	Standard error of score	Score Range: 1-8
NIH Toolbox Self-Efficacy CAT Raw	NIHSelfEfficCatRaw49	Raw scale score	Score Range: 0-40
NIH Toolbox Self-Efficacy CAT Theta	NIHSelfEfficCatTheta49	Theta score	Score Range: -4-4
NIH Toolbox Self-Efficacy CAT T-Score	NIHSelfEfficCatT49	t-score comparing the test-taker to those in the NIH Toolbox nationally representative normative sample	Mean = 50, Standard Deviation = 10
NIH Toolbox Self-Efficacy CAT SE	NIHSelfEfficCatSE49	Standard error of score	Score Range: 1-8
NIH Negative Affect Summary Score	NIHNegAffSum49	Negative Affect summary score	Score Range: 10-75
NIH Psychological Well-Being Summary Score	NIHWellBeingSum49	Psychological Well-Being summary score	Score Range: 10-75
NIH Social Satisfaction Summary Score	NIHSocialSatSum49	Social Satisfaction summary score	Score Range: 10-75
Assessment 50—Scale of Positive and Negative Emotions	Assess50	1 = Has data<br>2 = Assessment data partial<br>3 = No task data	
Scale of Positive and Negative Emotions: Positive Score	SPANEPos50	A summed score indicating the amount of positive emotions experienced in the past four weeks.	Score Range: 6-30
Scale of Positive and Negative Emotions: Negative Score	SPANENeg50	A summed score indicating the amount of negative emotions experienced in the past four weeks.	Score Range: 6-30
Scale of Positive and Negative Emotions: Balance Score	SPANEBal50	An overall score of emotions experienced in the past four weeks. SPANE Positive total minus SPANE Negative total	Score Range: -24 to 24
Assessment 51—Psychological Well-being	Assess51	1 = Has data<br>2 = Assessment data partial<br>3 = No data	
Psychological Well-being: Positive Relations with Others	SWQRelWOther51	Average score of positive relationships with others, which is the ability to achieve warm, trusting interpersonal relationships.	Score Range: 0-5

		Higher scores indicate greater positive relationship with others.	
Psychological Well-being: Autonomy	SWQAuto51	Average score of autonomy, or self-determination, independence, and evaluating oneself by personal standards instead of outward approval. Higher scores indicate greater autonomy.	Score Range: 0-5
Psychological Well-being: Environmental Mastery	SWQEnvirMast51	Average score of environmental mastery, which is an individual's ability to choose, create, and take advantage of environmental opportunities. Higher scores indicate greater environmental mastery.	Score Range: 0-5
Psychological Well-being: Personal Growth	SWQPersonGrow51	Average score of personal growth, or one's continued commitment to develop as a person. Higher scores indicate greater personal growth.	Score Range: 0-5
Psychological Well-being: Purpose in Life	SWQPurposeLife51	Average score of purpose in life, or a feeling of meaning, sense of directedness, and intentionality. Higher scores indicate greater purpose in life.	Score Range: 0-5
Psychological Well-being: Self-acceptance	SWQSelfAccept51	Average score of self-acceptance or holding positive attitudes towards oneself. Higher scores indicate greater self-acceptance.	Score Range: 0-5
Psychological Well-being Total	SWQTot51	Total average score of psychological well-being. Higher scores are indicative of greater psychological well-being.	Score Range: 1-5

Psychosocial Data Set: Instruments

Assessment 10.39 Martin and Park Environmental Demands (MPED) Questionnaire

1. How busy are you during an average day?
2. How often do you have too many things to do each day to actually get them all done?
3. How often do you find yourself rushing from place to place trying to get appointments or to get things done?
4. How often are you so busy that you miss scheduled breaks or rest periods?
5. How often are you so busy that you miss your regular meal times?
6. How often are you so busy that you forget what you are supposed to do?
7. How often are you so busy that you cannot take you medications when you are supposed to take them?
8. How often do you rush out of the house in the morning to get to where you need to be?
9. How often do you have so many things to do that you go to bed later than you regular bedtime?
10. How often do your days follow a basic routine?
11. How often do you get out of bed in the morning and go to bed at night at about the same time?
12. How often do you eat all of your meals at about the same time each day and night?
13. How often do you engage in activities at home at specific time (i.e. read the paper after work, watch a particular television show, spend time with children, work on hobbies, etc.)?

Assessment 10.40 Daily Activities Questionnaire

1. I prepare a meal
2. I do housework (dishes, laundry, vacuuming, etc.)
3. I go grocery shopping
4. I go shopping at a mall or downtown
5. I drive a car
6. I take a bus
7. I take care of someone in my family (invalid or disabled)
8. I take care of one or more pets
9. I do household repairs (painting, leaky faucets, etc.)
10. I repair a car, lawn mower, or other mechanical device
11. I purchase a new item requiring some set-up or assembly
12. I do woodworking, carpentry, or furniture refinishing
13. I play a musical instrument
14. I engage in creative writing, writing poems, writing newspaper articles, etc.
15. I engage in photography
16. I collect stamps, coins, dolls or other memorabilia
17. I engage in sewing, knitting, or needlework
18. I engage in painting, sculpting, ceramics, drawing, etc.
19. I participate in a theatrical activity
20. I sing in a choir

21. I garden indoors or outdoors
22. I engage in exercise activities such as jogging, swimming, bicycling, or walking
23. I engage in outdoor activities such as sailing, fishing, or backpacking
24. I engage in recreational sports such as tennis, bowling, or golf
25. I work crossword puzzles, acrostics, or anagrams
26. I play card games such as Pinochle or Bridge
27. I do jigsaw puzzles
28. I play board games such as chess and checkers
29. I play knowledge games such as Trivial Pursuit
30. I play word games such as Scrabble
31. I read newspapers
32. I read books or magazines for leisure
33. I read books or magazines as part of my job, career, or formal education
34. I go to the library
35. I watch news programs on television
36. I watch documentary or educational programs on television
37. I watch game shows such as Wheel of Fortune on television
38. I watch comedy or adventure programs on television
39. I watch continuing dramas on television
40. I listen to radio programs
41. I write a letter (to a friend, relative, business, etc.)
42. I program software for a personal computer
43. I use pre-programmed software on a personal computer
44. I use an electronic calculator
45. I balance a check book
46. I prepare my own income taxes
47. I prepare someone else's income taxes
48. I do arithmetic or mathematical calculations
49. I attend films (travel films, commercial movies, etc.)
50. I attend a concert or a play
51. I attend a public lecture or talk
52. I attend sports events such as hockey, soccer, football, baseball, lawn bowling, or cricket
53. I eat out at a restaurant
54. I visit a physician, dentist, or other professional
55. I visit relatives, friends or neighbors
56. I give a dinner or a party for friends
57. I attend religious services
58. I engage in prayer, meditation, or philosophical contemplation
59. I attend meetings of service organizations such as Lions, Rotary, or Seniors Serving Seniors
60. I attend meeting of clubs (hobby club, book club, discussion club, etc.)
61. I give a public talk or lecture (to a club, service organization, etc.)
62. I do volunteer work for an organization such as a hospital, church, school, or political party
63. I engage in business activities such as investments or real estate transactions not related to my job or career

64. I engage in an on-the-job training program
65. I enroll in a course at a college or university
66. I enroll in a correspondence course
67. I study or practice a language other than my native tongue
68. I travel away from my home, but within my home state
69. I travel outside my home state, but within the United States:
70. I travel in a foreign country

Assessment 10.41 Lifetime Cognitive Activities

1. At age 6 how often did someone in your home read to you?
2. At age 6 how often did you play games (i.e. Cards, puzzles, checkers)?
3. At age 6 how often did someone in your home tell you stories?
4. At age 12 how often did you visit the library
5. At age 12 how often did you read newspapers?
6. At age 12 how often did you read magazines?
7. Age at 12 how often did you read books?
8. At age 12 how often did you write letters/ emails?
9. At age 12 how often did you play games (i.e. cards, puzzles, checkers)?
10. At age 18 how often did you visit the library?
11. At age 18 how often did you read newspapers?
12. At age 18 how often did you read magazines?
13. At age 18 how often did you read books?
14. At age 18 how often did you write letters/emails?
15. At age 18 how often did you play games (i.e. cards, puzzles, checkers)?
16. Are you age 41 or older?
17. At age 40 how often did you read newspapers?
18. At age 40 how often did you read magazines?
19. At age 40 how often did you read books?
20. At age 40 how often did you write letters/emails?
21. At age 40 how often did you play games (i.e. cards, puzzles, checkers)?
22. Currently, how often do you read newspapers?
23. Currently, how often do you read magazines?
24. Currently, how often do you read books?
25. Currently, how often do you write letters/emails?
26. Currently, how often do you play games (i.e. cards, puzzles, checkers)?

Assessment 10.42 Need for Cognition Survey (NFC)

1. I prefer complex to simple problems.
2. I like to have the responsibility of handling a situation that requires a lot of thinking.
3. Thinking is not my idea of fun.
4. I would rather do something that requires little thought than something that is sure to challenge m abilities.
5. I try to anticipate and avoid situations where there is a likely chance I will have to think in depth about something.
6. I find satisfaction in deliberation for long hours.
7. I only think as hard as I have to.

8. I prefer to think about small daily projects rather than long-term ones.
9. I like tasks that require little thought once I've learned them.
10. The idea of relying on thought to make my own way to the top appeals to me.
11. I really enjoy a task that involves coming up with new solutions to problems.
12. Learning new ways to think doesn't excite me much.
13. I prefer my life to be filled with problems that I must solve.
14. The notion of thinking abstractly is appealing to me.
15. I would prefer a task that is intellectual, difficult, and important to one that is somewhat important but does not require much thought.
16. I feel relief rather than satisfaction after completing a task that requires a lot of mental effort.
17. It's enough for me that something gets the job done; I don't care how or why it works.
18. I usually end up deliberating about issues even when they do not affect me personally.

Assessment 10.43 Metamemory in Adulthood (MIA) Questionnaire

Different people use their memory in different ways in their everyday lives. For example, some people make shopping lists, whereas others do not. Some people are good at remembering names, whereas others are not. In this questionnaire, we would like you to tell us how you use your memory and how you feel about it. There are no right or wrong answers to these questions because people are different. Please take your time and answer each of these questions to the best of your ability.

1. For most people, facts that are interesting are easier to remember than facts that are not.
2. I am good at remembering names.
3. Do you keep a list of otherwise not important dates, such as birthdays or anniversaries?
4. It is important to me to have a good memory.
5. I get upset when I cannot remember something.
6. When you are looking for something you have recently misplaced, do you try to retrace your steps to locate it?
7. I think a good memory is something of which to be proud.
8. I find it harder to remember things when I am upset.
9. I am good at remembering birthdates.
10. I can remember things as well as always.
11. When you have not finished reading a book or magazine, do you somehow note the place where you have stopped?
12. I get anxious when I am asked to remember something.
13. It bothers me when others notice my memory failures.
14. I'm less efficient at remembering things now than I used to be.
15. I have difficulty remembering things when I'm anxious.
16. The older I get the harder it is to remember clearly.
17. Do you think about the day's activities at the beginning of the day so you can remember what you are supposed to do?
18. I am just as good at remembering as I ever was.
19. I have no trouble keeping track of my appointments.
20. For most people, it is easier to remember information they need to use immediately than information they will not use for a long time.

21. Most people find it easier to remember directions to places they want or need to go than to places they know they will never be going.
22. I am usually uneasy when I attempt a problem that requires me to use my memory.
23. I feel jittery if I have to introduce someone I just met.
24. Having a better memory would be nice but it is not very important.
25. Do you post reminders of things you need to do in a prominent place, such as on bulletin boards or note boards?
26. It does not bother me when my memory fails.
27. I am poor at remembering trivia.
28. I am much worse now at remembering the content of news articles and broadcasts than I was 10 years ago.
29. Do you routinely keep things in a familiar spot so you won't forget them when you need to locate them?
30. Compared to 10 years ago, I am much worse at remembering titles of books, films, or plays.
31. For most people it is easier to remember words they want to use than words they know they will never use.
32. I remember my dreams much less now than 10 years ago.
33. I can't expect to be good at remembering zip codes at my age.
34. Most people find it easier to remember the names of people they especially dislike than people they hardly notice.
35. I have little control over my memory ability.
36. When you want to take something with you, do you leave it in an obvious, prominent place, such as putting your suitcase in front of the door?
37. I think it is important to work at sustaining my memory abilities.
38. I misplace things more frequently now than when I was younger.
39. As people get older they tend to forget where they put things more frequently.
40. I work hard at trying to improve my memory.
41. Compared to 10 years ago, I now forget many more appointments.
42. If I am put on the spot to remember names, I know I will have difficulty doing it.
43. For most people, it is easier to remember the names of people they especially like than people that don't mean very much to them.
44. Most people find it easier to remember words they understand than words that don't mean very much to them.
45. My memory for important events has improved over the last 10 years.
46. I admire people who have good memories.
47. My friends often notice my memory ability.
48. When you try to remember people you have met, do you associate names and faces?
49. I am good at remembering the order that events occurred.
50. For most people, words they have seen or heard before are easier to remember than words that are totally new to them.
51. Familiar things are easier to remember than unfamiliar things.
52. I am good at remembering conversations I have had.
53. I would feel on edge right now if I had to take a memory test or something similar.
54. My memory for phone numbers will decline as I get older.
55. I often notice my friends' memory ability.

56. My memory for dates has greatly declined in the last 10 years.
57. When you have trouble remembering something, do you try to remember something similar in order to help you remember?
58. My memory for names has declined greatly in the last 10 years.
59. I often forget who was with me at events I have attended.
60. Do you consciously attempt to reconstruct the day's events in order to remember something?
61. As long as I exercise my memory, it will not decline.
62. I am good at remembering the places I have been.
63. I know if I keep using my memory I will never lose it.
64. Do you try to relate something you want to remember to something else, hoping that this will increase the likelihood of your remembering later?
65. It's important that I am very accurate when remembering names of people.
66. When I am tense and uneasy at a social gathering, I cannot remember names very well.
67. Do you try to concentrate hard on something you want to remember?
68. It's important that I am very accurate when remembering significant dates.
69. It's up to me to keep my remembering abilities from deteriorating.
70. When someone I don't know very well asks me to remember something, I get nervous.
71. I have no trouble remembering where I have put things.
72. It is easier for most people to remember things that are unrelated to each other than things that are related.
73. Even if I work on it, my memory ability will go downhill.
74. Most people find it easier to remember concrete things than abstract things.
75. Do you make mental images or pictures to help you remember?
76. I know of someone in my family whose memory improved significantly in old age.
77. I am good at remembering things like recipes.
78. I get anxious when I have to do something I haven't done for a long time.
79. It bothers me when I forget an appointment.
80. Most people find it easier to remember things that happen to them than things that happen to others.
81. Do you mentally repeat something you are trying to remember?
82. My memory has improved greatly in the past 10 years.
83. I like to remember things on my own, without relying on other people to remind me.
84. I get tense and anxious when I feel my memory is not as good as other people's.
85. Do you ask other people the remind you of something?
86. I'm highly motivated to remember new things I learn.
87. I do not get flustered when I am put on the spot to remember new things.
88. I am good at remembering titles of books, films, or plays.
89. My memory has declined greatly in the last 10 years.
90. For most people it is easier to remember things in which they are most interested than things in which they are less interested.
91. I have no trouble remembering lyrics of songs.
92. My memory will get better as I get older.
93. It is easier for most people to remember bizarre things than usual things.
94. Do you write yourself reminder notes?
95. I am good at remembering names of musical selections.

96. Most people find it easier to remember visual things than verbal things.
97. After I have read a book, I have no difficulty remembering factual information from it.
98. Do you write appointments on a calendar to help you remember them?
99. I would feel very anxious if I visited a new place and had to remember how to find my way back.
100. I am good at remembering the content of news articles and broadcasts.
101. No matter how hard a person works on his memory, it cannot be improved very much.
102. If I were to work on my memory I could improve it.
103. It gives me great satisfaction to remember things I thought I had forgotten.
104. Remembering the plots of stories and novels is easy for me.
105. I am usually able to remember exactly where I read or heard a specific thing.
106. I think a good memory comes mostly from working at it.
107. Most people find it easier to remember unorganized things than organized things.
108. Do you write shopping lists?

Assessment 10.44 Self-Concept Clarity (SCC) Survey

This survey asks for your views about yourself. For each question, choose the response that best describes your answer.

1. My beliefs about myself often conflict with one another.
2. On one day I might have one opinion of myself and on another day I might have a different opinion.
3. I spend a lot of time wondering what kind of person I really am.
4. Sometimes, I feel that I am not really the person that I appear to be.
5. When I think about the kind of person I have been in the past, I'm not sure what I was really like.
6. I seldom experience conflict between the different aspects of my personality.
7. Sometimes I think I know other people better than I know myself.
8. My beliefs about myself seem to change very frequently.
9. If I were asked to describe my personality, my description might end up being different from one day to another.
10. Even if I wanted to, I don't think I could tell someone what I'm really like.
11. In general, I have a clear sense of who I am and what I am.
12. It is often hard for me to make up my mind about things because I don't really know what I want.

Assessment 10.45 Satisfaction with Life Scale

This survey asks for your views about your life. For each question, choose the response that best describes your answer.

1. In most ways my life is close to my ideal.
2. The conditions of my life are excellent.
3. I am satisfied with my life.
4. So far I have gotten the important things I want in life.
5. If I could live my life over, I would change almost nothing.

Assessment 10.46 Revised Neuroticism-Extraversion-Openness Personality Inventory (NEO-PI-R)

Please refer to publications for questionnaires.

Assessment 10.47 Big 5 Inventory

Here are a number of characteristics that may or may not apply to you. For example, do you agree that you are someone who likes to spend time with others? Please indicate the extent to which you agree or disagree with each statement.

1. Is talkative
2. Tends to find fault with others
3. Does a thorough job
4. Is depressed, blue
5. Is original, comes up with new ideas
6. Is reserved
7. Is helpful and unselfish with others
8. Can be somewhat careless
9. Is relaxed, handles stress well
10. Is curious about many different things
11. Is full of energy
12. Starts quarrels with others
13. Is a reliable worker
14. Can be tense
15. Is ingenious, a deep thinker
16. Generates a lot of enthusiasm
17. Has a forgiving nature
18. Tends to be disorganized
19. Worries a lot
20. Has an active imagination
21. Tends to be quiet
22. Is generally trusting
23. Tends to be lazy
24. Is emotionally stable, not easily upset
25. Is inventive
26. Has an assertive personality
27. Can be cold and aloof
28. Perseveres until the task is finished
29. Can be moody
30. Values artistic, aesthetic experiences
31. Is sometimes shy, inhibited
32. Is considerate and kind to almost everyone
33. Does things efficiently
34. Remains calm in tense situations
35. Prefers work that is routine
36. Is outgoing, sociable
37. Is sometimes rude to others
38. Makes plans and follows through with them

39. Gets nervous easily
40. Likes to reflect, play with ideas
41. Has few artistic interests
42. Likes to cooperate with others
43. Is easily distracted
44. Is sophisticated in art, music, or literature

Assessment 10.48 Personality Survey

On the following pages, there are phrases describing people's behaviors. Please describe yourself as you generally are now, not as you wish to be in the future. Describe yourself as you honestly see yourself, in relation to other people you know of your gender and age. Please read each statement carefully, and select the response that best describes your answer.

1. Being neat is not exactly my strength.
2. Organization is a key component of most things I do.
3. I need a neat environment in order to work well.
4. I become annoyed when things around me are disorganized.
5. For me, being organized is unimportant.
6. Half of the time I do not put things in their proper place.
7. Most of the time my room is in complete disarray.
8. Every item in my room and on my desk has its own designated place.
9. I frequently forget to put things back in their proper place.
10. I hate when people are sloppy.
11. If I could get away with it, I would not pay taxes.
12. I would lie without hesitation if it serves my purpose.
13. I could be insincere and dishonest if situation required me to do so.
14. If I find money laying around, I'll keep it to myself.:
15. If I cashier forgot to charge me for an item I would tell him/her.
16. I would rather get a bad grade than copy someone else's homework and turn it in as my own.
17. It bothers me when people cheat on their taxes.
18. If I accidentally scratched a parked car, I would try to find the owner to pay for the repairs.
19. I firmly believe that under no circumstances it is okay to lie.
20. The people who know me best would say that I am honest.
21. I have the highest respect for authorities and assist them whenever I can.
22. People respect authority more than they should.
23. Even if I knew how to get around the rules without breaking them, I would not do it.
24. I believe that people should be allowed to take drugs, as long as it doesn't affect others.
25. I support long-established rules and traditions.
26. People who resist authority should be severely punished.
27. When I was in school, I used to break rules quite regularly.
28. In my opinion, all laws should be strictly enforced.
29. In my opinion, censorship slows down progress.
30. When working with others I am the one who makes sure that rules are observed.
31. I often rush into action without thinking about potential consequences.
32. I rarely jump into something without first thinking about it.

33. I am known to make quick, hot-headed decisions.
34. I do not take unnecessary risks.
35. I am easily talked into doing silly things.
36. My friends say I am unpredictable.
37. I get into trouble because I act on impulses rather than on thoughts.
38. I am careful with what I say to others.
39. I dislike being around impulsive people.
40. Even under time pressure, I would rather take my time to think about my answer than to say the first thing that comes to mind.
41. I carry out my obligations to the best of my ability.
42. I often feel responsible for making sure that all group project assignments are completed.
43. I go out of my way to keep my promises.
44. Sometimes it is too much of a bother to do exactly what is promised.
45. I would gladly spend some of my leisure time trying to improve my community.
46. If I am running late to an appointment, I may decide not to go at all.
47. I am usually not the most responsible group member, but I will not shirk on my duties either.
48. If I am running late, I try to call ahead to notify those who are waiting for me.
49. When I make mistakes I often blame others.
50. I have a reputation for being late for almost every meeting or event.
51. I have high standards and work toward them.
52. I go above and beyond of what is required.
53. I do not work as hard as the majority of people around me.
54. I invest little effort into my work.
55. I demand the highest quality in everything I do.
56. I try to be the best at anything I do.
57. I make every effort to do more than what is expected of me.
58. I do what is required, but rarely anything more.
59. Setting goals and achieving them is not very important to me.
60. Getting average grades is enough for me.
61. I have a rich vocabulary.
62. I use difficult words.
63. I make insightful remarks.
64. I show a mastery of language.
65. I enjoy thinking about things.
66. I try to understand myself.
67. I am not interested in abstract ideas.
68. I will not probe deeply into a subject.
69. I have a poor vocabulary.
70. I dislike learning.
71. I skip difficult words while reading.
72. I am full of ideas.
73. I have excellent ideas.
74. I carry the conversation to a higher level.
75. I come up with bold plans.
76. I quickly think up new ideas.

77. I am good at many things.
78. I do not have a good imagination.
79. I have difficulty imagining things.
80. I can't come up with new ideas.
81. I learn quickly.
82. I use my brain.
83. I excel in what I do.
84. I look at the facts.
85. I meet challenges.
86. I seek explanations of things.
87. I need things explained only once.
88. I know how to apply my knowledge.
89. I can handle complex problems.
90. I am quick to understand things.
91. I catch on to things quickly.
92. I love to read challenging material.
93. I am able to find out things by myself.
94. I can handle a lot of information.
95. I quickly get the idea of things.
96. I avoid difficult reading material.
97. I try to avoid complex people.
98. I don't understand things.
99. I like to solve complex problems.
100. I ask questions that nobody else does.
101. I know the answers to many questions.
102. I challenge others' point of view.
103. I can easily link facts together.
104. I have difficulty understanding abstract ideas.
105. I avoid philosophical discussions.
106. I am not interested in theoretical discussions.
107. I consider myself an average person.
108. I am not interested in speculating about things.

Assessment 10.49 NIH Toolbox Emotion Measures

Please refer to publications for questionnaires.

Assessment 10.50 Scale of Positive and Negative Experience (SPANE)

Please think about what you have been doing and experiencing during the past four weeks. Then report how much you experienced each of the following feelings:

1. During the past 4 weeks, I experienced feeling: Positive
2. During the past 4 weeks, I experienced feeling: Negative
3. During the past 4 weeks, I experienced feeling: Good
4. During the past 4 weeks, I experienced feeling: Bad
5. During the past 4 weeks, I experienced feeling: Pleasant
6. During the past 4 weeks, I experienced feeling: Unpleasant
7. During the past 4 weeks, I experienced feeling: Happy

8. During the past 4 weeks, I experienced feeling: Sad
9. During the past 4 weeks, I experienced feeing: Afraid
10. During the past 4 weeks, I experienced feeling: Joyful
11. During the past 4 weeks, I experienced feeling: Angry
12. During the past 4 weeks, I experienced feeling: Contented

Assessment 10.51 Psychological Well-being (SWQ)

This survey asks for your views about yourself. For each question, choose the response that best describes your answer.

1. Most people see me as loving and affectionate.
2. Sometimes I change the way I act or think to be more like those around me.
3. In general, I feel I am in charge of the situation in which I live.
4. I am not interested in activities that will expand my horizons.
5. I feel good when I think of what I've done in the past and what I hope to do in the future.
6. When I look at the story of my life, I am pleased with how things have turned out.
7. Maintaining close relationships has been difficult and frustrating for me.
8. I am not afraid to voice my opinions, even when they are in opposition to the opinions of most people.
9. The demands of everyday life often get me down.
10. In general, I feel that I continue to learn more about myself as time goes by.
11. I live life one day at a time and don't really think about the future.
12. In general, I feel confident and positive about myself.
13. I often feel lonely because I have few close friends with whom to share my concerns.
14. My decisions are not usually influenced by what everyone else is doing.
15. I do not fit very well with the people and the community around me.
16. I am the kind of person who likes to give new things a try.
17. I tend to focus on the present, because the future nearly always brings me problems.
18. I feel like many of the people I know have gotten more out of life than I have.
19. I enjoy personal and mutual conversations with family members or friends.
20. I tend to worry about what other people think of me.
21. I am quite good at managing the many responsibilities of my daily life.
22. I don't want to try new ways of doing things - my life is fine the way it is.
23. I have a sense of direction and purpose in life.
24. Given the opportunity, there are many things about myself that I would change.
25. It is important to me to be a good listener when close friends talk to me about their problems.
26. Being happy with myself is more important to me than having others approve of me.
27. I often feel overwhelmed by my responsibilities.
28. I think it is important to have new experiences that challenge how you think about yourself and the world.
29. My daily activities often seem trivial and unimportant to me.
30. I like most aspects of my personality.
31. I don't have many people who want to listen when I need to talk.
32. I tend to be influenced by people with strong opinions.
33. If I were unhappy with my living situation, I would take effective steps to change it.
34. When I think about it, I haven't really improved much as a person over the years.

35. I don't have a good sense of what it is I'm trying to accomplish in life.
36. I made some mistakes in the past, but I feel that all in all everything has worked out for the best.
37. I feel like I get a lot out of my friendships.
38. People rarely talk me into doing things I don't want to do.
39. I generally do a good job of taking care of my personal finances and affairs.
40. In my view, people of every age are able to continue growing and developing.
41. I used to set goals for myself, but that now seems like a waste of time.
42. In many ways, I feel disappointed about my achievements in life.
43. It seems to me that most other people have more friends than I do.
44. It is more important to me to fit in with others than to stand alone on my principles.
45. I find it stressful that I can't keep up with all of the things I have to do each day.
46. With time, I have gained a lot of insight about life that has made me a stronger, more capable person.
47. I enjoy making plans for the future and working to make them a reality.
48. For the most part, I am proud of who I am and the life I lead.
49. People would describe me as a giving person, willing to share my time with others.
50. I have confidence in my opinions, even if they are contrary to the general consensus.
51. I am good at juggling my time so that I can fit everything in that needs to be done.
52. I have a sense that I have developed a lot as a person over time.
53. I am an active person in carrying out the plans I set for myself.
54. I envy many people for the lives they lead.
55. I have not experienced many warm and trusting relationships with others.
56. It's difficult for me to voice my own opinions on controversial matters.
57. My life is busy, but I derive a sense of satisfaction from keeping up with everything.
58. I do not enjoy being in new situations that require me to change my old familiar ways of doing things.
59. Some people wander aimlessly through life, but I am not one of them.
60. My attitude about myself is probably not as positive as most people feel about themselves.
61. I often feel as if I'm on the outside looking in when it comes to friendships.
62. I often change my mind about decisions if my friends or family disagree.
63. I get frustrated when trying to plan my daily activities because I never accomplish the things I set out to do.
64. For me, life has been a continuous process of learning, changing and growth.
65. I sometimes feel as if I've done all there is to do in life.
66. Many days I wake up feeling discouraged about how I have lived my life.
67. I know that I can trust my friends, and they know they can trust me.
68. I am not the kind of person who gives in to social pressures to think or act in certain ways.
69. My efforts to find the kinds of activities and relationships that I need have been quite successful.
70. I enjoy seeing how my views have changed and matured over the year.
71. My aims in life have been more a source of satisfaction than frustration to me.
72. The past had its ups and down, but in general, I wouldn't want to change it.
73. I find it difficult to really open up when I talk to others.

74. I am concerned about how other people evaluate the choices I have made in my life.
75. I have difficulty arranging my life in a way that is satisfying to me.
76. I gave up trying to make big improvements or changes in my life a long time ago.
77. I find it satisfying to think about what I have accomplished in life.
78. When I compare myself to friends and acquaintances, it makes me feel good about who I am.
79. My friends and I sympathize with each other's problems.
80. I judge myself by what I think is important, not by the values of what others think is important.
81. I have been able to build a home and a lifestyle for myself that is much to my liking.
82. There is truth to the saying that you can't teach an old dog new tricks.
83. In the final analysis, I'm not so sure that my life adds up to much.
84. Everyone has their weaknesses, but I seem to have more than my fair share.
